# Spatiotemporal modeling of GPCR signaling: The role of endosomal dynamics and receptor recycling

**DOI:** 10.1371/journal.pcbi.1014790

**Published:** 2026-09-29

**Authors:** Chloé Weckel, Juliette Gourdon, Léo Darrigade, Vinesh Jugnarain, Pascale Crépieux, Eric Reiter, Frédéric Jean-Alphonse, Stefan Haar, Romain Yvinec

**Affiliations:** 1 Musca, Inria, Université Paris-Saclay, Inria Saclay-Ile-de-France, Palaiseau, France; 2 PRC, INRAE, CNRS, Université de Tours, Nouzilly, France; UFRN: Universidade Federal do Rio Grande do Norte, BRAZIL

## Abstract

A growing number of G Protein-Coupled Receptors (GPCRs) have been reported to initiate signaling both at the cell surface and from intracellular compartments such as endosomes. The kinetics and spatial localization of these signals are critical determinants of cellular responses, yet receptor trafficking - including internalization, endosomal sorting, and recycling - remains a pivotal but often overlooked component of theoretical GPCR models. Here, we present a generic GPCR dynamic model that explicitly integrates receptor trafficking and signaling compartmentalization, enabling systematic characterization of the role of endosomal dynamics and receptor recycling on ligand-induced cellular responses. Then, as a case study, a model selection method with high-throughput kinetic data from the follicle-stimulating hormone receptor (FSHR) is used to reveal the impact of receptor trafficking on FSH-induced signaling responses. Although only a small fraction of FSHRs is internalized, these receptors produce a highly active endosomal response, underscoring the strong effect that any drug perturbing receptor trafficking can have. Our approach thus provides a refined tool for the pharmacological characterization of ligands and advances understanding of the spatial organization of FSHR signaling. Beyond this specific receptor, the methodology offers a generalizable strategy for modeling GPCR trafficking dynamics.

## Author summary

Cell signaling pathways have long been viewed as being activated by interactions between extracellular ligands and a relatively static pools of membrane receptors. However, growing evidence shows that ligand–receptor interactions trigger complex spatial trafficking processes and activate signaling pathways at distinct intracellular locations (e.g., endosomes). This has led to the emergence of a new paradigm of compartmentalized signaling, which remains poorly understood but offers exciting pharmacological opportunities. In this study, we adapt a well-established ordinary differential equation framework to describe cell signaling pathways by explicitly incorporating receptor trafficking and compartmentalized signaling. Our steady-state analysis demonstrates the critical role of both receptor trafficking and compartmentalized signaling in shaping cellular responses, providing further evidence that these mechanisms should be considered in drug design. We then apply a quantitative model selection and calibration approach on the follicle-stimulating hormone receptor (FSHR) using kinetic data on receptor trafficking and signaling dynamics. The FSHR has been characterized to internalize in distinct and peculiar endosomal compartments, whose dynamics remain unclear. Our results reveal dynamic spatial trafficking in three distinct compartments, that strongly influences signaling outcomes. This quantitative framework provides new insights into follicle-stimulating hormone receptor pharmacology, with broader implications for reproductive management in both human health and livestock production.

## Introduction

G protein-coupled receptors (GPCRs) constitute a large family of transmembrane receptors governing essential physiological functions, including reproduction, immune response, metabolism, and cardiovascular homeostasis. These receptors are targeted by a diverse array of ligands, such as hormones, ions, and neurotransmitters. Upon ligand binding, GPCR activation triggers intracellular signaling cascades that ultimately modulate gene expression and biological responses [1]. Given their pivotal role in integrated physiological processes, GPCRs represent a major class of pharmacological targets. The traditional view, according to which signaling cascades are activated at the plasma membrane (PM) and subsequently desensitized via receptor internalization, has evolved substantially. It is now well established that many GPCRs remain active in endosomes after internalization and trigger kinetics cellular responses distinct from those initiated at the plasma membrane [2]. While the physiological significance of this compartmentalized signaling has long been underappreciated, it has been elucidated for a few GPCRs. Endosomal cAMP signaling induced by the parathyroid hormone type 1 receptor (PTHR) specifically drives the synthesis of active vitamin D, increases serum Ca^2+^ levels, and promotes bone formation [3]. Compartmentalized signaling can extend beyond endosomes to deeper intracellular organelles: the TSH receptor (TSHR) undergoes retrograde trafficking to the trans-Golgi network (TGN), where local cAMP/PKA signaling in proximity to the nucleus is required for efficient CREB phosphorylation and gene transcription [4]. The glucagon-like peptide-1 receptor (GLP-1R) provides yet another example, where intracellular trafficking shapes the duration and amplitude of cAMP signaling with direct metabolic consequences [5].

Although most GPCRs signal from two cellular compartments, some receptors have been shown to localize in three distinct compartments. This is the case of the TSHR, but also of the gonadotropin receptors. To date, gonadotropin receptor trafficking remains poorly characterized: the Follicle-Stimulating Hormone Receptor (FSHR) and the Luteinizing Hormone Receptor (LHR) display atypical endosomal distribution [2,6–8]. Unlike many GPCRs internalized directly into Early Endosomes (EE), gonadotropin receptors are preferentially sorted into a distinct, small endosomal compartment known as Very Early Endosomes (VEE), that differ from EE in both size and molecular markers [6,8]. This VEE pathway facilitate rapid receptor recycling to the plasma membrane and is established as a signaling-competent route. The capacity of receptors to sustain signaling from EE remains unclear. In this line, whether EEs and VEEs are each capable of mediating distinct functional roles, or whether signaling is predominantly driven by one or the other compartment, remains an open question. Signaling from endosomal compartments of the luteinizing hormone (LH) receptor has been shown to control the resumption of oocyte meiosis preceding ovulation [9] and gonadal steroidogenesis [10]. As FSHR and LHR play crucial roles in reproduction by supporting steroidogenesis and gametogenesis in both female and male, deciphering the precise spatial organization of their signaling is of both fundamental and therapeutic importance. Understanding cell signaling events and their spatiotemporal dynamics is a key step to develop pharmacological approaches. Since individual processes within GPCR signaling pathways are often experimentally inaccessible, analyzing and estimating parameters for comprehensive models is a crucial step toward characterizing signaling compartmentalization. Hence, mathematical modeling may critically help disentangling each compartment contributions.

Deterministic frameworks based on ordinary differential equations (ODEs), derived from chemical reaction networks and structured through compartmentalized representations, have been developed to investigate spatiotemporal dynamics of signaling [11–13]. In these models, the first signaling compartment typically represents the PM, while subsequent ones may correspond to intracellular structures such as EE, the Golgi apparatus, or the nucleus, depending on the receptor considered [14–17]. In [11], the authors introduced a two-compartment model that incorporates ligand-induced receptor internalization together with receptor recycling, and used it to quantify the accumulation of internalized ligand as a function of extracellular ligand concentration. However, the impact of receptor trafficking on cellular response has not yet been assessed in such a model. Concomitantly, in [12], the authors extended the model of [18] - which integrates ligand-receptor binding, four distinct receptor conformational states, and G-protein activation - by incorporating internalization and recycling dynamics, enabling numerical exploration of drug efficacy and potency across different G-protein subtypes for a same receptor. Furthermore, in [19], the authors proposed a model with seven intracellular compartments, performing a meta-analysis across eight receptor tyrosine kinases (RTKs) to quantify the contribution of each endocytic compartment to receptor signaling. Although these models are biologically interpretable, a full interpretation remains challenging due to the high dimensionality of the system and the large number of compartment-specific parameters [12,19].

Classical pharmacological theory aims to quantify the action of both natural and synthetic ligands at a given receptor. The foundation of this field is the operational model, which draws an analogy between ligand-receptor-effector interactions and enzymatic processes [20]. At steady state, the operational model establishes a relationship between ligand concentration and cellular response, thereby facilitating dose-response analysis. The quantification of ligand efficacy and potency enables the detection of ligand bias, a promising concept for the rational design of drugs with enhanced therapeutic efficacy and reduced adverse effects.  [21] and [22] employ an agonism operational model derived from [20] to quantify biased ligands at a given receptor. Moreover, recent works have highlighted the critical influence of ligand binding kinetics on cellular responses, giving rise to the concept of kinetic bias in pharmacology [23]. The role of receptor trafficking - namely internalization into endosomes and recycling back to the plasma membrane - along with signaling from intracellular compartments, calls for a revision of the standard operational model using compartmental models. Spatial bias, arising from the subcellular localization of receptors, has also been identified as a key determinant of differential signaling outcomes [3]. Notably, it has recently been shown that intracellular receptor distribution - for instance, across distinct endosomal subtypes - can be selectively modulated using innovative pharmacological tools [24]. Previous studies have employed compartmental models to quantify the impact of receptor trafficking on dose-response characteristics - such as efficacy and potency [11,12]. However, those approaches rely on steady-state fitting, whereas a systematic kinetic approach remains essential to fully dissect the underlying mechanisms [19]. Beyond steady-state analyses, Hoare and colleagues [13,25] developed a series of alternative models under different kinetic assumptions to directly fit time-series experimental data using minimal model frameworks. Nevertheless, questions surrounding model selection and parameter identifiability remain largely unaddressed across these studies. General-purpose workflows covering the full parameter estimation pipeline for dynamic systems - from model design to prediction, and encompassing structural identifiability analysis, objective function formulation, and uncertainty quantification - have been proposed to address these challenges [26,27].

The present study aims to elucidate how receptor trafficking shape cellular responses using up-to-date analytic and parameter estimation methods. We built on the two-compartment model of [11] and extended it to a three-compartment framework that more explicitly accounted for distinct compartment types and their mutual interactions, while seeking an appropriate balance between model complexity and interpretability. Using both the two- and three-compartment frameworks, we systematically investigated the role of receptor trafficking and endosomal signaling on cellular responses. This framework was formulated in general terms, exploiting the conserved trafficking mechanisms shared across GPCRs, making it readily applicable to a broad range of receptor systems. Particular attention was given to the characterization of unique steady states in multi-compartment systems, which provides a concise description of cellular responses and support rigorous dose-response analysis. This framework allowed us to quantify the impact of receptor internalization and recycling on second messenger production and their influence on downstream signaling. Inhibiting receptor internalization remains the primary experimental strategy for dissecting the contribution of post-endocytic signaling from that occurring at the plasma membrane [10,28,29]. Thus, through dose-response analyses, we examined how internalization inhibition alters ligand-induced responses, following the approach of [21], and demonstrated how trafficking dynamics and receptor spatial distribution drastically dictate signaling outcomes. By combining experiments with a dedicated mathematical framework, the contributions of receptor trafficking and endosomal signaling to cellular responses were uncovered. To this end, we designed a series of dose-dependent kinetic experiments monitoring the cellular response induced by FSH on the FSHR, with and without blocking receptor internalization, complemented by real-time measurements of receptor internalization and trafficking to EEs. Starting from a generic three-compartment kinetic model, we established a pipeline for model selection and parameter estimation. Special emphasis was placed on parameter identifiability, and model predictions were corroborated using independent validation data. Our results support the hypothesis that the FSHR signals from two distinct endosomal compartments, specifically VEEs and EEs. We further quantified the respective contribution of each compartment to the overall cellular response. Finally, we proposed experimental strategies to further increase predictive accuracy in trafficking parameters.

## Results

### Receptor trafficking and spatial distribution shape cellular responses

In this first part, we systematically investigated the role of receptor trafficking and endosomal signaling on cellular responses, using generic two and three-compartment models of GPCR effectors. We framed it for the cyclic adenosine monophosphate (cAMP) effector, a central second messenger in $G_{\alpha s}$-coupled receptor signaling cascades, that regulates key physiological functions, although other pathways mediated by distinct effectors may also be considered.

#### Receptor trafficking critically modulates both the potency and efficacy of ligand-induced signaling.

To investigate the effect of internalization and recycling on the production of cAMP, we first studied a model with two compartments, inspired from [11]. Model 1 (Fig 1) was developed to include reversible ligand-receptor binding, ligand-receptor internalization and receptor recycling, as well as catalytic cAMP production in both compartments and first-order cAMP degradation. Formulating model 1 (Fig 1) as a system of ordinary differential equations (ODEs) (Methods -Eq. (12)), we described the temporal dynamics of the system and derived its unique steady-state for the cAMP response which reads, as a function of the total quantity of ligand (*L*), receptors (*R*_0_) and model parameters (Eq. (1)) [11]:


$$\begin{array}{c} cAMP(L,R_{0})=\left(\frac{k_{1}^{+}}{k^{-}}+\frac{k_{2}^{+}}{k^{-}}\frac{k_{i12}}{k_{r21}}\right)R_{0}\frac{L}{L\left(1+\frac{k_{i12}}{k_{r21}}\right)+K_{D}\left(1+\frac{k_{i12}}{k_{off}}\right)}, \end{array}$$ (1)

**Fig 1:**
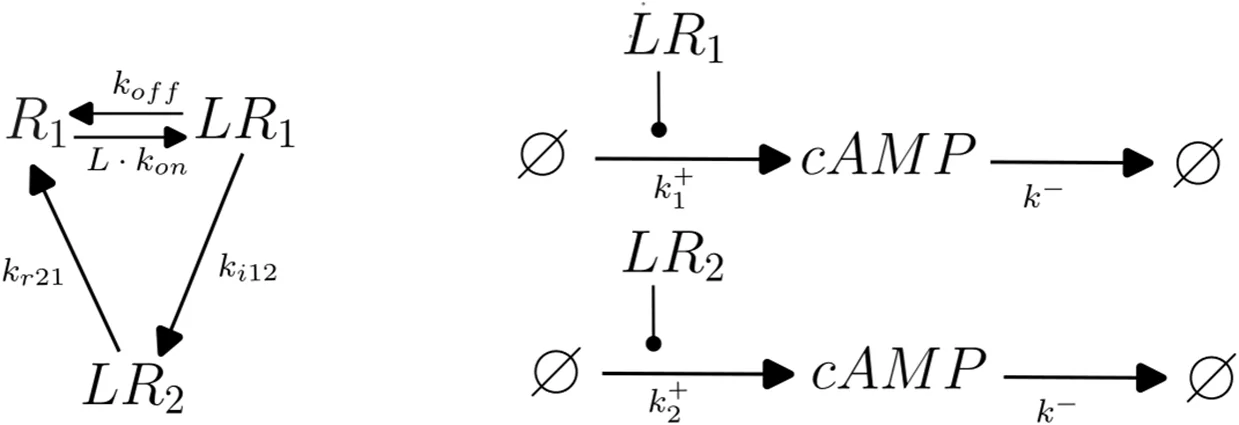
Model 1. The left panel illustrates receptor trafficking, whereas the right panel depicts second messenger production and degradation. *R*_1_ denotes the amount of free receptors at the plasma membrane, *LR*_1_ and *LR*_2_ the amount of ligand-receptor complex at the plasma membrane and intracellular compartments. cAMP refers to the quantity of second messenger molecules. A parameter was associated to each reaction rate (represented by arrows).

with $K_{D}=\frac{k_{off}}{k_{on}}$. All calculations are detailed in Methods. From Eq. (1), we deduced that the efficacy is given by:


$$\begin{array}{c} E_{max}=\frac{\left(\frac{k_{1}^{+}}{k^{-}}+\frac{k_{2}^{+}}{k^{-}}\frac{k_{i12}}{k_{r21}}\right)}{1+\frac{k_{i12}}{k_{r21}}}R_{0}, \end{array}$$ (2)

and the potency is determined by:


$$\begin{array}{c} EC_{50}=\frac{\left(1+\frac{k_{i12}}{k_{off}}\right)}{1+\frac{k_{i12}}{k_{r21}}}K_{D}. \end{array}$$ (3)

To investigate the effect of receptor trafficking, we compared expressions (1)–(3) with the classical ligand-receptor interaction model, when no internalization occurs ($k_{i12}=0$, Eq. (4)):


$$\begin{array}{c} cAMP(L,R_{0})=\frac{k^{+}}{k^{-}}R_{0}\frac{L}{L+K_{D}}, \end{array}$$ (4)

for which $E_{max}=\frac{k^{+}}{k^{-}}R_{0}$ and $EC_{50}=K_{D}$.

We thus deduced that receptor trafficking ($k_{i12},k_{r21}$) modulates both potency and efficacy of the signaling response, whereas second messenger production rates ($k_{1}^{+},k_{2}^{+}$) influence efficacy alone.

We subsequently focused our analysis on scenarios mimicking receptor internalization inhibition. As illustrated in Fig 2, reducing the internalization rate can elicit diametrically opposite effects on the cellular response, depending on the relative magnitudes of signaling ($k_{1}^{+},k_{2}^{+}$) and receptor ($k_{off},k_{r21}$) parameters. When the endocytic response is weaker than the plasma membrane response ($k_{2}^{+}<k_{1}^{+}$), inhibiting receptor internalization increases efficacy ($E_{max}$; Fig 2A and 2B). Conversely, when the endocytic response is stronger than the plasma membrane response ($k_{2}^{+}>k_{1}^{+}$), inhibiting receptor internalization decreases efficacy ($E_{max}$; Fig 2C and 2D).

**Fig 2:**
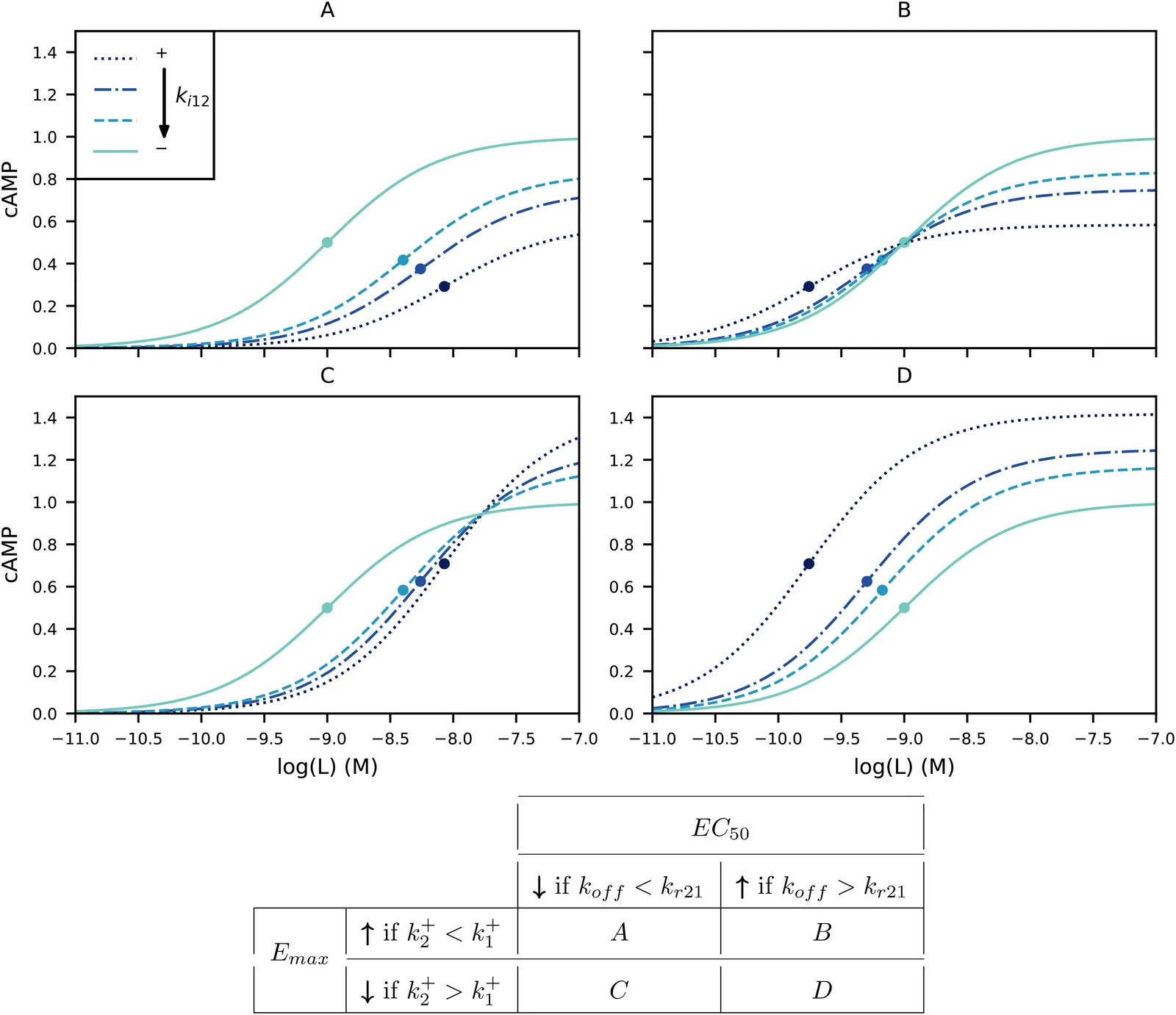
Impact of internalization inhibition on cAMP production using dose-response analysis. In the different panels, the ligand (M) dose response relationship of model 1 (Fig 1) is plotted for different values of the internalization rate: $k_{i12}=5$ (dotted line), $k_{i12}=1$ (dash-dot line), $k_{i12}=0.5$ (dashed line), $k_{i12}=0$ (solid line). Circles represent the *EC*_50_ value. Common parameters to all six panels are $K_{D}=10^{-9}$, *R*_0_ = 1, *k*^–^ = 1, $k_{1}^{+}=1$, $k_{r21}=1$. The remaining parameters values ($k_{2}^{+}$, $k_{off}$) are given by: **(A)** $k_{2}^{+}=0.5$, $k_{off}=0.1$; **(B)** $k_{2}^{+}=0.5$, $k_{off}=100$; **(C)** $k_{2}^{+}=1.5$, $k_{off}=0.1$; **(D)** $k_{2}^{+}=1.5$, $k_{off}=100$. Parameter values were arbitrarily chosen, for illustration purposes.

Similarly, when ligand dissociation from the receptor is faster than receptor recycling ($k_{off}>k_{r21}$), inhibiting receptor internalization increases potency (*EC*_50_; Fig 2B and 2D). In contrast, when ligand dissociation is slower than receptor recycling ($k_{off}<k_{r21}$), inhibiting receptor internalization decreases potency (*EC*_50_; Fig 2A and 2C). Reduction in ligand efficacy (Fig 2C and 2D) is rather expected for receptors that have a strong intracellular activity, as inhibiting receptor internalization will prevent signaling from endosomal compartments. A decrease of ligand potency (Fig 2B and 2D; increase of *EC*_50_) is more subtle, and shows that receptor trafficking may indeed affect the observed affinity from dose-response cellular assays. Going further, while inhibiting receptor internalization, the condition $k_{2}^{+}>k_{1}^{+}$ and $k_{off}>k_{r21}$ (Fig 2D) leads to a globally less efficient response (i.e., $E_{max}$ decreases and *EC*_50_ increases), and symmetrically the condition $k_{2}^{+}<k_{1}^{+}$ and $k_{off}<k_{r21}$ (Fig 2A) leads to a globally more efficient response (i.e., $E_{max}$ increases and *EC*_50_ decreases). However, as shown in Fig 2B and 2C, the effect of inhibiting receptor internalization may be more ambiguous. In these scenarios, efficacy and potency are affected in opposite directions. To resolve the complexities of cases (b) and (c) and characterize overall signaling efficiency under internalization blockade, we calculated the transducer ratio τ (Eq. (5)), which defines coupling efficiency as the ratio of efficacy to potency:


$$\begin{array}{c} \tau =\frac{E_{max}}{EC_{50}}=\frac{R_{0}}{K_{D}}\frac{\frac{k_{1}^{+}}{k^{-}}+\frac{k_{2}^{+}}{k^{-}}\frac{k_{i12}}{k_{r21}}}{\left(1+\frac{k_{i12}}{k_{off}}\right)}. \end{array}$$ (5)

The transducer ratio (Eq. (5)) decreases when inhibiting receptor internalization if $k_{2}^{+}k_{off}>k_{1}^{+}k_{r21}$ (Fig 2D), and increases in the opposite case (Fig 2A). However, the transducer ratio can increase or decrease in both cases (b) and (c). Given the widespread use of the transducer ratio to quantify ligand bias [21], these results further suggest that it is also a reliable proxy for assessing how ligand-dependent responses are altered when internalization is perturbed.

Analytic formula (Eqs. (2)-(3)) further reveal that the relative time scale of trafficking parameters ($k_{i12},k_{r21}$) and ligand unbinding rates ($k_{off})$ affects potency exclusively, leaving efficacy unchanged. Indeed, if the trafficking parameter time scale decreases, with a constant ratio $\frac{k_{i12}}{k_{r21}}$, $E_{max}$ remains unchanged, while the *EC*_50_ decreases. This indicates that, within this model, ligands inducing a slower receptor trafficking will have a tendency to have higher observed potency (Eq. (3)).

#### Spatial distribution of receptors modulates both the potency and efficacy of ligand-induced signaling.

The results derived in Eqs. (1)-(3) can be readily generalized to an arbitrary number of receptor-containing compartments. Beyond receptor trafficking kinetics, the spatial distribution of receptors within the cell may also influence the cellular response. To investigate this, we extended the analysis to model 2 (Fig 3) with three compartments (e.g., very early and early endosomes in the case of gonadotropin receptors). Model 2 (Fig 3) was constructed to account for reversible ligand-receptor binding, ligand-receptor internalization to two different compartments (*LR*_2_ and *LR*_3_), and receptor recycling from both intracellular compartments. It further incorporates compartment maturation, whereby ligand-receptor complexes *LR*_2_ transition to *LR*_3_. As in model 1 (Fig 1), catalytic cAMP production occurs across all compartments, and cAMP degradation follows first-order kinetics. Interpreting again model 2 (Fig 3) as an ODE system (Methods -Eq. (14)), we obtained a unique steady-state for the cAMP response, derived in Methods -Eq. (15), which yields analytical expressions for the pharmacological quantities $E_{max}$ and *EC*_50_ (Methods -Eqs. (16)-(17)). In order to investigate how the spatial distribution of ligand-receptor complexes influences the cellular response, we focused on conditions that promote their accumulation in the third compartment [24]. To this end, we introduced a parameter $p_{23}\in [0,1]$, representing the fraction of internalized receptors directed to *LR*_3_ rather than *LR*_2_. The internalization rates were thus defined as $k_{i12}=(1-p_{23})\cdot k_{int}$ and $k_{i13}=p_{23}\cdot k_{int}$ where $k_{int}$ denotes the total internalization rate. From the analytical formula for the steady-state cAMP response (Methods -Eq. (15)), we obtained the efficacy, potency and transducer ratio values as follows:


$$\begin{array}{c} E_{max}=R_{0}\frac{\frac{k_{1}^{+}}{k^{-}}+k_{int}\left(\frac{\frac{k_{2}^{+}}{k^{-}}+\frac{k_{3}^{+}}{k^{-}}\frac{k_{r23}}{k_{r31}}}{k_{r21}+k_{r23}}\right)+p_{23}k_{int}\left(\frac{\frac{k_{3}^{+}}{k^{-}}}{k_{r31}}-\frac{\frac{k_{2}^{+}}{k^{-}}+\frac{k_{3}^{+}}{k^{-}}\frac{k_{r23}}{k_{r31}}}{k_{r21}+k_{r23}}\right)}{1+k_{int}\left(\frac{1+\frac{k_{r23}}{k_{r31}}}{k_{r21}+k_{r23}}\right)+p_{23}k_{int}\left(\frac{1}{k_{r31}}-\frac{1+\frac{k_{r23}}{k_{r31}}}{k_{r21}+k_{r23}}\right)}, \end{array}$$ (6)

$$\begin{array}{c} EC_{50}=K_{D}\left(\frac{1+\frac{k_{int}}{k_{off}}}{1+k_{int}\left(\frac{1+\frac{k_{r23}}{k_{r31}}}{k_{r21}+k_{r23}}\right)+p_{23}k_{int}\left(\frac{1}{k_{r31}}-\frac{1+\frac{k_{r23}}{k_{r31}}}{k_{r21}+k_{r23}}\right)}\right), \end{array}$$ (7)

and


$$\begin{array}{c} \tau =\frac{R_{0}\left(\frac{k_{1}^{+}}{k^{-}}+k_{int}\left(\frac{\frac{k_{2}^{+}}{k^{-}}+\frac{k_{3}^{+}}{k^{-}}\frac{k_{r23}}{k_{r31}}}{k_{r21}+k_{r23}}\right)+p_{23}k_{int}\left(\frac{\frac{k_{3}^{+}}{k^{-}}}{k_{r31}}-\frac{\frac{k_{2}^{+}}{k^{-}}+\frac{k_{3}^{+}}{k^{-}}\frac{k_{r23}}{k_{r31}}}{k_{r21}+k_{r23}}\right)\right)}{K_{D}\left(1+\frac{k_{int}}{k_{off}}\right)}. \end{array}$$ (8)

**Fig 3:**
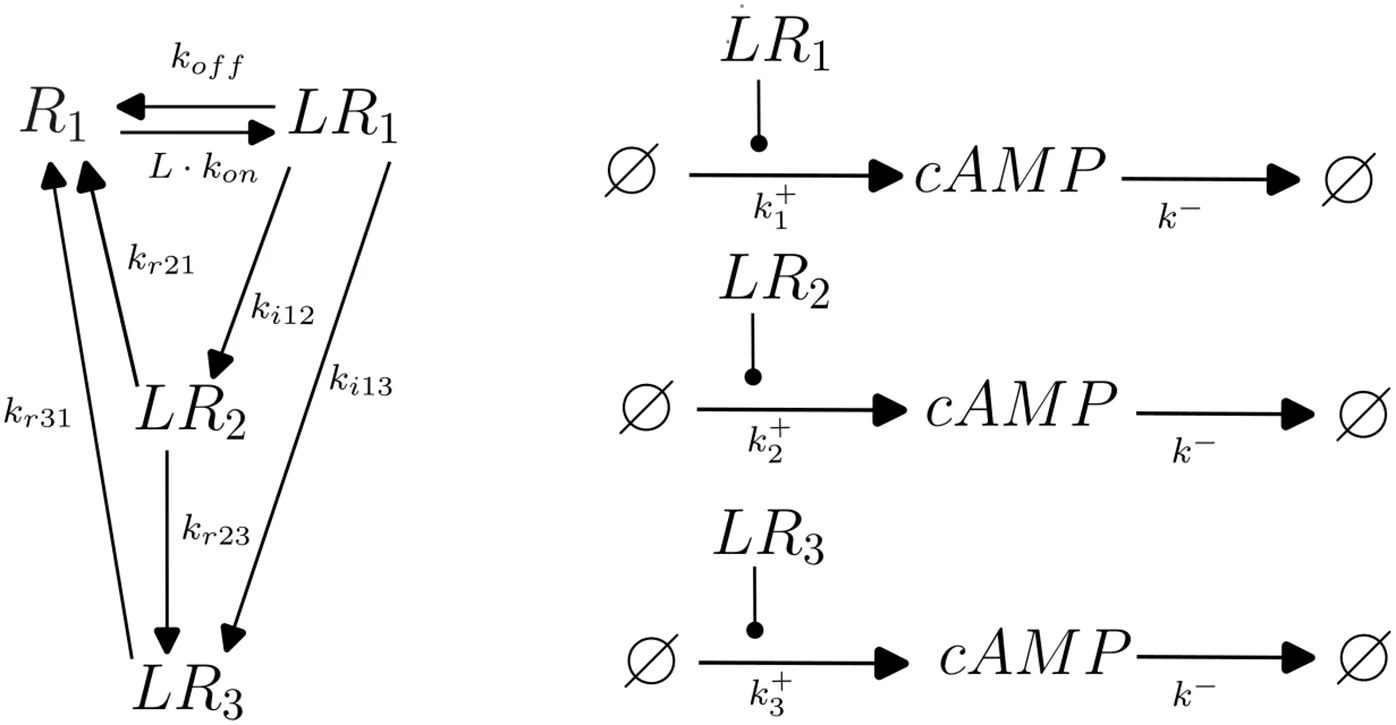
Model 2. The left panel illustrates receptor trafficking, whereas the right panel depicts second messenger production and degradation. *R*_1_ denotes the amount of free receptors at the plasma membrane, *LR*_1_, *LR*_2_ and *LR*_3_ the amount of ligand-receptor complex at the plasma membrane and two types of intracellular compartments. cAMP refers to the quantity of second messenger molecules. A parameter was associated to each reaction rate (represented by arrows).

As in Fig 2, Fig 4 illustrates that promoting ligand-receptor accumulation in the third compartment (*e.g.,* increasing *p*_23_) can exert opposing effects on potency, efficacy, and transducer ratio. This reflects the intricate interplay between receptor trafficking parameters and signaling parameters, as captured by Eqs. (6)-(8). Remarkably, inducing ligand-receptor accumulation in the third compartment (e.g., increasing *p*_23_) always leads to a monotonic relation between $E_{max}$, *EC*_50_ and τ with respect to *p*_23_. However, as shown on Fig 4, all qualitatively distinct cases are theoretically attainable. Characterizing the dynamics of each case could help identify the most plausible scenario based on current knowledge, and provide a framework for dissecting receptor trafficking and compartmentalized signaling. Notably, the compartment maturation rate from *LR*_2_ to *LR*_3_ ($k_{r23}$) has no effect on the variations of the potency, efficacy or transduction coefficient. Potency is affected only by recycling rates, consistent with the result obtained for the first model. The interpretation of the first case (Fig 4A) is as follows: when the recycling rate is faster from *LR*_2_ than from *LR*_3_ ($k_{r21}>k_{r31}$), and cAMP production in the third compartment is high enough (Eq. (19)), then the accumulation of receptor in the third compartment leads to globally more efficient response ($E_{max}$ increases, *EC*_50_ decreases, τ increases). The fourth case (Fig 4D) is symmetrically opposite to the first case, with a globally less efficient response.

**Fig 4:**
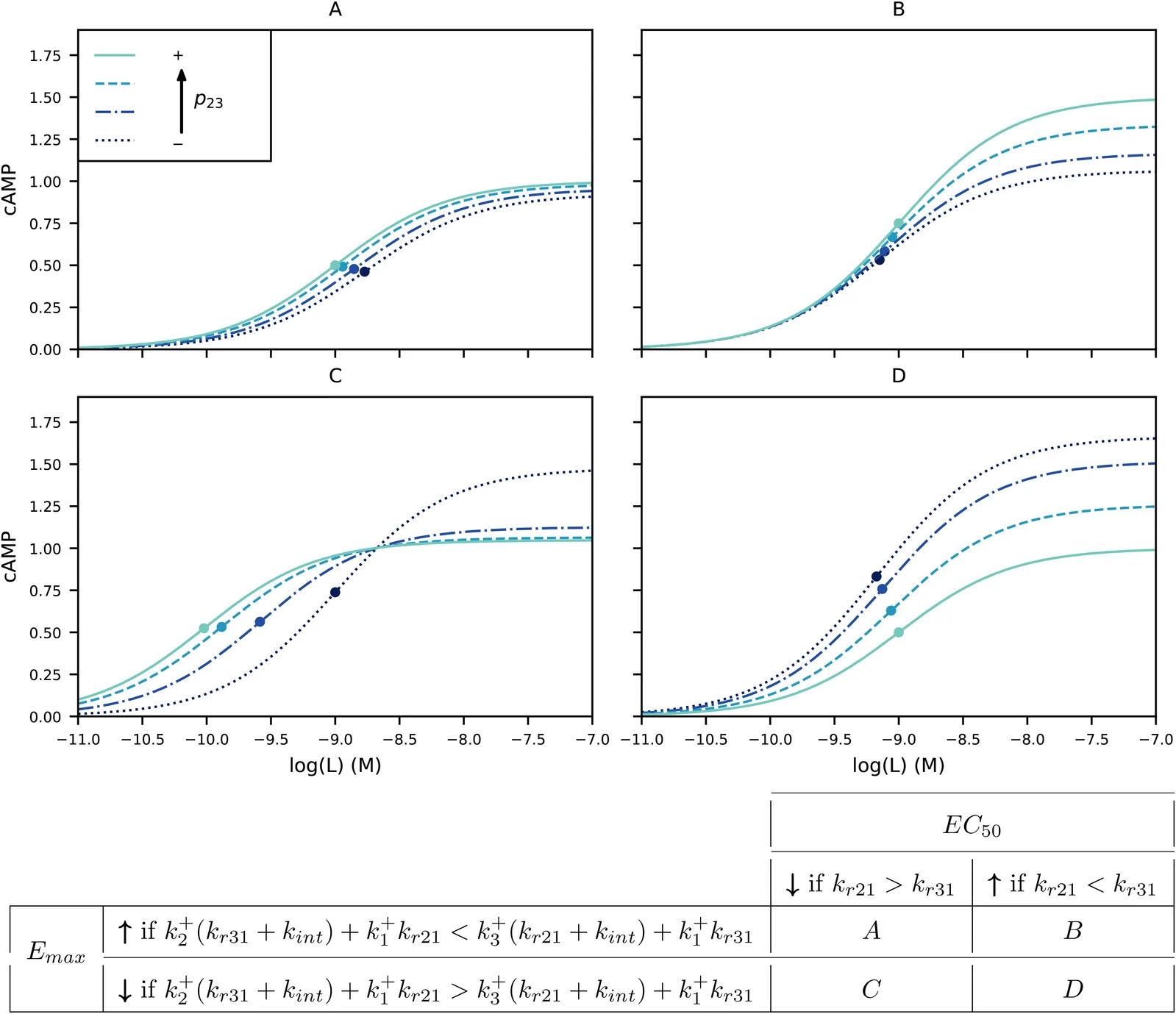
Impact of biasing receptor distribution on cAMP production using dose-response analysis. In the different panels, the ligand (M) dose response relationship of model 2 (Fig 3) is plotted for different values of *p*_23_. The values of *p*_23_ represent the increasing of receptors in the third compartment. Common parameters are *p*_23_ = 0 (dotted line), *p*_23_ = 0.3 (dashdot line), *p*_23_ = 0.7 (dashed line) and *p*_23_ = 1 (solid line),; $K_{D}=10^{-9}$, *R*_0_ = 1, *k*^–^ = 1, $k_{3}^{+}=1$, $k_{int}=1$, $k_{r23}=1$, $k_{off}=1$, whereas the recycling ($k_{r21}$ and $k_{r31}$) and production ($k_{1}^{+}$ and $k_{2}^{+}$) parameters are specific to each panel: **(A)** $k_{r21}=10$, $k_{r31}=1$, $k_{1}^{+}=1$, $k_{2}^{+}=0.01$; **(B)** $k_{r21}=0.01$, $k_{r31}=1$, $k_{1}^{+}=1$, $k_{2}^{+}=0.01$; **(C)** $k_{r21}=20$, $k_{r31}=0.05$, $k_{1}^{+}=2$, $k_{2}^{+}=0.01$; **(D)** $k_{r21}=0.01$, $k_{r31}=10$, $k_{1}^{+}=1$, $k_{2}^{+}=2$. Circles represent the value of *EC*_50_. Parameter values were arbitrarily chosen, for illustration purposes.

In contrast, the second (Fig 4B) and third cases (Fig 4C) are more subtle. In those later cases, the impact of receptor accumulation in the third compartment is dose-dependent, and the overall effect can be quantified by the transduction coefficient τ, whose variation depends on the relation between signaling efficacy and receptor recycling (Eq. (20)).

In conclusion, unlike the receptor internalization inhibition case studied in the first subsection, a change in $E_{max}$ induced by biasing receptor spatial distribution is harder to interpret in terms of parameters values and, therefore, receptor trafficking or cAMP production. However, a shift in the dose-response curve (*EC*_50_) is indicative of differential recycling rates from distinct intracellular compartments, and the transduction coefficient τ may further provide insight into the signaling efficacy within each compartment.

### Evidence that FSHR signals through two distinct intracellular compartments

As demonstrated by the results presented in the previous section, receptor trafficking and localized signaling parameters may substantially influence cellular responses. Our next objective was therefore to show that we can identify the parameter regime governing a given signaling pathway using dedicated biological experiments and parameter estimation framework. We used the FSHR-induced cAMP signaling pathway as a case study. As introduced earlier, FSHR undergoes an atypical receptor trafficking sequence, being internalized predominantly into VEEs rather than EEs, while the transitions between these two compartments remain unknown to date [6,8]. While the distinct functional roles of these two compartments remain to be fully elucidated, their sequential nature and the spatially distinct signaling they may support justify treating them as separate compartments in the model.

#### Kinetic experiments and model can jointly reveal receptor trafficking and compartmentalized signaling parameters.

Using Bioluminescence Resonance Energy Transfer (BRET) measurements, we monitored the temporal dynamics of molecular species following FSH stimulation in a cell system overexpressing FSHR (Fig A in S1 Text) [30]. We considered four different BRET experiments. First, we quantified total cAMP levels using an intramolecular biosensor (EPAC) upon continuous stimulation with varying FSH concentrations (FSH = 0, 0.1, 0.3, 1, 3, 10 nM). At a single FSH concentration (1 nM), cAMP level was monitored in control conditions or in the presence of PitStop2, a chemical inhibitor of clathrin-mediated endocytosis (CME), hereafter referred to as *ps*_2_. Localization of receptor in specific cellular compartments was assessed by monitoring interaction between FSHR fused to the luciferase RLuc8 (FSHR-RLuc8) and a fluorescent protein Ypet localized either at plasma membrane (Lyn-Ypet) or at early endosomes (Ypet-FYVE) (Fig B in S1 Text). Measuring the interaction between Lyn-Ypet and FSHR-RLuc8 enables to monitor FSHR localization at the plasma membrane, which decrease upon ligand-induced internalization. In contrast, monitoring interaction between FSHR-RLuc8 and Ypet-FYVE allowed to quantify the time-dependent accumulation of ligand-receptors complexes in early endosomes. Receptor internalization (PM_R_) and trafficking to the EEs (EE_R_) kinetics were assessed in basal conditions (FSH = 0) and with FSH = 100 nM.

Model 2 (Fig 3) represents the atypical trafficking *a priori* known for FSHR [6,8] in which *LR*_1_, *LR*_2_ and *LR*_3_ represent ligand-receptor complexes at the plasma membrane (PM), in the very early endosomes (VEE) or early endosomes (EE). As cAMP data show moderate but persistent signal decreases at high doses (Fig A in S1 Text), we further incorporated the possibility of irreversible receptor desensitization from *LR*_1_, at the plasma membrane, and from *LR*_3_ at the early endosomes to obtain the more general model 3 (Fig 5). This complete model yielded an ODE system detailed in Methods -Eq. (21). We assumed the BRET signal is proportional to the quantity of measured species in the cell [31]. These signals were then linked to species evolution according to the following observable equations:


$$\begin{array}{c} \left\{ \begin{array}{c} cAMP=\alpha_{1}\left(cAMP_{1}+cAMP_{2}+cAMP_{3}\right),\\ cAMP^{ps_{2}}=\alpha_{1}\left(cAMP_{1}^{ps_{2}}+cAMP_{2}^{ps_{2}}+cAMP_{3}^{ps_{2}}\right),\\ PM_{R}=\alpha_{2}\left(R_{1}+LR_{1}-R_{0}\right),\\ EE_{R}=\alpha_{3}LR_{3}\;. \end{array}\right. \end{array}$$ (9)

**Fig 5:**
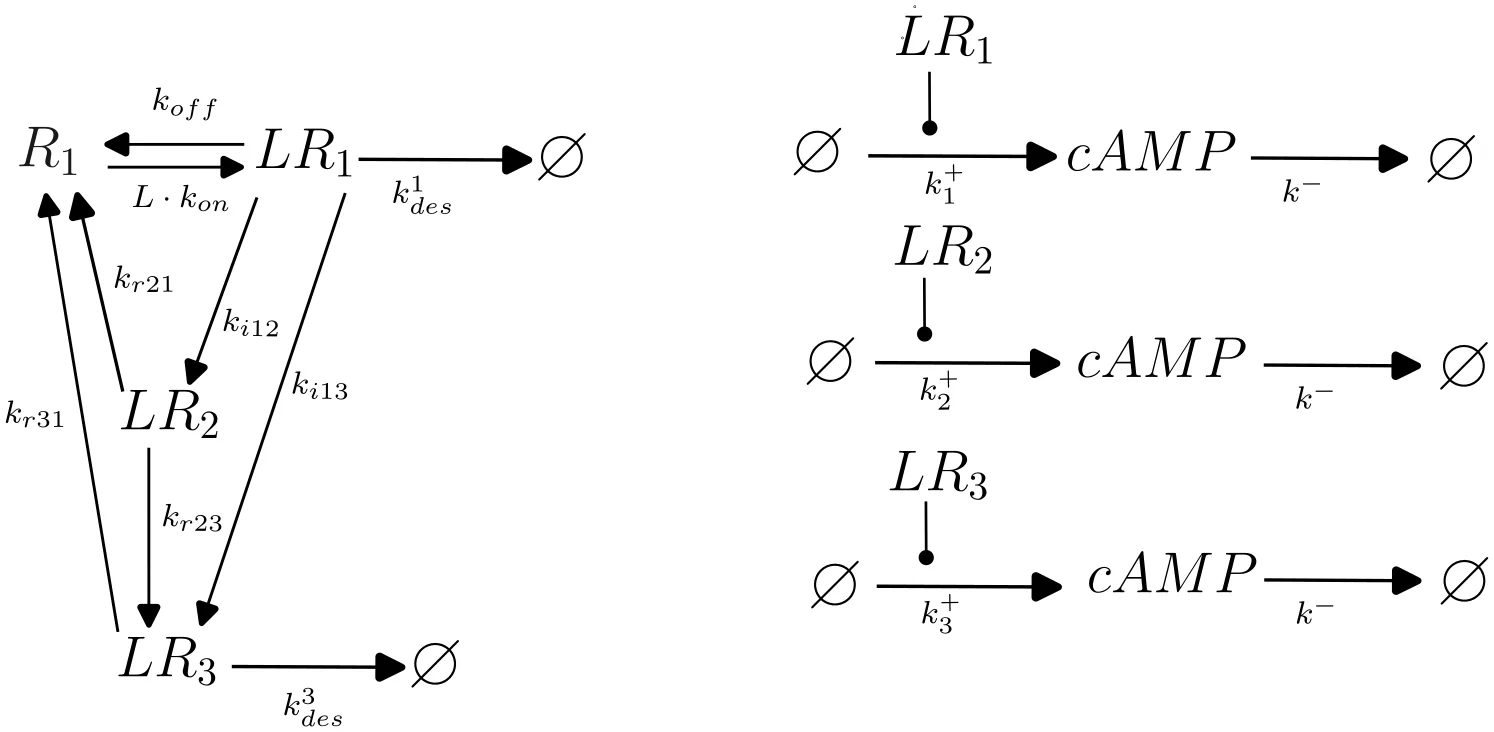
Model 3. The left panel illustrates receptor trafficking, whereas the right panel depicts second messenger production and degradation. *R*_1_ denotes the amount of free receptors at the plasma membrane, *LR*_1_, *LR*_2_ and *LR*_3_ the amount of ligand-receptor complex at the plasma membrane (PM) and two types of intracellular compartments. cAMP refers to the quantity of second messenger molecules. A parameter was associated to each reaction rate (represented by arrows).

Importantly, prior to parameter estimation, it was essential to verify that the experimental design and model structure yielded a well-posed - i.e., identifiable - inverse problem. To this end, we first reparametrized the ODE system, together with the observable equations, and non-dimensionalized the parameters, so as to reduce the total number of parameters as much as possible. The resulting normalized model led to Eq. (22), comprising 19 unknown parameters instead of 21 (Tables 3 and 4) in which new parameters $\lambda_{tot}$, $\lambda_{1}$ and $\lambda_{3}$ represent the cellular response produced by receptors (Eqs. (23)). Applying the methodology of [32], we demonstrated that the parameter estimation problem is locally identifiable given the observables (Eq. (9)) meaning that each set of observable outputs locally determines a single set of parameter values. Interestingly, when the internalization inhibition data with PitStop2 were excluded, the model became non identifiable for a subset of parameters, including several trafficking and cellular response parameters ($\lambda_{tot}$, $k_{i13}$, $k_{r21}$, $k_{r31}$, $k_{r21}$, $k_{des}^{1}$, $k_{des}^{3}$, $\lambda_{1}$ and $\lambda_{3}$). This result demonstrates the value of the experimental design in jointly constraining - and thereby disentangling - receptor trafficking and compartmentalized signaling parameters.

#### Identifying a cluster of models.

We developed a model selection procedure aimed at identifying parsimonious models with fewer parameters than the complete model 3 (Fig 5), in order to discriminate among the following alternative questions, given the available data (Fig 6):

Q1. Does the ligand-receptor complex internalize in one or two compartments (EE or VEE/EE) i.e., is $k_{i12}=0$?
Q2. Is the ligand-receptor complex functionally active in two compartments (PM and VEE, i.e., is $k_{3}^{+}=0$ or PM and EE, i.e., is $k_{2}^{+}=0$) or three compartments (PM, VEE and EE)?
Q3. Is chemical internalization inhibition partially or fully efficient, i.e., is *ps*_2_ = 0?
Q4. Are there irreversible receptor desensitization mechanisms at the plasma membrane, i.e., is $k_{des}^{1}=0$?
Q5. Are there irreversible receptor desensitization mechanisms at early endosomes, i.e., is $k_{des}^{3}=0$?
Q6. If there are three active signaling compartments, what is the trafficking pathway that the ligand-receptor complex may follow (internalization, traffic to an other compartment, recycling)– i.e., what is the most plausible trafficking model among 8 competitive models (Fig C in S1 Text)?

**Fig 6:**
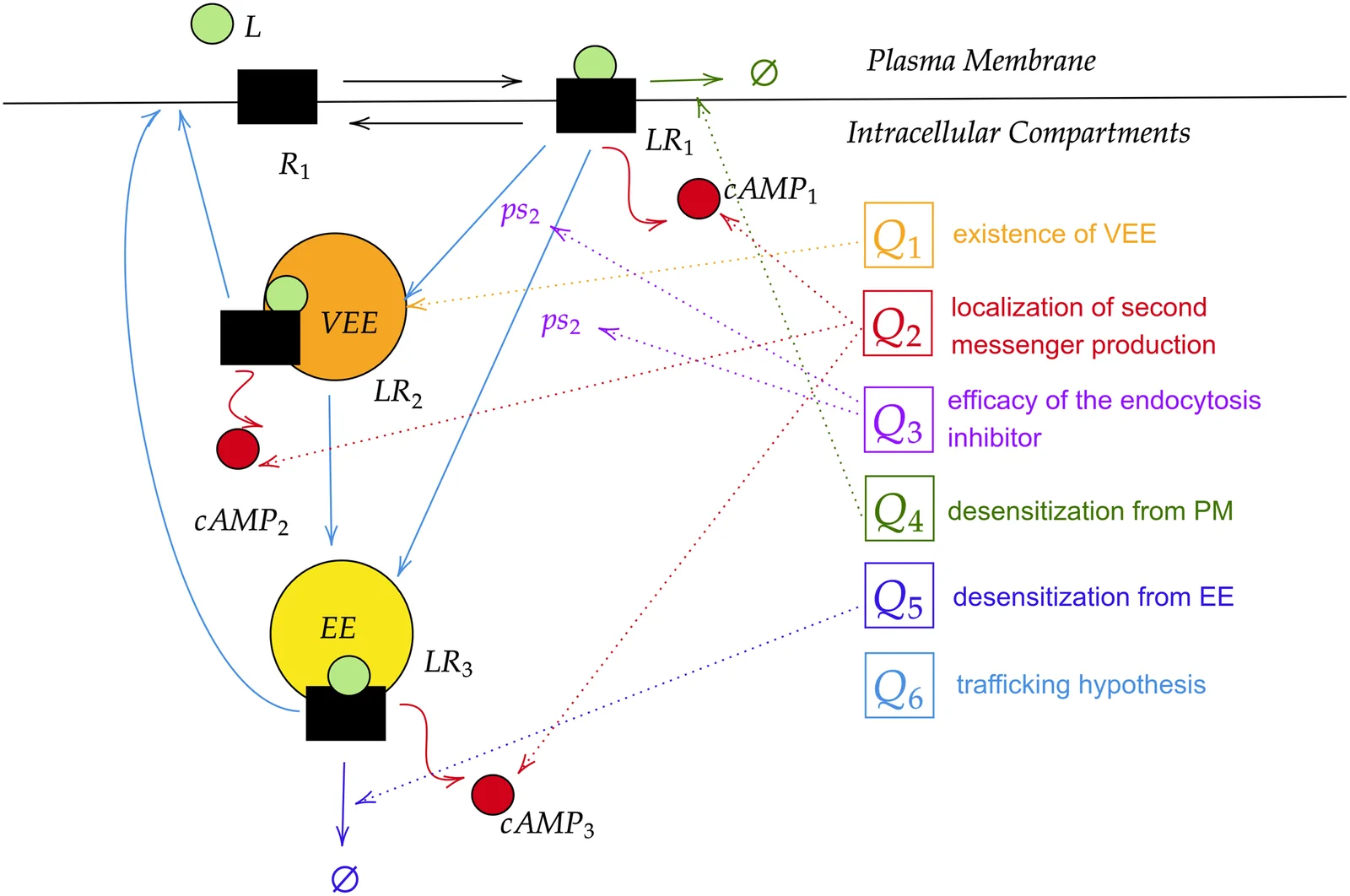
Schematic model diagram highlighting the different questions (Q1-Q6) tested during the model selection. The scheme was done on MatchaIO.

Those six questions were associated to alternative model formulation that led to 200 submodels of the complete model 3 (Fig 5) (Table B in S1 Text). We ran the model selection algorithm on the 200 models and we ranked them according to the AIC criterion (Table B in S1 Text). Rather than a single best model, 5 models have a $\Delta_{AIC}<2$ and are therefore considered as likely as the best model; moreover, up to 14 models have $2<\Delta_{AIC}<7$, and can thus be regarded as suitable alternatives. Importantly, all 19 best models share common features. Analysis below considers only the best 5 models, yet results extend to models from 3.6 to 3.19 (Table E in S1 Text). First, three compartments are required (Q1), with ligand-receptor complexes being functionally active within each compartment individually (Q2). Model selection rejects the scenario in which production arises solely from PM and VEE (*LR*_1_ and *LR*_2_) or from PM and EE (*LR*_1_ and *LR*_3_), even though biological model suggests that active receptors may be abundant in VEE (*LR*_2_). The data are compatible with the hypothesis that the CME inhibitor is fully effective (Q3) (model 3.5 is the only model with a positive parameter *ps*_2_, but it has very low value). The data also support the existence of an irreversible receptor desensitization mechanism at the plasma membrane (Q4), while we cannot conclude about the desensitization mechanism from early endosomes (Q5). Finally, we cannot currently identify a unique best trafficking model (Q6) (Table C in S1 Text), as three trafficking hypotheses (c, b and i) fall below the threshold $\Delta_{AIC}<2$ (Table 1). These three trafficking models all exclude the internalization route from PM to EE ($k_{i13}=0$), but share the assumption that VEEs can mature into EEs ($k_{r23}>0$). In contrast, all models that omit the transition from VEE to EE (trafficking model g and h) have $\Delta_{AIC}\approx 16$ (Table B in S1 Text) and were thus clearly rejected.

**Table 1:** Summary of results for the five best models selected based on the AIC criterion. The table below reports the outcomes of the selection procedure for models 3.1, 3.2, 3.3, 3.4 and 3.5, including the answers to 0–0 and the corresponding statistics for each model (negative log-likelihood, AIC, $\Delta_{AIC}$, number of estimated parameters, convergence status), revealing the existence of two distinct model clusters. Convergence was calculated as the number of runs with a negative log-likelihood value below the minimum negative log-likelihood value plus 0.1.

	3.1	3.2	3.3	3.4	3.5
Q1 - Existence of VEE	yes	yes	yes	yes	yes
Q2 - Localization of second messenger production	*LR*_1,2,3_	*LR*_1,2,3_	*LR*_1,2,3_	*LR*_1,2,3_	*LR*_1,2,3_
Q3 - Efficacy of the inhibitor endocytosis	total	total	total	total	partial
Q4 - Desensitization from PM	yes	yes	yes	yes	yes
Q5 - Desensitization from EE	no	no	yes	yes	no
Q6 - Trafficking hypotheses	c	b	i	c	c
Internalization from PM to EE	no	no	no	no	no
Recycling from VEE	no	yes	yes	no	no
Internalization from PM to VEE	yes	yes	yes	yes	yes
Recycling from EE	yes	yes	no	yes	yes
Negative log-likelihood	-8329.94	-8329.96	-8329.96	-8329.94	-8329.94
AIC	-16629.88	-16627.91	-16627.91	-16627.88	-16627.88
$\Delta_{AIC}$	0.00	1.97	1.97	2.00	2.00
Number of estimated parameters	15	16	16	16	16
Convergence (%)	77.8	83.6	78.9	68.0	75.0
Cluster	1	2	2	1	1

The parameter estimation procedure yielded estimates for all parameters of each model (Table C in S1 Text), which were subsequently used for all predictions. We verified that the five best models yield comparable fits, as illustrated in Fig 7, which demonstrates strong agreement between model predictions and experimental data across all observables - cAMP production, receptor internalization, and receptor trafficking to early endosomes - both under control conditions and upon CME inhibition. Verifying practical parameter identifiability for the different models is essential to perform reliable model prediction. Hence, to further investigate the five best models (Table B in S1 Text, models 3.1-3.5), we performed a profile likelihood estimate for each parameter on each model (Fig 8). Some parameters are clearly identifiable with very small confidence intervals and common values across the eight models ($k_{off}$, $K_{D}$, $k^{-*}$, $\sigma_{1}$, $\sigma_{2}$, $\sigma_{3}$, $\alpha_{2}^{*}$, $k_{des}^{1*}$), whereas some are consistent across models but have larger confidence intervals ($k_{i12}^{*}$, $k_{r21}^{*}$, $\alpha_{3}^{*}$, $\lambda_{1}$, $\lambda_{tot}$). The remaining parameters are consistent across some models but exhibit large confidence intervals in others ($k_{r23}^{*}$, $\lambda_{3}$, $k_{r31}^{*}$, $k_{des}^{3*}$) (Fig 8).

**Fig 7:**
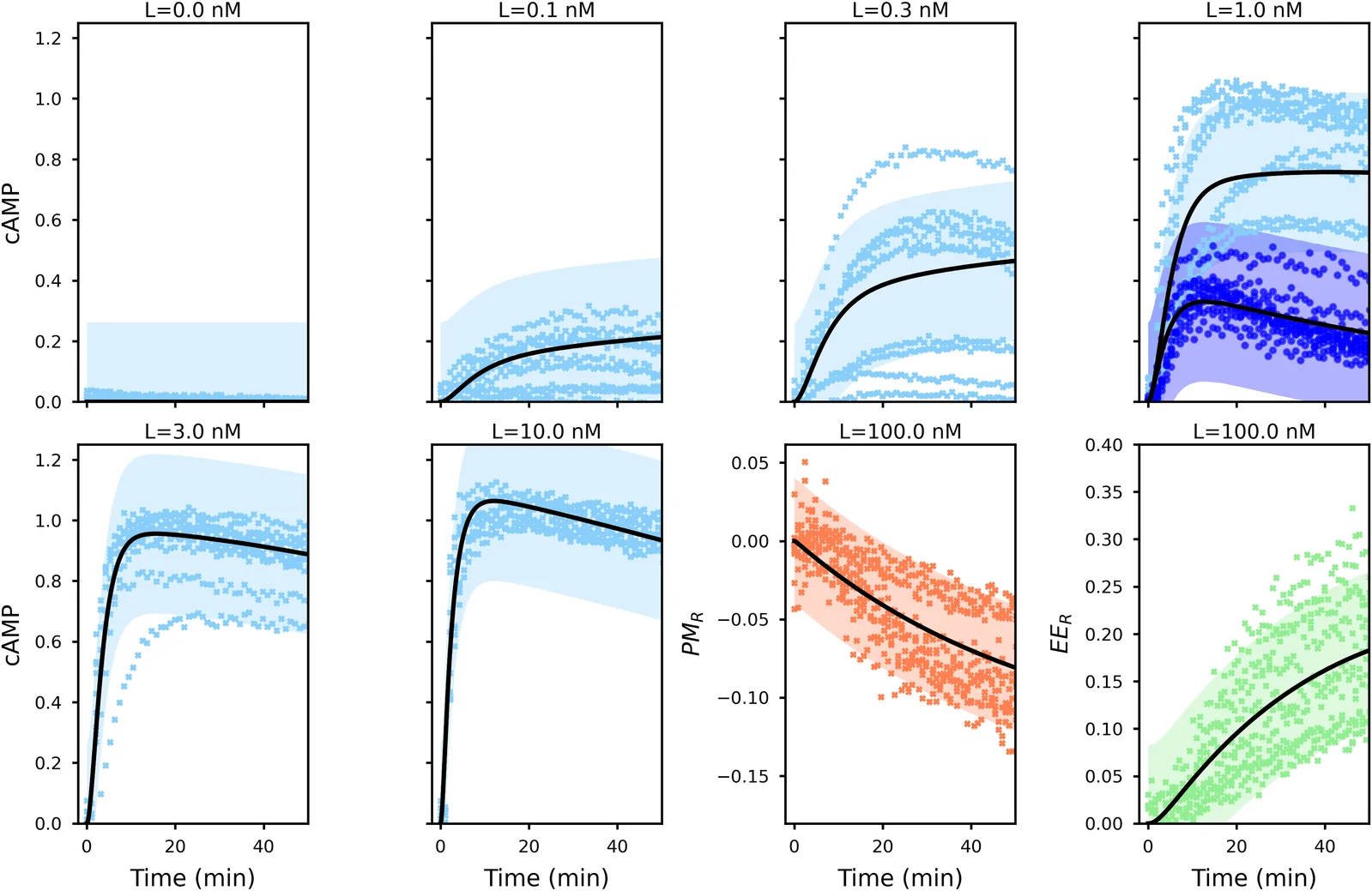
Model fitting for the five best models. The five best fit of the ODE simulation (Table B in S1 Text, models 3.1-5) are superimposed while shading area indicates statistical uncertainty for the first model. Experimental data points are shown as crosses. For the 1 nM ligand condition, the bottom curve (shown in dark blue) corresponds to cAMP levels measured under PitStop2 treatment.

**Fig 8:**
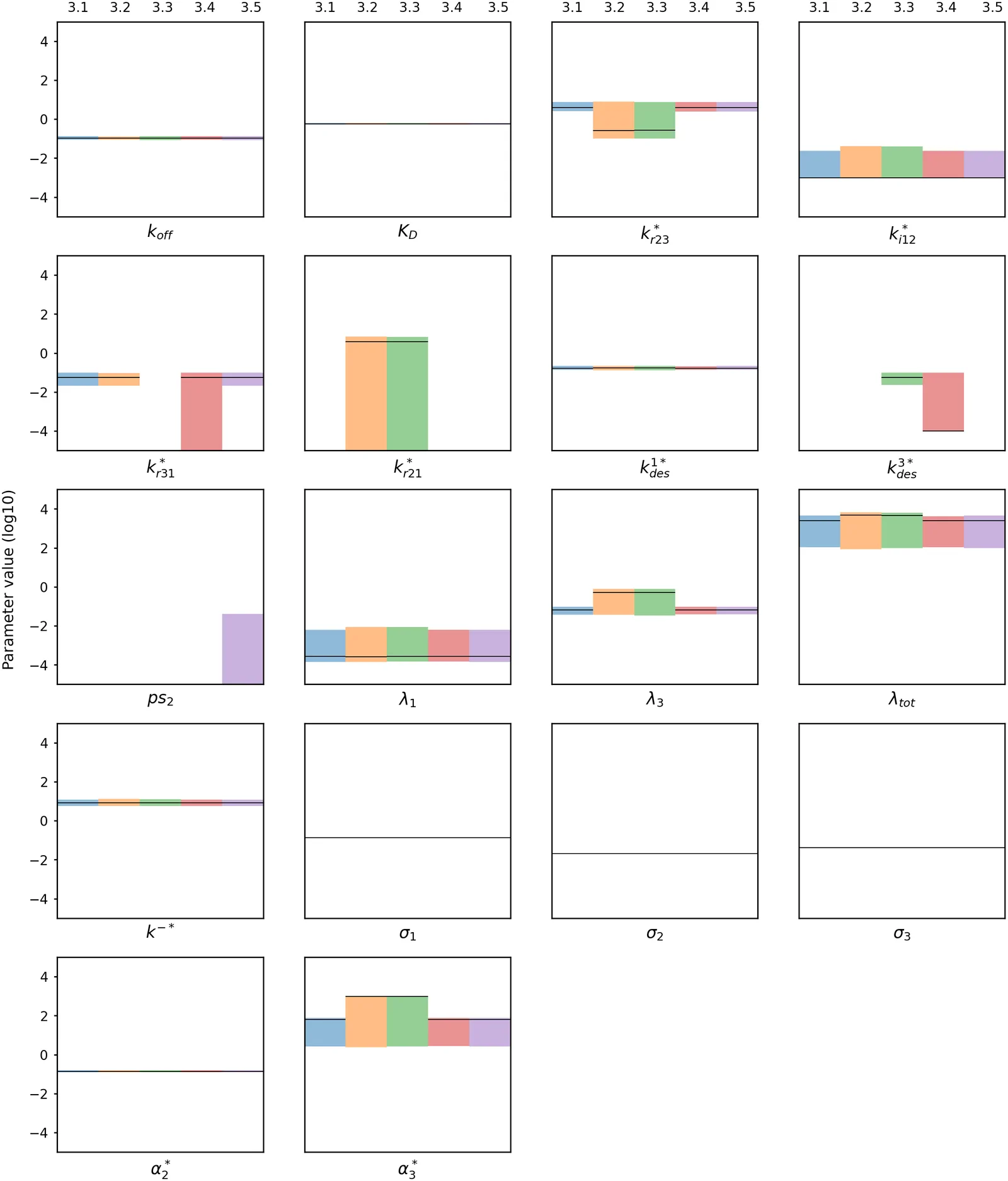
Confidence intervals for all parameters in the five best models. In each panel, we show the *profile-based* 95% confidence interval of the normalized parameter for the 5 selected best models (Table B in S1 Text). The optimum value is represented by the dark line.

Parameters governing time scales, ligand-receptor interactions, and observables are well identified, while those related to receptor trafficking and localized cAMP production remain subject to greater uncertainty. Models 3.1, 3.4 and 3.5 share the common trafficking hypothesis *c* and lead to similar parameter values and practical identifiability (except for $k_{r31}^{*}$). Similarly, models 3.2 and 3.3 also share similar parameter values and practical identifiability, but distinct from models 3.1, 3.4 and 3.5. This distinction is particularly evident for several parameters, including $k_{r23}^{*}$, $\lambda_{3}$ and $\alpha_{3}^{*}$ (Fig 8). Thus, within the five best models, two groups emerge (cluster 1 and cluster 2 -Table 1) that are compared in the subsequent study.

#### Uncertainties on internalized receptors do not affect predictions on localized cAMP production.

Despite our theoretical identifiability results, parameter uncertainty may arise from the inherent variability of the experimental data and/or from insufficient number of observables or experimental conditions. As apparent from Fig 7, experimental variability is high, which necessarily increases the variance of the statistical error model. In Fig 9, we plot the *profile-based* 95% confidence interval (shaded area), revealing that parameter uncertainty does not affect cAMP level predictions for model 3.1 (first column), but significantly impacts the predicted amount of internalized receptors. Similarly, Fig 9 (second column) shows that model predictions for each cAMP variable and for the amount of receptors at PM and in VEE (*LR*_1_ and *LR*_2_) are consistent across the five best models. However, models differ substantially in their predictions of the amount of internalized receptor in the EE (*LR*_3_). The prediction uncertainty in internalized receptor likely reflects variability in trafficking and localized production parameters, which may partially compensate for one another to reproduce a given cAMP output-at least within our dataset. A negative correlation can indeed be observed between the number of ligand-receptor complexes in EE (*LR*_3_) and the cAMP production rate in this compartment ($k_{3}^{+}$). Indeed, the amount of *LR*_3_ is higher for models with trafficking hypotheses *c* (cluster 1, models 3.1, 3.4 and 3.5), while cAMP production in the third compartment is lower in these models (Fig 9 and Table C in S1 Text). In comparison, models 3.2 and 3.3 (cluster 2) have less internalized receptors and a highest cAMP production in the third compartment. A similar trend is observed within the 19 best models that are all consistent for cAMP dynamics but differs in internalized receptors (Fig H in S1 Text), possibly highlighting a third cluster (Table E in S1 Text). It is to be noted that the best models do not differ in their amount of internalized receptors (less than 1%, see next subsection) but differs in their relative proportion of *LR*_2_ and *LR*_3_ (Table 2).

**Table 2:** Averaged outputs for the five best models. The table presents the proportion of internalized receptors ($LR_{intra}=LR_{2}+LR_{3}$) relative to total receptors, and the proportion of receptors in EE (*LR*_2_) and VEE (*LR*_3_) relative to internalized receptors, as well as endocytosis and recycling time and probability for a 5min incubation with 10nM of ligand. Values in brackets indicate uncertainty.

	3.1	3.2	3.3	3.4	3.5
$LR_{intra}$ (%)	0.05	0.02	0.02	0.05	0.05
	[0.04, 1.14]	[0.01, 1.24]	[0.01, 1.12]	[0.04, 1.09]	[0.04, 1.12]
*LR*_2_ (%)	43.95	92.32	92.17	43.96	43.95
	[27.16, 52.28]	[27.41, 97.20]	[25.68, 97.19]	[28.50, 52.68]	[27.33, 53.21]
*LR*_3_ (%)	56.05	7.68	7.83	56.04	56.05
	[47.72, 72.84]	[2.80, 72.59]	[2.81, 74.32]	[47.32, 71.50]	[46.79, 72.67]
Time endocytosis (s)	58.62	58.42	58.41	58.62	58.62
	[49.76,70.26]	[48.77,69.48]	[49.25,69.67]	[49.58,69.97]	[49.74,70.39]
Probability endocytosis	0.01	0.01	0.01	0.01	0.01
	[0.00,0.14]	[0.00,0.19]	[0.00,0.18]	[0.00,0.13]	[0.00,0.14]
Time recycling (s)	168.88	13.15	2.33	168.88	168.88
	[105.65,434.49]	[4.79,191.71]	[1.20,2.71]	[107.65,234.69]	[105.65,436.66]
Probability recycling	1.00	1.00	0.93	1.00	1.00
	[1.00,1.00]	[1.00,1.00]	[0.00,0.98]	[0.00,1.00]	[1.00,1.00]

**Fig 9:**
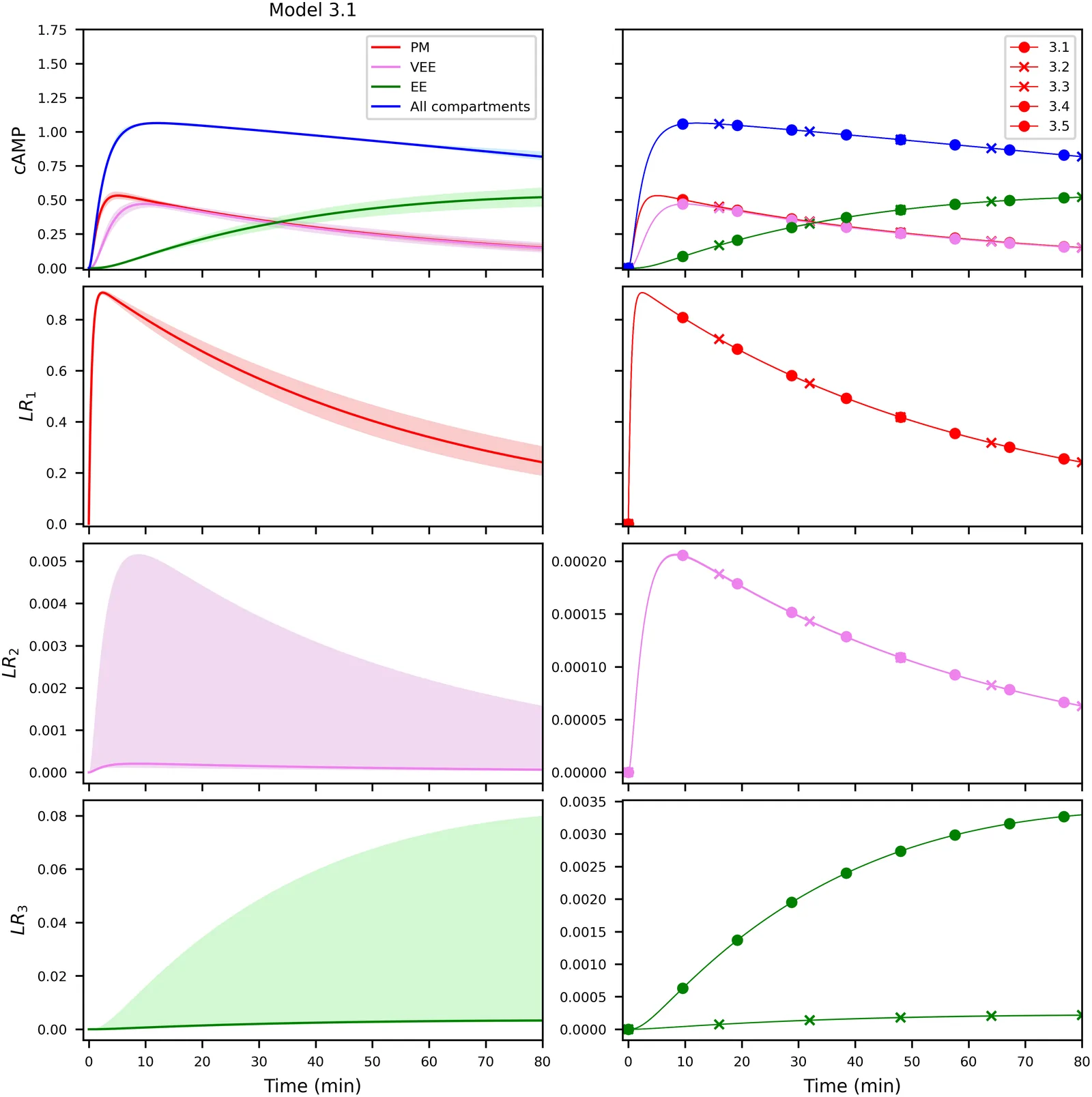
Prediction and uncertainty quantification of cAMP levels and LR complexes in each compartment for the five best models. Model 3.1 predictions are shown with 95% profile-based confidence intervals (left column), alongside single predictions for all best models (right column) in response to a single FSH dose (10 nM). Rounds show cluster 1 and crosses the cluster 2.

To further discriminate among the best models, an interesting perspective would be to design experiments using sensors selectively localized in distinct endosomal compartments to monitor the quantities of *LR*_2_ and *LR*_3_ [6]. Such spatially resolved data would help discriminate between models and strengthen our conclusions regarding hypotheses Q1 to Q5. However, designing such selective sensors is currently challenging, as VEEs lack specific molecular markers. To illustrate this, Fig D in S1 Text shows model predictions for models 3.33, 3.60, 3.40, and 3.132, which all share the common *c* trafficking structure as their best model (Fig C in S1 Text), but differ in their assumptions regarding hypotheses: desensitization from EE (3.33) (Q5), no desensitization from PM (3.60) (Q4) and cAMP production at PM and VEE (3.40) or at PM and EE (3.132) (Q2), respectively (Table B in S1 Text). These alternative models deviate only marginally from the best model in terms of total cAMP - failing in particular to capture the long-time cAMP decrease - but diverge more substantially in their predictions of internalized receptor amounts and endosomal cAMP levels (Table D in S1 Text).

#### FSHR activation leads to a strong endosomal signaling response.

The five best models exhibit very few internalized receptors (less than 1%, Table 2) that yet elicit a strong cellular response ($k_{2}^{+}$ and $k_{3}^{+}$ are three to four order of magnitude higher than $k_{1}^{+}$, Table C in S1 Text). The first and transient wave of cellular response originates from signaling at the plasma membrane and in very early endosomes (*LR*_2_), with a slightly higher response in VEE. A second and sustained wave of signaling corresponds to cAMP production in EEs (*LR*_3_) (Fig 9). Further, receptor internalization rates (of order $10^{-4}-10^{-5}s^{-1}$) appear to be considerably slower than receptor recycling rates (of order $10^{-2}-10^{0}s^{-1}$), the latter being comparable to ligand unbinding rate (of order $10^{-1}s^{-1}$). All models predict an averaged endocytosis time of 58*s* with a rather sharp confidence interval, while the averaged receptor recycling time has much more uncertainty across models, ranging from 2 to 169*s*. Interestingly, for models with receptor recycling from VEE, the VEE recycling rate is much higher than the EE recycling rate (Table C in S1 Text), in agreement with previously reported dynamics for the LHR [6,8]. Finally, irreversible receptor desensitization occurs mostly at the plasma membrane (with a probability of 0.01 for receptors to be internalized in endosomes) while most receptors within the endosomes are recycled back to the plasma membrane. Results remain consistent across models 3.6 to 3.19 (Table F in S1 Text).

Given relatively comparable receptor recycling and ligand unbinding rates, but markedly contrasted signaling rates between the plasma membrane and endosomal compartments, we expect - in light of the Figs 2 and 4 - that internalization inhibition will primarily reduce signaling efficacy. Simulating dose-response experiments with decreasing internalization rates using our best models (3.1 and 3.2, representing each cluster), we show in Fig 10 that the model indeed predicts that internalization inhibition drastically reduces signaling efficacy, with no apparent effect on potency. Similarly, biasing the spatial distribution of receptors - by inducing ligand-receptor accumulation in the third compartment - has virtually no effect on potency but substantially impacts efficacy. However, the large parameter uncertainty and the lack of agreement between the two best models complicate interpretation: for model 3.1, efficacy decreases as the proportion of *LR*_3_ increases, whereas in model 3.2, efficacy increases as the proportion of *LR*_3_ increases. These contrasting predictions highlight that experimental approaches capable of biasing receptor spatial distribution will be extremely valuable, both for discriminating between models and for reducing parameter uncertainties within a given model.

**Fig 10:**
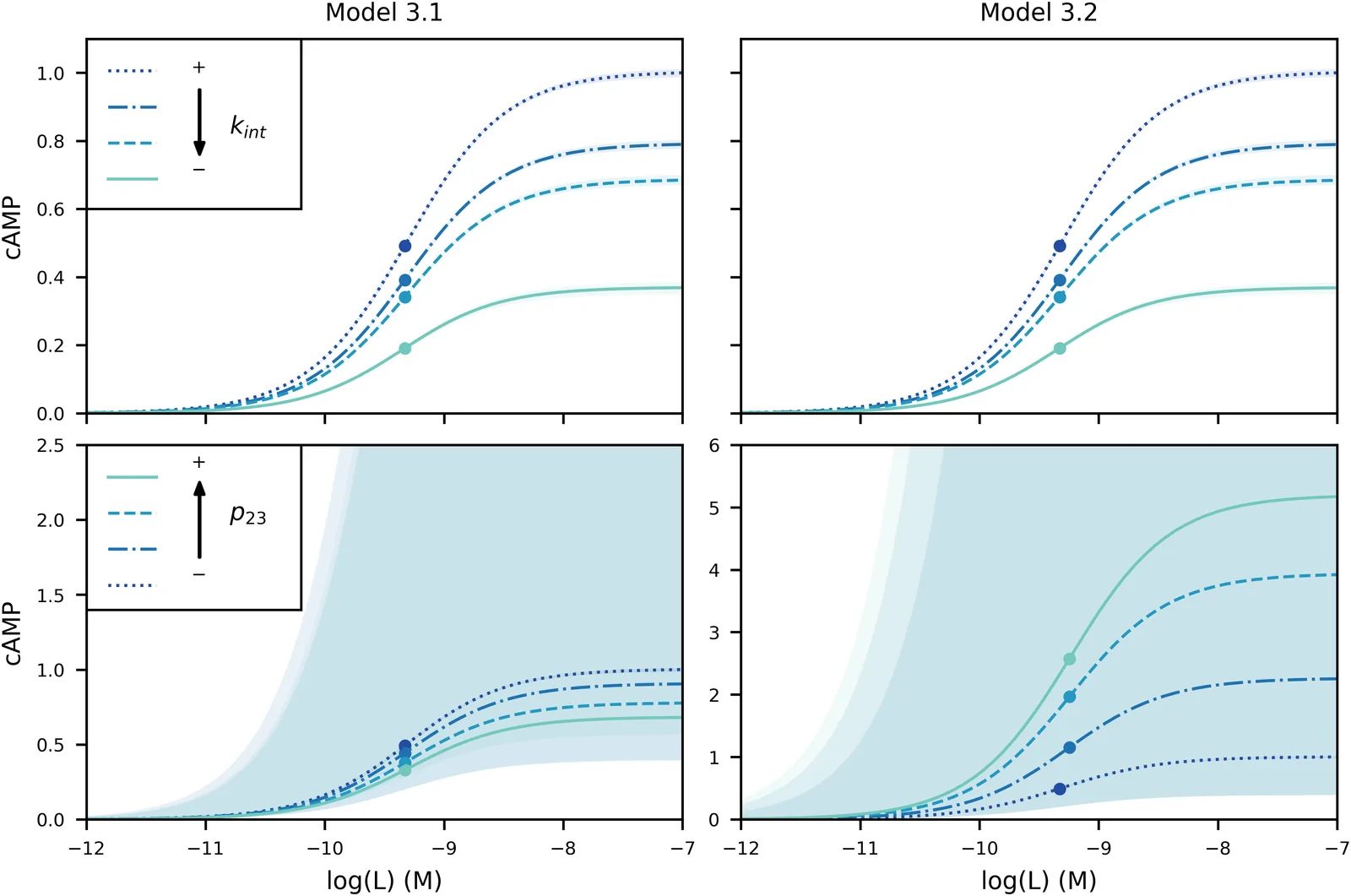
Dose response prediction of the total quantity of cAMP with internalization inhibition and with biased receptor spatial distribution for models 3.1 and 3.2 with 95% profile-based confidence intervals. The different values of internalization are obtained by varying the parameters *ps*_2_: *ps*_2_ = 1, (dotted line), $ps_{2}=\frac{2}{3}$ (dash-dot line), $ps_{2}=\frac{1}{2}$ (dashed line), no internalization: *ps*_2_ = 0 (solid line). The different values for *p*_23_ are 0 (dotted line), 0.3 (dash-dot line), 0.7 (dashed line) and 1 (solid line), while the internalization rate remains constant for each model ($k_{int}=k_{i13}+k_{i12}$, $k_{i13}=p_{23}k_{int}$ and $k_{i12}=(1-p_{23})k_{int}$). Circles represent the value of *EC*_50_. The remaining parameters used for the prediction correspond to the best estimates obtained during model selection (Table C in S1 Text).

#### The model accurately predicts pulse-chase ligand stimulation data.

To strengthen our model predictions, we performed additional experiments for model validation, using both continuous and pulse-chase ligand stimulation. In the later design, the ligand is washed out after one to several minutes of exposure (Fig A in S1 Text-validation data). Pulse-chase experiments are particularly well suited for dissecting the molecular events that occur after ligand binding to its receptor at the plasma membrane, while preventing new ligand binding. Validation data include an additional ligand concentration (30 nM), that was not included in the model fitting data. Three BRET assays were used to monitor total cAMP levels, receptor internalization, and ligand-receptor complexes located in early endosomes (EE). In receptor internalization assays, the CAAX sequence was used instead of LYN to adress the Ypet at PM, but the assay similarly monitored the observable PM_R_.

Fig 11 shows the predictive performance of these models in representing pulse-chase data for a single ligand concentration, across all observables (cAMP, PM_R_, and EE_R_) (Fig E in S1 Text for all ligand concentrations). Nearly all validation data points (≈92% for all the concentrations of ligand) fall within the 95% confidence interval of the statistical error model. Although a systematic model bias is apparent, this may be attributable to variability between independent experiments, as observed in Fig 7. Some differences between cAMP dynamics have to be noticed. Firstly, a notable difference in the timing of cAMP decrease can be observed between conditions. In both cases, cAMP production decreases faster than suggested by the validation data points. One possible explanation is that our model relies on a minimal representation of the cAMP signaling cascade, whereas it is well established that additional molecular actors are involved, as well as non mass-action kinetics phenomena (e.g., phase-separation [33]). Furthermore, for the two highest ligand concentrations (*L* = 10 nM and *L* = 30 nM), model predictions exceed the validation data points during the first 30 minutes, before decreasing faster than observed experimentally. The plateau level ($E_{max}$) is subject to systematic variability across replicate (Fig 7), potentially due to variability in receptor and/or sensor expression (the validation data seems to have reached saturation phase starting from *L* = 3 nM, which is not the case of the experimental data used for fitting). Finally, one notable model limitation emerges in the pulse-chase condition: following ligand washout, the number of membrane receptors first decreases rapidly due to internalization, then rebounds after approximately 20 minutes, presumably reflecting the fraction of receptors recycled back to the plasma membrane (Fig 11 - right column). This biphasic dynamic is not captured by our model, possibly due to the large experimental variability that obscures the underlying trend - most data points nonetheless remain within the confidence interval derived from the statistical observation model. The observed decrease in plasma membrane receptors can be attributed to both internalization and desensitization reactions. When we simulated the model 3.1 with its best fit parameters, but with no desensitization ($k_{des}^{1}=0$) and with a faster internalization ($k_{i12}\times 500$), we were able to reproduce the bounce effect (Fig 12). Similarly, this phenomenon appears when we simulated a model without desensitization at the plasma membrane (Fig 12, model 3.60/best fit), with a faster internalization rate ($k_{i12}\times 2$). Overall, this result demonstrates that our set of models can ultimately predict this behavior when considering an alternative set of parameters.

**Fig 11:**
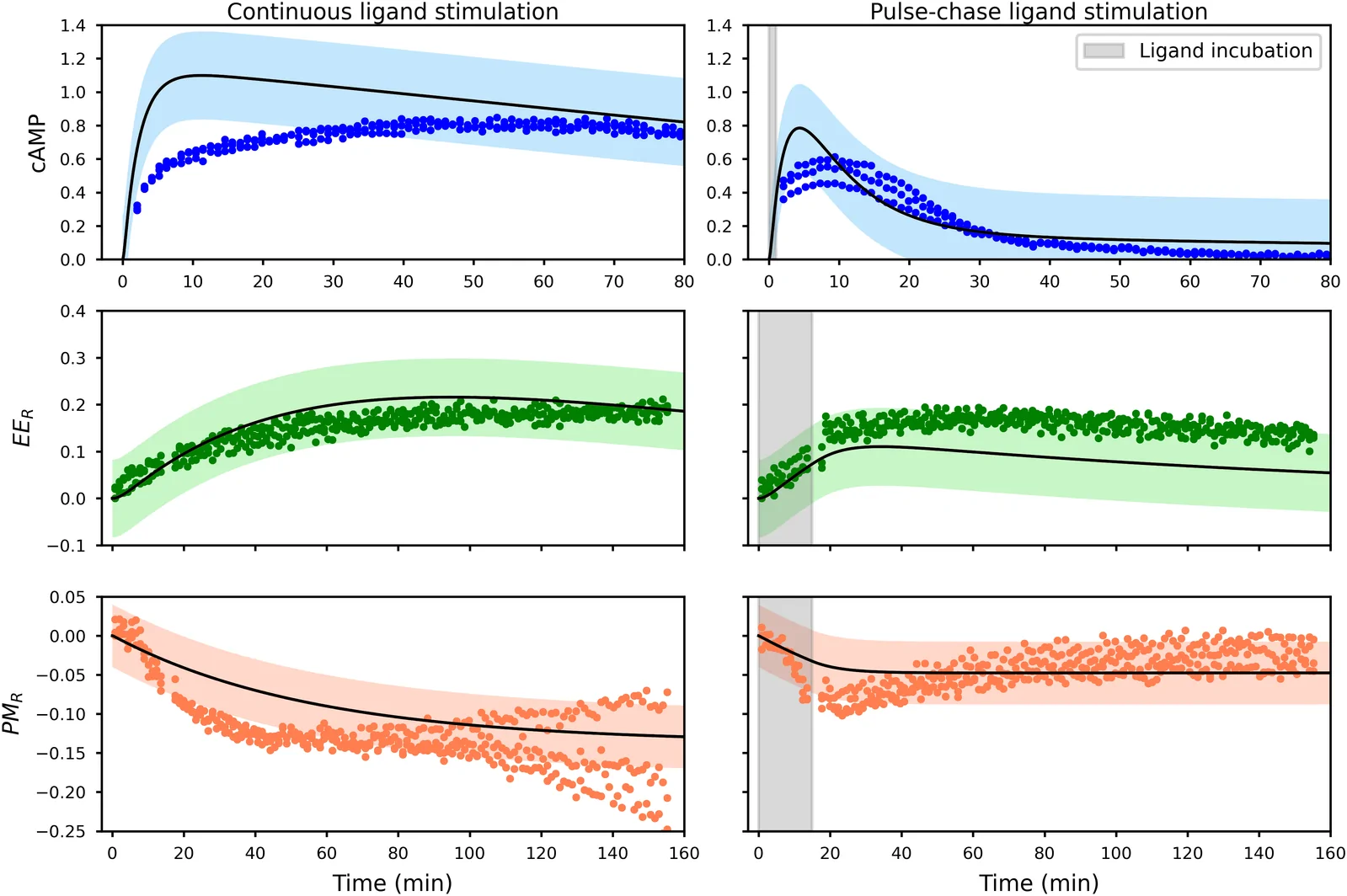
Prediction of observables with data validation for the five best models. The first column shows results under continuous ligand stimulation, and the second column under pulse-chase stimulation, both at a single FSH concentration of 30 nM. The first row displays cAMP levels, followed by *LR*_3_-FYVE (second row) and plasma membrane receptors-CAAX (third row). Scatter points represent validation data under both conditions (continuous and pulse-chase stimulation). Shaded areas indicate the 95% confidence interval of the statistical observation model across the five best models. The parameter values used for these predictions correspond to the best estimates obtained during model selection (Table C in S1 Text).

**Fig 12:**
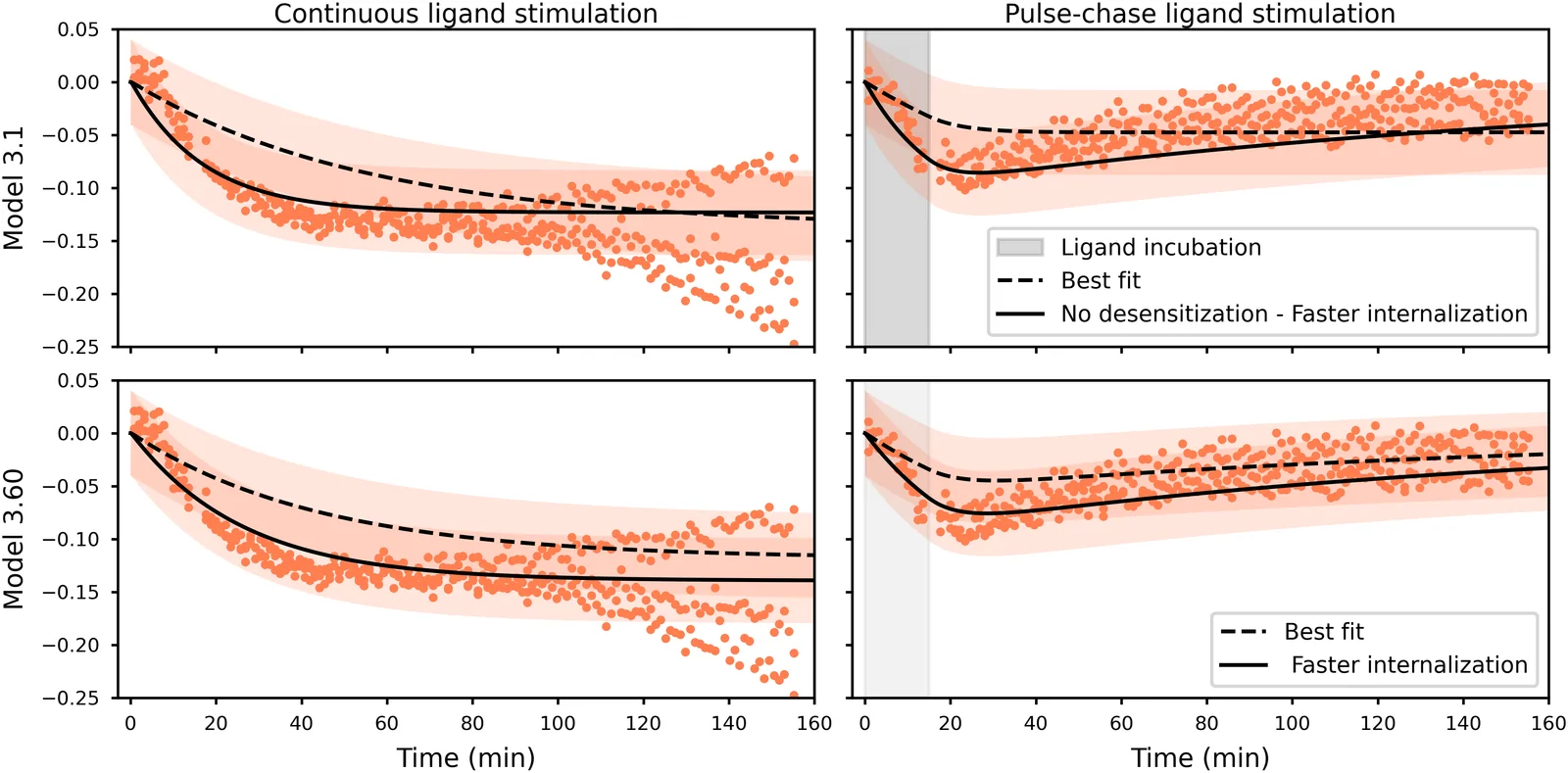
Predictions of plasma membrane receptors. Simulations were performed for both the best-selected model (3.1) and a similar model lacking desensitization from the plasma membrane (3.60). Parameters used for the prediction are: 1) Model 3.1: best estimates (dashed line) and $500\cdot k_{i12}$ and $k_{des}^{1}=0$ (solid line) and 2) Model 3.60: best estimates (dashed line) and $2\cdot k_{i12}$ (solid line).

#### The model accurately predicts binding affinity.

We note that the binding affinity of FSH is predicted at $K_{D}=0.598$ nM, (95% confidence interval: [0.53,0.63] nM), consistently with previously reported values [34]. We additionally designed an experiment to further validate our binding affinity prediction. We characterized FSH binding to its cognate receptor using a first classical binding assay with a recombinant FSH fused to mNeonGreen as a BRET acceptor (FSH^mNG^) and a FSHR fused to a N-terminal NanoLuciferase tag (Nluc-FSHR) as the BRET donor. We then performed a competitive binding experiment where the concentration of FSH^mNG^ was held fixed while unlabelled FSH was added in excess (Fig A in S1 Text, last row). This approach allowed indirect quantification of reference FSH binding through the dose-dependent decrease in FSH^mNG^-induced BRET signal. The first binding assay corresponds to the traditional equilibrium function with *f* the amount of ligand-receptor complexes:


$$\begin{array}{c} f(L^{*})=\frac{L^{*}}{K_{D}^{*}+L^{*}}\;, \end{array}$$ (10)

where $L^{*}$ is the fluorescent FSH^mNG^ concentration, and $K_{D}^{*}$ its binding affinity to FSHR, while the competitive assay corresponds to:


$$\begin{array}{c} f(L)=\frac{K_{D}\cdot L^{*}}{K_{D}\cdot K_{D}^{*}+L\cdot K_{D}^{*}+L^{*}\cdot K_{D}}\;. \end{array}$$ (11)

We jointly optimized Eqs. (10)-(11) in $K_{D}^{*}$, with $K_{D}$ fixed to its model-prediction ($K_{D}=0.583$ nM in the best model) with a least squares fitting. Given the good data-fitting in Fig 13 and the fact that the fitted $K_{D}^{*}$ ($K_{D}^{*}=5.937$ nM) is very close to the *EC*_50_ measured in the experiment, we conclude that the model-predicted $K_{D}$ value is coherent with our competitive binding assays.

**Fig 13:**
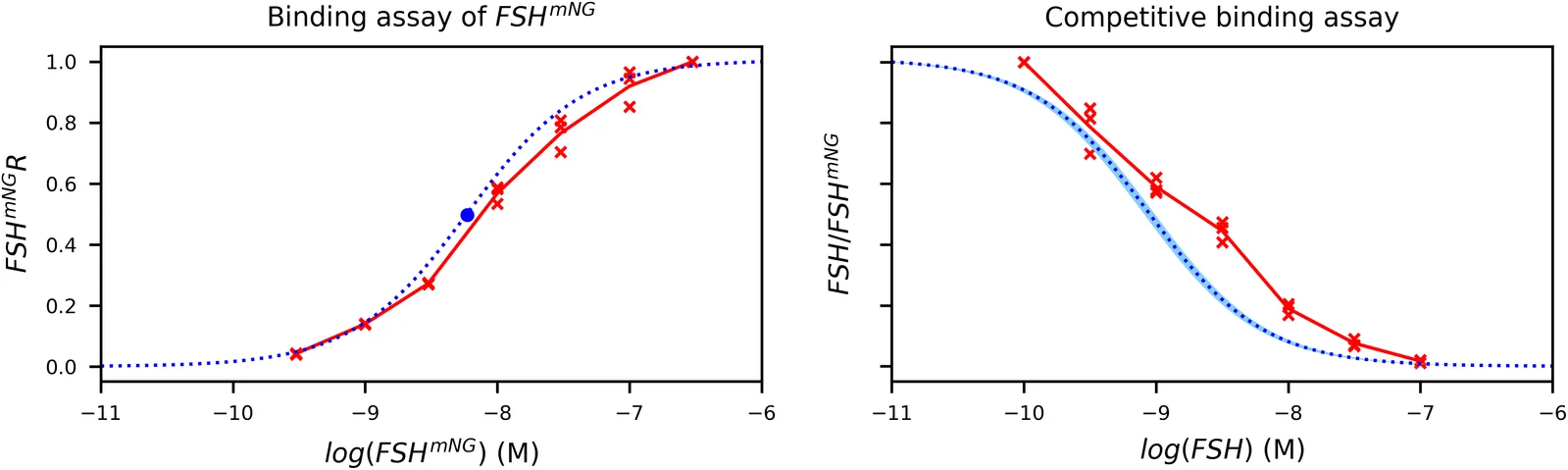
Validation of the FSH binding affinity. Binding assay of FSH^mNG^ to FSHR (left panel) and competitive binding of FSH to FSHR in presence of FSH^mNG^ (right panel, FSH^mNG^ = 3 nM). Data points are shown in red crosses, the solid red line represents the mean of the data, and the dotted blue line corresponds to the prediction. The fitted FSH^mNG^ binding affinity constant is shown by the circle on the left panel.

## Discussion

The classical framework of GPCR signaling, in which receptor activation at the plasma membrane initiates downstream signaling cascades before being terminated through receptor internalization, has been substantially revised. For a growing number of GPCRs, internalization does not simply terminate signaling. Rather, receptors remain active within endosomal compartments, where they promote signaling responses that are qualitatively distinct from those initiated at the plasma membrane - a spatial organization that appears essential for appropriate physiological responses [3,9,10]. Nevertheless, many aspects of receptor trafficking remain poorly understood, and current experimental approaches face inherent limitations in disambiguating the respective contributions of plasma membrane and endosomal signaling to the overall cellular response. In this context, a mathematical modeling approach offers a valuable complementary framework. By deriving and analyzing theoretical results, such an approach can reveal the impact of receptor trafficking on cellular responses in a general framework, broadly applicable to GPCRs. Analysis of dose-response profiles provides a mean to characterize cellular responses across different receptor systems and to evaluate the effects of perturbations on the signaling output.

Several simplifying assumptions were made to ensure analytical tractability and interpretability of the dose-response relationships. First, consistent with standard pharmacological modeling practices [13,25], the ligand was assumed to be in large excess relative to the receptor amount, such that its concentration can be treated as constant, yielding a linear ligand-receptor association rate. Second, ligand and receptor degradation were neglected, as including such terms would preclude positive steady states. Third, the rate of second messenger degradation was assumed to be identical across compartments, despite potentially distinct production rates. Fourth, constitutive receptor activity, although potentially present, was ignored. Finally, ligand-receptor dissociation within intracellular compartments was not considered. The equilibrium between ligand binding and dissociation appears to differ inside endosomes compared to the plasma membrane, for two main reasons: (1) the small size of endosomes, which confines ligand-receptor complexes within a very restricted volume, favoring fast association/dissociation kinetics and rapid convergence toward equilibrium; and (2) differences in local environmental conditions, such as pH, although such information remains unavailable to date. Instead, we assumed that ligand dissociation occurs concomitantly with receptor recycling to the plasma membrane. All those assumptions were made in order to reduce the number of free parameters and improve interpretability. One can obtain analogue of formulas (1)-(15) for (i) nonlinear cAMP production rate; (ii) compartment-dependent cAMP degradation rate; (iii) time-dependent ligand association kinetics (L+R⟷LR); (iv) ligand dissociation in endosomes prior to recycling. These alternative modeling choices will not modify direct estimation of efficacy and potency but will impact their interpretation, as we have shown that ligand binding kinetics, receptor trafficking and compartmentalized signaling all impact observed potency and efficacy, especially in case of internalization inhibition.

Our analysis reveals counter-intuitive effects of receptor trafficking and spatial distribution on the observed *EC*_50_. When studying the impact of receptor trafficking on the signaling response, researchers routinely disrupt dynamin or clathrin activity through the use of drugs such as Dyngo4a and PitStop2 [10], or via overexpressing a dominant negative mutant protein like DynK44E [29]. Recently, it has been shown that inhibiting endocytosis by overexpressing DynK44E leads to a significant decrease in ligand-induced cAMP response for β2-adrenergic receptor (β2AR) and vasoactive intestinal peptide receptor 1 (VIPR1) [29]. Further, this strategy had no effect on potency for these receptors. In the light of our results (Fig 2), this suggests that for β2AR and VIPR1, the endocytic response is strong ($k_{2}^{+}>k_{1}^{+}$), and ligand dissociation and receptor recycling occur on a similar timescale ($k_{off}\approx k_{r21}$). By constrast, for the luteinizing hormone receptor (LHR), inhibiting endocytosis with chemical compounds has been reported to induce a strong increase of the observed potency, with no or moderate impact on the efficacy for both hCG- and LH-induced cAMP response [10]. This suggests that for the LHR, the compartmentalized cAMP production is balanced ($k_{1}^{+}\approx k_{2}^{+}$), but receptor recycling is slower than ligand unbinding ($k_{off}>k_{r21}$) (“shift to the right,” Fig 2).

Several authors have shown that LHR and FSHR are internalized into different types of endosomes (*e.g.,* VEE and EE), with different downstream signaling efficiency and recycling kinetics [6,8]. In [24], the authors designed an FSHR-specific intracellular antibody fragment (i.e., an intracellular VHH), which drives receptor accumulation in EEs at the expense of VEEs (increased *LR*_3_ at the expense of *LR*_2_), associated to a decreased of overall receptor recycling. Interestingly, this innovative tool had no effect on receptor internalization, thus providing a way to finely tune ligand-induced receptor endosomal distribution. Receptor accumulation in EEs was associated with a reduction in global cAMP production. This observation suggests that signaling from EEs is less efficient than that originating from VEEs or the plasma membrane - consistent with a decrease in $E_{max}$ (Eq. (19), Fig 4C and 4D). The fact that the recycling rate from EEs is presumably lower than the one from VEEs ($k_{r21}>k_{r31}$) suggests a potential shift to the left (decrease of *EC*_50_), which constitutes an experimentally testable prediction for future model validation (Fig 4A and 4C). More broadly, Eq. (19) - which integrates trafficking and signaling production parameters to determine the conditions under which *EC*_50_ increases - underscores the complexity of GPCR signaling when three spatially distinct pools of active ligand-receptor complexes coexist. This complexity renders biological interpretation challenging, and calls for the joint use of dedicated mathematical modeling and carefully designed experimental approaches, to rigorously disentangle the interrelationships between receptor trafficking kinetics and signaling response dynamics.

Better understanding spatial dynamics of gonadotropins-induced responses could improve our knowledge of the complex signaling networks it regulates, and therefore help improving the design of therapeutic compounds in reproductive medicine. The parameter estimation framework developed for the FSHR case study allowed us to determine the order of magnitude of most kinetic parameters, which were, to the best of our knowledge, previously unknown. In particular, the joint analysis of kinetic dose-response experiments - encompassing receptor internalization kinetics under both control and perturbed conditions - together with an exhaustive model selection procedure, supports the hypothesis that the FSHR is actively signaling from the plasma membrane and two distinct endosomal compartments, each displaying different signaling kinetics. VEEs appear to account for the largest proportion of cAMP production following FSH stimulation, while the cAMP induced by receptors in EEs emerges in a second, delayed phase (Fig 9). This is consistent with earlier biological studies suggesting that very early endosomes constitute the primary active intracellular signaling compartment [6,8], although definitive experimental confirmation is still lacking. This result echoes the findings of [19], whose authors quantified the relative contribution of each endocytic compartment to RTK signaling across eight receptors, identifying endocytic vesicles as the dominant signaling compartment, accounting for over 43% of total signaling. Despite the mechanistic differences between RTK and GPCR endocytic signaling, this convergence suggests that compartment-specific signaling contributions may follow shared organizational principles across receptor families.

At present, it is not possible to determine a unique model, but rather clusters of plausible models. Among the best-fitting models, the main differences lie in the quantity of ligand-receptor complexes within intracellular compartments and in the magnitude of internalization and recycling fluxes. One way to distinguish between alternative models is to perform additional perturbation experiments such as biasing receptor spatial distribution (Fig 10). Consistently with [24], when receptor accumulates in the third compartment, the best model 3.1 leads to a decrease in efficacy (Fig 10), and its parameter range confirms Eq. (19) (Fig 4): the number of active receptors is higher for *LR*_1_ and *LR*_2_ than for *LR*_3_ (Table C in S1 Text). Interestingly, an in-depth parameter identifiability analysis across the cluster of models that contains model 3.1 revealed that substantial intracellular signaling can occur despite a small number of internalized receptors, a configuration arising from slow internalization combined with fast recycling rates. These predictions are biologically plausible, although experimental validation them remains challenging, as precise measurements of receptor internalization and recycling kinetics are difficult to obtain.

Some studies have pioneered the quantification of receptor trafficking and the number of receptor-positive endosomes labeled with specific compartment markers - such as APPL1 for very early endosomes (*LR*_2_/VEE) and EEA1 for early endosomes (*LR*_3_/EE). In [35], 42% and 36% of FSHR-positive endosomes where positively labelled for APPL1 and EEA1, respectively. Due to the high model uncertainty, direct comparison of receptor proportions is not possible, but our results indicate that a similar order of magnitude of *LR*_2_/VEE and *LR*_3_/EE is coherent with the model (confidence interval cross each others in Table 2). Previous studies suggest that LH receptors are not recycled from EE back to PM [6,8], which is consistent with the fact that some of the best models (e.g., 3.3, 3.14) lack recycling from EE (and instead have desensitisation from EE). In addition, the study [8] reported that receptor recycling is rapid, peaking at approximately 5 min before reaching a plateau - a finding consistent with the time scales inferred in our model (Table 2). The validity of these inferred time scales is further supported by the ability of the model to predict cAMP kinetics under ligand pulse-chase validation experiments.

The role of receptor desensitization in our modeling approach merits further discussion: when receptor desensitization at the PM is present, the quantity of receptors recycling back to the PM cannot be accurately predicted (Fig 12), highlighting the importance of resolving this model default with additional data in future works. Further model validation could be achieved through experimental measurement of internalization and recycling kinetics via single-particle tracking, as well as quantification of receptor proportions in VEEs and EEs by whole-cell microscopy imaging.

A strong prediction of our parameter inference is the large differences in cAMP signaling efficiency between the endosomal and plasma membrane compartments. In future works, the assumption that cAMP degradation is identical in all compartments should be revised. One could wonder indeed if the inferred parameters for the proportion of internalized receptors and the signaling efficiency may be different if local cAMP degradation was taken into account in the model. In addition to receptor trafficking measurements, compartmentalized cAMP biosensors could provide a direct means to test this hypothesis, but designing such selective sensors - which would require distinct endosomal compartments to be more precisely characterized using specific molecular markers - remains challenging, particularly since VEEs currently lack such markers.

The trafficking of GPCRs was modeled using ordinary differential equation systems describing the temporal evolution of the relevant molecular species. This modeling approach improves our understanding of how cells encode receptor signaling by providing a mechanistic interpretation of underlying biological processes, and can readily be extended to other GPCRs by adjusting the number of compartments and trafficking dynamics. To ensure analytical tractability, several simplifying biological assumptions were made to reduce the number of chemical species, despite the known complexity of the system. This parsimony represents both a strength and a limitation of the model: while it yields an interpretable and generalizable framework, it inevitably omits certain biological interactions. Several natural extensions could be envisioned to progressively relax these assumptions. At the level of receptor-G protein coupling, the G protein cycle could be incorporated through a ternary complex formalism [18], and further enriched by accounting for the subcellular trafficking of G proteins themselves - recent evidence highlighting the role of $G_{\alpha s}$ trafficking in intracellular signaling [10,36] makes this a particularly relevant extension. The role of β-arrestins, whose involvement in receptor internalization has recently been substantially revised, also warrants explicit consideration [37–41]. Finally, the framework could be extended to additional subcellular compartments, irrespective of whether signaling responses are produced therein [17].

We employed simplified, or ’toy’, models of receptor signaling cascades to isolate and precisely characterize the specific contribution of receptor trafficking to the overall cellular response. Since the introduction of the operational model, numerous mathematical frameworks have been developed to capture the complexity of GPCR-dependent signaling, addressing aspects such as constitutive receptor activity, G protein cycling, and the activation of multiple effector pathways [18,42–47]. Yet, to our knowledge, few modeling studies have explicitly focused on receptor trafficking, and existing frameworks may now require re-evaluation in light of the recognized importance of signaling compartmentalization. Beyond receptor trafficking itself, a comprehensive understanding of compartmentalized GPCR signaling requires accounting for the coordinated dynamics of multiple cellular actors. These include the differential involvement of β-arrestins in receptor sorting [48–50], and the regulation of the diffusion of second messengers such as cAMP [51]. Although the modeling of these phenomena remains in its early stages, their integration into a unified framework will be essential for a full mechanistic description of spatially organized signaling.

Finally, the present framework relies on deterministic ODE modeling, which captures the average behavior of a cell population. Kinetic dose-response profiles should therefore be interpreted as mean cellular responses across a population, rather than descriptions of individual cells behavior. With the rapid advances in single-cell imaging and single-cell resolution measurements of signaling pathways, the development of stochastic models represents a promising extension of this work, offering a principled framework for characterizing cell-to-cell variability in signaling responses [52,53].

## Materials and methods

### Biological assumptions for modeling

For all the models 1, 2 and 3 (Figs 1, 3 and 5) general assumptions were:

1. the quantity of ligand (*L*) was considered to be in excess compared to the total quantity of receptors and was therefore taken as a constant, resulting in ligand kinetics that are independent of time [13,25,54];
2. ligand and receptor degradations were ignored;
3. the second messenger (cAMP) was produced in each compartment proportionally to the ligand-receptor complexes, but was degraded linearly independently of the compartment type ($\frac{cAMP_{i}}{dt}=k_{i}^{+}LR_{i}-k^{-}cAMP_{i}$);
4. For time-dependent solutions (model 3 (Fig 5)), we started with an initial quantity of free receptors at the plasma membrane ($R_{1}(0)=R_{0}$) and the initial quantity of all other species was null;
5. there was no dissociation of the ligand-receptor complexes in the intracellular compartments. Instead, we assumed that ligand dissociation occurs concomitantly with receptor recycling to the plasma membrane.

In addition, ligand-receptor complexes degradation was neglected in the models 1 and 2 (Figs 1 and 3).

### Mathematical modeling

All the models in our study were studied using the ordinary differential equation formalism, following the evolution concentration of species over time. Then, the equilibrium of each system was calculated to obtain to study the influence of each parameters on the dose-response.

#### Model 1 (Fig 1).

Model 1 (Fig 1) leads to the following ODE system:


$$\begin{array}{c} \left\{ \begin{array}{c} \frac{dR_{1}}{dt}=-L\cdot k_{on}R_{1}+k_{off}LR_{1}+k_{r21}LR_{2},\\ \frac{dLR_{1}}{dt}=L\cdot k_{on}R_{1}-\left(k_{off}+k_{i12}\right)LR_{1},\\ \frac{dLR_{2}}{dt}=k_{i12}LR_{1}-k_{r21}LR_{2},\\ \frac{cAMP_{1}}{dt}=k_{1}^{+}LR_{1}-k^{-}cAMP_{1},\\ \frac{cAMP_{2}}{dt}=k_{2}^{+}LR_{2}-k^{-}cAMP_{2},\\ R_{1}+LR_{1}+LR_{2}=R_{0}. \end{array}\right. \end{array}$$ (12)

In short, through binding (resp. unbinding) reactions at rate $k_{on}$ (resp. $k_{off}$), lead to the formation of a ligand-receptor complex (*LR*_1_) at the plasma membrane. This complex is then internalized into the second compartment at rate $k_{i12}$, while the receptor is recycled back to the membrane at rate $k_{r21}$. The second messenger (cAMP) is produced in each compartment, in proportion to the amount of ligand-receptor complex $LR_{i}$, with *i* = 1,2 with compartment-specific production rates $k_{1}^{+}$ at the plasma membrane and $k_{2}^{+}$ in the intracellular compartment and is degraded according to first-order kinetics, at a rate *k*^–^. Table A in S1 Text summarizes all variables and parameters of this model.

By mass conservation, the receptor trafficking subsystem ($R_{1},LR_{1},LR_{2}$) can be reduced to a two-dimensional affine system whose system matrix is stable (by simple calculations). Thus, the receptor variables are globally exponentially stable, and the same holds for the second messenger cAMP, due to its first-order degradation kinetics. To compute the steady state, consider the system:


$$\begin{array}{c} \left\{ \begin{array}{c} 0=-L\cdot k_{on}R_{1}+k_{off}LR_{1}+k_{r21}LR_{2},\\ 0=L\cdot k_{on}R_{1}-\left(k_{off}+k_{i12}\right)LR_{1},\\ LR_{2}=R_{0}-R_{1}-LR_{1}, \end{array}\right. \end{array}$$

we deduced thanks to simple algebraic calculations the expressions:


$$\begin{array}{c} \left\{ \begin{array}{c} R_{1}=\frac{k_{off}+k_{i12}}{L\cdot k_{on}}LR_{1},\\ LR_{2}=\frac{k_{i12}}{k_{r21}}LR_{1},\\ LR_{1}=\frac{L\cdot R_{0}}{L\left(1+\frac{k_{i12}}{k_{r21}}\right)+K_{D}\left(1+\frac{k_{i12}}{k_{off}}\right)}, \end{array}\right. \end{array}$$

and then using:


$$\begin{array}{c} k^{-}cAMP=k_{1}^{+}LR_{1}+k_{2}^{+}LR_{2}, \end{array}$$

we obtained Eq. (1). The later Eq. (1) has a similar expression as the standard phenomenological dose-response curve which we recall:


$$\begin{array}{c} f(L)=E_{max}\frac{L}{L+EC_{50}}. \end{array}$$ (13)

In Eq. (13), $E_{max}$ quantifies the ligand efficacy (maximum of the cellular response) and *EC*_50_ is the ligand potency (quantity of ligand needed to obtain half of the maximum of the dose response) [20]. Identifying $E_{max}$ and *EC*_50_ in Eq. (1) leads to Eqs. (2)-(3).

#### Model 2 (Fig 3).

Model 2 (Fig 3) leads to the following ODE system (Eq. (14)):


$$\begin{array}{c} \left\{ \begin{array}{c} \frac{dR_{1}}{dt}=-k_{on}L\cdot R_{1}+k_{off}LR_{1}+k_{r21}LR_{2}+k_{r31}LR_{3},\\ \frac{dLR_{1}}{dt}=k_{on}L\cdot R_{1}-\left(k_{off}+k_{i12}+k_{i13}\right)LR_{1},\\ \frac{dLR_{2}}{dt}=k_{i12}LR_{1}-\left(k_{r21}+k_{r23}\right)LR_{2},\\ \frac{dLR_{3}}{dt}=k_{i13}LR_{1}-k_{r31}LR_{3}+k_{r23}LR_{2},\\ \frac{cAMP_{1}}{dt}=k_{1}^{+}LR_{1}-k^{-}cAMP_{1},\\ \frac{cAMP_{2}}{dt}=k_{2}^{+}LR_{2}-k^{-}cAMP_{2},\\ \frac{cAMP_{3}}{dt}=k_{3}^{+}LR_{3}-k^{-}cAMP_{3},\\ R_{1}+LR_{1}+LR_{2}+LR_{3}=R_{0}. \end{array}\right. \end{array}$$ (14)

In short, binding and unbinding reactions, occurring at rates $k_{on}$ and $k_{off}$ respectively, lead to the formation of a ligand-receptor complex (*LR*_1_) at the plasma membrane. The ligand-receptor complex *LR*_1_ is then internalized in two intracellular compartments, called *LR*_2_ or *LR*_3_, at rates $k_{i12}$ and $k_{i13}$, respectively. *LR*_2_ is recycled at rate $k_{r21}$ (respectively *LR*_3_ at rate $k_{r31}$) and receptors in the second compartment, called *LR*_2_, can transfer to the third compartment, called *LR*_3_, at rate $k_{r23}$. The second messenger (cAMP) is produced in each compartment proportionally to the ligand-receptor complex $LR_{i}$ with compartment-specific production rates $k_{i}^{+}$ for *i* = 1,2,3, and is degraded according to first-order kinetics, at a rate *k*^–^. Table A in S1 Text summarizes all variables and parameters of this model. Using the same methodology as the previous section, we derived the analytical formula for the steady-state cAMP response, which reads as follows. By simple algebraic calculations, we obtained the following expressions:


$$\begin{array}{c} \left\{ \begin{array}{c} R_{1}=\frac{k_{off}+k_{i12}+k_{i13}}{k_{on}L}LR_{1},\\ LR_{2}=\frac{k_{i12}}{k_{r21}+k_{r23}}LR_{1},\\ LR_{3}=\left(\frac{k_{i13}}{k_{r31}}+\frac{k_{r23}}{k_{r31}}\frac{k_{i12}}{k_{r21}+k_{r23}}\right)LR_{1},\\ LR_{1}=\frac{L\cdot R_{0}}{L\left(1+\frac{k_{i12}}{k_{r21}+k_{r23}}+\frac{k_{i13}}{k_{r31}}+\frac{k_{r23}}{k_{r31}}\frac{k_{i12}}{k_{r21}+k_{r23}}\right)+K_{D}\left(1+\frac{k_{i12}+k_{i13}}{k_{off}}\right)}. \end{array}\right. \end{array}$$

The steady-state for cAMP is the following:


$$\begin{array}{c} cAMP=\frac{LR_{0}\left(\frac{k_{1}^{+}}{k^{-}}+\frac{k_{i12}}{k_{r21}+k_{r23}}\left(\frac{k_{2}^{+}}{k^{-}}+\frac{k_{3}^{+}}{k^{-}}\frac{k_{r23}}{k_{r31}}\right)+\frac{k_{3}^{+}}{k^{-}}\frac{k_{i13}}{k_{r31}}\right)}{L\left(1+\frac{k_{i12}}{k_{r21}+k_{r23}}\left(1+\frac{k_{r23}}{k_{r31}}\right)+\frac{k_{i13}}{k_{r31}}\right)+K_{D}\left(1+\frac{k_{i12}+k_{i13}}{k_{off}}\right)}, \end{array}$$ (15)

with the values of potency and efficacy:


$$\begin{array}{c} E_{max}=\frac{R_{0}\frac{k_{1}^{+}}{k^{-}}+\frac{k_{i12}}{k_{r21}+k_{r23}}\left(\frac{k_{2}^{+}}{k^{-}}+\frac{k_{3}^{+}}{k^{-}}\frac{k_{r23}}{k_{r31}}\right)+\frac{k_{3}^{+}}{k^{-}}\frac{k_{i13}}{k_{r31}}}{1+\frac{k_{i12}}{k_{r21}+k_{r23}}\left(1+\frac{k_{r23}}{k_{r31}}\right)+\frac{k_{i13}}{k_{r31}}}, \end{array}$$ (16)

$$\begin{array}{c} EC_{50}=K_{D}\left(\frac{1+\frac{k_{i12}+k_{i13}}{k_{off}}}{1+\frac{k_{i12}}{k_{r21}+k_{r23}}\left(1+\frac{k_{r23}}{k_{r31}}\right)+\frac{k_{i13}}{k_{r31}}}\right). \end{array}$$ (17)

Now using $k_{i12}=(1-p_{23})\cdot k_{int}$ and $k_{i13}=p_{23}\cdot k_{int}$, we obtained efficacy, potency and transducer ratio values given in Eqs. (6)-(8). We studied the effect of increasing the proportion of receptors in the third compartment (*p*_23_) on the efficacy $E_{max}$, potency (*EC*_50_) and τ (Eqs. (6)-(8)), to obtain the following necessary and sufficient conditions:


$$\begin{array}{c} EC_{50}\text{ is increasing according to }p_{23}⟺k_{r21}<k_{r31}, \end{array}$$ (18)

$$\begin{array}{c} E_{max}\text{ is increasing according to }p_{23}⟺k_{2}^{+}\left(k_{r31}+k_{int}\right)+k_{1}^{+}k_{r21}<k_{3}^{+}\left(k_{r21}+k_{int}\right)+k_{1}^{+}k_{r31}, \end{array}$$ (19)

and,


$$\begin{array}{c} \tau \text{ is increasing according to }p_{23}⟺k_{3}^{+}k_{r21}>k_{2}^{+}k_{r31}. \end{array}$$ (20)

#### Model 3 (Fig 5).

The general model 3 (Fig 5) for the parameter estimation problem is the following ODE system (Eq. (21) and Table A in S1 Text):


$$\begin{array}{c} \left\{ \begin{array}{c} \frac{dR_{1}}{dt}=-k_{on}L\cdot R_{1}+k_{off}LR_{1}+k_{r21}LR_{2}+k_{r31}LR_{3},\\ \frac{dLR_{1}}{dt}=k_{on}L\cdot R_{1}-\left(k_{off}+k_{i12}+k_{i13}+k_{des}^{1}\right)LR_{1},\\ \frac{dLR_{2}}{dt}=k_{i12}LR_{1}-\left(k_{r21}+k_{r23}\right)LR_{2},\\ \frac{dLR_{3}}{dt}=k_{i13}LR_{1}-\left(k_{r31}+k_{des}^{3}\right)LR_{3}+k_{r23}LR_{2},\\ \frac{dcAMP_{1}}{dt}=k_{1}^{+}LR_{1}-k^{-}cAMP_{1},\\ \frac{dcAMP_{2}}{dt}=k_{2}^{+}LR_{2}-k^{-}cAMP_{2},\\ \frac{dcAMP_{3}}{dt}=k_{3}^{+}LR_{3}-k^{-}cAMP_{3},\\ \frac{dR_{1}^{ps_{2}}}{dt}=-k_{on}L\cdot R_{1}^{ps_{2}}+k_{off}LR_{1}^{ps_{2}}+k_{r21}LR_{2}^{ps_{2}}+k_{r31}LR_{3}^{ps_{2}},\\ \frac{dLR_{1}^{ps_{2}}}{dt}=k_{on}L\cdot R_{1}^{ps_{2}}-\left(k_{off}+ps_{2}(k_{i12}+k_{i13})+k_{des}^{1}\right)LR_{1}^{ps_{2}},\\ \frac{dLR_{2}^{ps_{2}}}{dt}=ps_{2}k_{i12}LR_{1}^{ps_{2}}-\left(k_{r21}+k_{r23}\right)LR_{2}^{ps_{2}},\\ \frac{dLR_{3}^{ps_{2}}}{dt}=ps_{2}k_{i13}LR_{1}^{ps_{2}}-\left(k_{r31}+k_{des}^{3}\right)LR_{3}^{ps_{2}}+k_{r23}LR_{2}^{ps_{2}},\\ \frac{dcAMP_{1}^{ps_{2}}}{dt}=k_{1}^{+}LR_{1}^{ps_{2}}-k^{-}cAMP_{1}^{ps_{2}},\\ \frac{dcAMP_{2}^{ps_{2}}}{dt}=k_{2}^{+}LR_{2}^{ps_{2}}-k^{-}cAMP_{2}^{ps_{2}},\\ \frac{dcAMP_{3}^{ps_{2}}}{dt}=k_{3}^{+}LR_{3}^{ps_{2}}-k^{-}cAMP_{3}^{ps_{2}},\\ x(0)=(R_{0},0,0,0,0,0,0,R_{0},0,0,0,0,0,0)\;, \end{array}\right. \end{array}$$ (21)

together with the observable-equation system (Eq. (9) in the main text),


$$\begin{array}{c} \left\{ \begin{array}{c} cAMP=\alpha_{1}\left(cAMP_{1}+cAMP_{2}+cAMP_{3}\right),\\ cAMP^{ps_{2}}=\alpha_{1}\left(cAMP_{1}^{ps_{2}}+cAMP_{2}^{ps_{2}}\right)+cAMP_{3}^{ps_{2}},\\ PM_{R}=\alpha_{2}\left(R_{1}+LR_{1}-R_{0}\right),\\ EE_{R}=\alpha_{3}LR_{3}. \end{array}\right. \end{array}$$

The ODE model is exactly the same model as Eq. (14) but includes the desensitization/degradation of *LR*_1_ at the plasma membrane ($k_{des}^{1}$) and of *LR*_3_ in the early endosomes ($k_{des}^{3}$). Table A in S1 Text summarizes all variables and parameters of this model.

### Reparameterization of the equations for the parameter estimation problem

The parameter estimation problem includes different types of parameters, such as initial conditions, kinetic parameters, error parameters and data parameters, totalling 21 parameters (Table 3).

**Table 3:** Table of the parameters of model 3 (Fig 5).

Type	Name	Number
Initial condition	*R*_0_	1
Kinetic parameters	$k_{on}$, $k_{off}$, $k_{r21}$, $k_{r31}$, $k_{i12}$, $k_{i13}$, $k_{r23}$, $k_{1}^{+}$, $k_{2}^{+}$, $k_{3}^{+}$, *k*^–^, $k_{des}^{1}$, $k_{des}^{3}$	13
Drug parameter	*ps*_2_	1
Error parameters	$\sigma_{1}$, $\sigma_{2}$, $\sigma_{3}$	3
Data parameters	$\alpha_{1}$, $\alpha_{2}$, $\alpha_{3}$	3
Total		21

To decrease the number of parameters, the first step is to reparameterized the model. All receptor species quantity were divided by *R*_0_ ($A^{*}=\frac{A}{R_{0}}$, with $A\in R_{1},R_{1}^{ps_{2}},LR_{i},LR_{i}^{ps_{2}},i=1,2,3$), and all cAMP species quantity were multiplied by $\alpha_{1}$ ($A^{*}=\alpha_{1}A$, with $A\in cAMP_{i},cAMP_{i}^{ps_{2}},i=1,2,3$). Further, we chose $k_{off}$ as the time scale parameter, and we noted $k^{*}$ the normalized parameters: $k^{*}=\frac{k}{k_{off}}$. We obtained the reparametrized system (Eq. (22)) (the * notations on the variables was omitted for simplicity):


$$\begin{array}{c} \left\{ \begin{array}{c} \frac{dR_{1}}{dt}=k_{off}\left(-\frac{1}{K_{D}}L\cdot R_{1}+LR_{1}+k_{r21}^{*}LR_{2}+k_{r31}^{*}LR_{3}\right),\\ \frac{dLR_{1}}{dt}=k_{off}\left(\frac{1}{K_{D}}L\cdot R_{1}-\left(1+k_{i12}^{*}+k_{i13}^{*}+k_{des}^{1*}\right)LR_{1}\right),\\ \frac{dLR_{2}}{dt}=k_{off}\left(k_{i12}^{*}LR_{1}-\left(k_{r21}^{*}+k_{r23}^{*}\right)LR_{2}\right),\\ \frac{dLR_{3}}{dt}=k_{off}\left(k_{i13}^{*}LR_{1}-\left(k_{r31}^{*}+k_{des}^{3*}\right)LR_{3}+k_{r23}^{*}LR_{2}\right),\\ \frac{dcAMP_{1}}{dt}=k_{off}k^{-*}\left(\lambda_{tot}\lambda_{1}\left(1-\lambda_{3}\right)LR_{1}-cAMP_{1}\right),\\ \frac{dcAMP_{2}}{dt}=k_{off}k^{-*}\left(\lambda_{tot}\left(1-\lambda_{1}\right)\left(1-\lambda_{3}\right)LR_{2}-cAMP_{2}\right),\\ \frac{dcAMP_{3}}{dt}=k_{off}k^{-*}\left(\lambda_{tot}\lambda_{3}LR_{3}-cAMP_{3}\right),\\ \frac{dR_{1}^{ps_{2}}}{dt}=k_{off}\left(-\frac{1}{K_{D}}L\cdot R_{1}^{ps_{2}}+LR_{1}^{ps_{2}}+k_{r21}^{*}LR_{2}^{ps_{2}}+k_{r31}^{*}LR_{3}^{ps_{2}}\right),\\ \frac{dLR_{1}^{ps_{2}}}{dt}=k_{off}\left(\frac{1}{K_{D}}L\cdot R_{1}^{ps_{2}}-\left(1+ps_{2}(k_{i12}^{*}+k_{i13}^{*})+k_{des}^{1*}\right)LR_{1}^{ps_{2}}\right),\\ \frac{dLR_{2}^{ps_{2}}}{dt}=k_{off}\left(ps_{2}k_{i12}^{*}LR_{1}^{ps_{2}}-\left(k_{r21}^{*}+k_{r23}^{*}\right)LR_{2}^{ps_{2}}\right),\\ \frac{dLR_{3}^{ps_{2}}}{dt}=k_{off}\left(ps_{2}k_{i13}^{*}LR_{1}^{ps_{2}}-\left(k_{r31}^{*}+k_{des}^{3*}\right)LR_{3}^{ps_{2}}+k_{r23}^{*}LR_{2}\right),\\ \frac{dcAMP_{1}^{ps_{2}}}{dt}=k_{off}k^{-*}\left(\lambda_{tot}\lambda_{1}\left(1-\lambda_{3}\right)LR_{1}^{ps_{2}}-cAMP_{1}^{ps_{2}}\right),\\ \frac{dcAMP_{2}^{ps_{2}}}{dt}=k_{off}k^{-*}\left(\lambda_{tot}\left(1-\lambda_{1}\right)\left(1-\lambda_{3}\right)LR_{2}^{ps_{2}}-cAMP_{2}^{ps_{2}}\right),\\ \frac{dcAMP_{3}^{ps_{2}}}{dt}=k_{off}k^{-*}\left(\lambda_{tot}\lambda_{3}LR_{3}^{ps_{2}}-cAMP_{3}^{ps_{2}}\right),\\ x(0)=(1,0,0,0,0,0,0,1,0,0,0,0,0,0)\;, \end{array}\right. \end{array}$$ (22)

together with the reparameterized observables-equation system:


$$\begin{array}{c} \left\{ \begin{array}{c} cAMP=cAMP_{1}+cAMP_{2}+cAMP_{3},\\ cAMP^{ps_{2}}=cAMP_{1}^{ps_{2}}+cAMP_{2}^{ps_{2}}+cAMP_{3}^{ps_{2}},\\ PM_{R}=\alpha_{2}^{*}\left(R_{1}+LR_{1}-1\right),\\ EE_{R}=\alpha_{3}^{*}LR_{3}, \end{array}\right. \end{array}$$

where we also defined normalized production parameters:


$$\begin{array}{cc} & \lambda_{1}=\frac{k_{1}}{k_{1}+k_{2}},\\ & \lambda_{3}=\frac{k_{3}}{k_{1}+k_{2}+k_{3}},\\ & \lambda_{tot}=\alpha_{1}R_{0}\frac{\left(k_{1}+k_{2}+k_{3}\right)}{k^{-}}, \end{array}$$ (23)

and $\alpha_{2}^{*}=\alpha_{2}R_{0}$ and $\alpha_{3}^{*}=\alpha_{3}R_{0}$.

The number of parameters decreases from 21 to 19, by deleting the initial quantity of receptor, *R*_0_, and by replacing, $k_{1}^{+}$, $k_{2}^{+}$, $k_{3}^{+}$ and $\alpha_{1}$ by $\lambda_{1}$, $\lambda_{3}$ and $\lambda_{tot}$ (Eqs. (23)). The number of parameters reparameterized is summarized in Table 4.

**Table 4:** Table of the reparameterized parameters (Model 3 (Fig 5) and Eq. (22)).

Type	Name	Number
Time scale parameter	$k_{off}$	1
Kinetic parameters	$K_{D}$, $k_{r21}^{*}$, $k_{r31}^{*}$, $k_{i12}^{*}$, $k_{i13}^{*}$, $k_{r23}^{*}$, $\lambda_{tot}$, $\lambda_{1}$, $\lambda_{3}$, $k^{-*}$, $k_{des}^{1*}$, $k_{des}^{3*}$	12
Drug parameter	*ps*_2_	1
Error parameters	$\sigma_{1}$, $\sigma_{2}$, $\sigma_{3}$	3
Data parameters	$\alpha_{2}^{*}$, $\alpha_{3}^{*}$	2
Total		19

### Parameter estimation

The parameter estimation problem was implemented using the Python-based tools PEtab [55] and pyPESTO [56]. PEtab provides a structured framework for parameter estimation by combining several tables, including the network, measurements, conditions, parameters, observables, and visualization results, thereby linking them consistently. This organization makes it easier to modify model parameters rapidly and to associate different measurements with observables under identical biological conditions. pyPESTO is then used to solve the PEtab problem and provides useful tools for analyzing the results. Let $y_{ij}$ denote the *j*-th observable at time $t_{i}$, and let $f_{j}(t_{i};\theta )$ be the corresponding ODE solution. We assume Gaussian noise for each observable, of standard deviation $\sigma_{j}$. The likelihood function is then defined by:


$$\begin{array}{c} ℒ=\prod_{j=1}^{m}\prod_{i=1}^{n_{j}}\frac{1}{\sqrt{2\pi \sigma_{j}^{2}}}\exp\left(-\frac{(y_{ij}-f_{j}(t_{i};\theta ){)}^{2}}{2\sigma_{j}^{2}}\right). \end{array}$$ (24)

The algorithm pyPESTO evaluates the negative log-likelihood function for each parameter vector (θ,σ) (Table 4), defined by:


$$\begin{array}{c} \ell (\theta ,\sigma )=\frac{1}{2}\sum_{j=1}^{m}\sum_{i=1}^{n_{j}}\left[\log(2\pi \sigma_{j}^{2})+\frac{(y_{ij}-f_{j}(t_{i};\theta ){)}^{2}}{\sigma_{j}^{2}}\right]. \end{array}$$ (25)

To resolve the parameter estimation, the algorithm employs deterministic gradient descent with multi-start optimization 1000 runs with random initial parameter values to ensure a satisfactory fit with the optimizer Fides. Several runs reached to the same optimum for the 5 best models leading to algorithm convergence (Fig F in S1 Text).

### Model selection

Our goal was to identify a parsimonious model that achieves robust parameter estimation and addresses key hypotheses. We performed model selection using Petab and pyPESTO, to compute the Akaike Information Criterion:


$$\begin{array}{c} AIC=2k+2\min\ell , \end{array}$$ (26)

where *k* is the number of estimated parameters (including both ODE model parameters θ and noise parameters σ) and ℓ, the negative log-likelihood function (Eq. (25)) and the minimum is taken over the 1000 multi-runs. This criterion penalizes overly complex models. To validate the selected model, we applied the $\Delta_{AIC}$ criterion, which compares each model’s AIC to that of the best models [57]. $\Delta_{AIC}<2$ are considered as likely as the best model; $2<\Delta_{AIC}<7$, as suitable alternatives; $7<\Delta_{AIC}<10$, less relevant; and those with $\Delta_{AIC}>10$ are rejected.

### Structural and practical identifiability

To verify the theoretical stability of the complete model after reparameterization (Eq. (22)), we employed StructuralIdentifiability with output saturation option [32,58,59]. This tool allows us to assess the structural identifiability of the model based on the observable variables, and in the assumption of idealized data.

To evaluate practical identifiability relative to available biological data, we used the Profile Likelihood Estimate (PLE) method [60]:


$$\begin{array}{c} PL_{j}(p)=\min_{\theta \in \{\theta |\theta_{j}=p\}}\ell (y|\theta ), \end{array}$$ (27)

where the negative log-likelihood ℓ (Eq. (25)) is evaluated as a function of the values *p* of a parameter component $\theta_{j}$, while all other parameters $\theta_{i},i\neq j$ are reoptimized. The values comprise in the 95% confidence interval were kept. The profile likelihood estimate for models 3.1 to 3.5 is shown in Fig G in S1 Text.

### Error

Throughout this paper, two types of uncertainties are presented to characterize the data: the statistical error and the profile based error. The statistical error captures the link between the observables and the data. It corresponds to the Gaussian noise model with standard deviation given by the parameters $\sigma_{1},\sigma_{2}$ and $\sigma_{3}$, associated respectively with observables (Eq. (9)). The parameter estimation problem infers the variance of the data with respect to each observable. Consequently, when plotting the observables, it is essential to include the statistical error as follows:


$$\begin{array}{c} O_{i}\pm 0.95\cdot \sigma_{i},\;\text{with }i=1,2,3. \end{array}$$

The profile based 95% confidence interval accounts for the uncertainty in the predictions given the uncertainty in parameters. Since the parameters are each associated with a confidence interval estimated via the Profile Likelihood Estimation (PLE) (Eq. (27)), they do not take a single unique value. It is therefore essential to propagate these uncertainties when computing predictions for quantities other than the observables. The predictions were computed for all parameter values within the confidence interval, and the minimum and maximum values of each prediction were retained to define the uncertainty bounds.

### Dose-response from kinetic simulation

Since Eq. (21) includes irreversible receptor desensitization, all species have a steady-state value of 0, unlike the models 1 and 2 (Figs 1 and 3). Therefore, rather than focusing on the steady state, we quantified the response by taking the area under the curve (AUC). We simulated Eq. (21) over one hour for different internalization rates in order to generate the dose-response profiles shown in Fig 10.

### Estimation of the constant affinity

The least squares algorithm from scipy.optimize was used to fit models Eqs. (10)-(11) to the validation binding experiments, in order to estimate the value of $K_{D}^{*}$.

### Times, probability and proportion

#### General formula.

Endocytosis and recycling events are not almost certain events from a probability point of view given that the model includes irreversible receptor desensitization. Therefore, we calculated conditional expectation times using the following method [61]. Given a continuous-time Markov chain (CTMC) of infinitesimal generator *L* on a state-space Ω∪A∪B where *A* and *B* are two disjoint absorbing ensembles, we calculated the solution of


$$\begin{array}{cc} L(hf)(x) & =-h(x),x\in \Omega \;,\;f(x)=0,x\in A,\\ L(h)(x) & =0,x\in \Omega \;,\;h(x)=1,x\in A,\;h(x)=0,x\in B. \end{array}$$ (28)

In Eq. (28), *h*(*x*) is the absorbing probability in *A*, starting from x∈Ω, and *f* is the conditional expectation time:


$$\begin{array}{c} f(x_{0})=𝔼\left[\tau_{A}|\tau_{A}<\tau_{B},X(0)=x_{0}\right]\;, \end{array}$$

with $\tau_{A}$ and $\tau_{B}$ the absorbing times in *A* et *B* respectively.

#### Endocytosis time and probability.

We used the CTMC whose graph is the following:


$$\begin{array}{c} {\text{R}}_{1}\overset{{\text{k}}_{\text{on}}\text{L}\;}{\to }{\text{LR}}_{1}\overset{{\text{k}}_{\text{off}}\;}{\to }{\text{R}}_{1}\text{ and }{\text{LR}}_{1}\overset{{\text{k}}_{\text{i}12}\;}{\to }{\text{LR}}_{2},{\text{LR}}_{1}\overset{{\text{k}}_{\text{i}13}\;}{\to }{\text{LR}}_{3},{\text{LR}}_{1}\overset{{\text{k}}_{\text{des}}^{1}\;}{\to }\emptyset , \end{array}$$

with $\Omega =\{R_{1},LR_{1}\}$, $A=\{LR_{2},LR_{3}\}$ and B={∅} (the absorbing desensitization state). We obtained for the conditional expectation time for endocytosis, with $x_{0}=R_{1}$:


$$\begin{array}{c} \begin{array}{cc} \text{Endocytosis probability} & =h(R_{1})=h(LR_{1})=\frac{k_{i12}+k_{i13}}{k_{i12}+k_{i13}+k_{des}^{1}}\;,\\ \text{Endocytosis time} & =f(R_{1})=\frac{1}{k_{on}L}+\frac{1}{k_{i12}+k_{i13}+k_{des}^{1}}\frac{\left(L+K_{D}\right)}{L}. \end{array} \end{array}$$

#### Recycling time and probability.

We used the CTMC whose graph is the following:


$$\begin{array}{c} {\text{LR}}_{2}\overset{{\text{k}}_{\text{r}23}\text{L}\;}{\to }{\text{LR}}_{3}\overset{{\text{kr}}_{31}\;}{\to }{\text{R}}_{1}\text{ and }{\text{LR}}_{2}\overset{{\text{k}}_{\text{r}21}\;}{\to }{\text{R}}_{1},{\text{LR}}_{3}\overset{{\text{k}}_{\text{des}}^{3}\;}{\to }\emptyset , \end{array}$$

with $\Omega =\{LR_{2},LR_{3}\}$, *A*={*R*_1_}, and B={∅} (the absorbing desensitization state). We obtained for the endocytosis, with $x_{0}=LR_{2}$ and $x_{0}=LR_{3}$:


$$\begin{array}{c} \begin{array}{cc} h(LR_{3}) & =\frac{k_{r31}}{k_{r31}+k_{des}^{3}},\\ h(LR_{2}) & =\frac{k_{r21}}{k_{r23}+k_{r21}}+\frac{k_{r23}}{k_{r23}+k_{r21}}h(LR_{3}),\\ f(LR_{3}) & =\frac{1}{k_{r31}+k_{des}^{3}},\\ f(LR_{2}) & =\frac{1}{k_{r23}+k_{r21}}+\frac{k_{r23}}{k_{r23}+k_{r21}}\frac{h(LR_{3})}{h(LR_{2})}f(LR_{3}). \end{array} \end{array}$$

Finally, we averaged out these quantities according to the initial point, which is *LR*_2_ with probability $\frac{k_{i12}}{k_{i12}+k_{i13}}$, and *LR*_3_ with probability $\frac{k_{i13}}{k_{i12}+k_{i13}}$. We thus obtained:


$$\begin{array}{c} \begin{array}{cc} \text{Recycling probability} & =\frac{k_{i13}}{k_{i12}+k_{i13}}h(LR_{3})+\frac{k_{i12}}{k_{i12}+k_{i13}}h(LR_{2})\;,\\ \text{Recycling time} & =\frac{k_{i13}}{k_{i12}+k_{i13}}f(LR_{3})+\frac{k_{i12}}{k_{i12}+k_{i13}}f(LR_{2}). \end{array} \end{array}$$

#### Receptors proportion.

The proportion of receptors is:


$$\begin{array}{c} \begin{array}{cc} & LR_{intra}=\frac{LR_{2}+LR_{3}}{R_{1}+LR_{1}+LR_{2}+LR_{3}}\cdot 100,\\ & LR_{2}=\frac{LR_{2}}{LR_{2}+LR_{3}}\cdot 100,\\ & LR_{3}=\frac{LR_{3}}{LR_{2}+LR_{3}}\cdot 100. \end{array} \end{array}$$

### Biological data

All measurements were performed across 10 independent experiments per data type, revealing inter-experiment variability, likely attributable to differences in receptor and/or BRET sensor expression levels.

#### Ligands and drugs.

Recombinant FSH (GONAL-f^R^) was kindly provided by Merck (Darmstadt, Germany) and resuspended in mQ H_2_O. FSH-mNeonGreen (FSH-mNG) was designed in our group and produced in ExpiCHO-S cells expression system. ExpiCHO-S cells at 6.10^6^ living cells/mL density were transfected with 20 μg of pcDNA3.1 encoding the FSH-mNG, according to ExpiCHO-S Expression System (Gibco) manufacturer’s instructions. Cells were cultured for one day at $37^{\circ }$C/8% CO_2_ on a shaker platform (120 rpm) before supplementation with feed and enhancer solution (provided in the ExpiCHO-S expression system transfection kit). Cells were then cultured for 12 days at $32^{\circ }$C/5% CO_2_ at 120 rpm. The culture was centrifuged at 500 g/30 min/$4^{\circ }$C, and the supernatant was collected. The supernatant was then centrifuged at 5000g/30min/$4^{\circ }$C. The newly collected supernatant was dialyzed in regenerated cellulose membrane tubing with a 6–8 kDa molecular weight cut-off (MWCO) (Spectra/Por^M^) against a pH 8.0 buffer (50 mM Tris HCl, 100 mM NaCl) overnight at $4^{\circ }$C under continuous stirring. The dialyzed supernatant was centrifuged (5000g/30min/$4^{\circ }$C) to remove insoluble debris, and passed through a buffer equilibrated (pH 8.0, 50mM TrisHCl, 100mM NaCl) Protino Ni-IDA packed column (Macherey-Nagel). The column was washed with three times its volume of the following buffers: i) pH 8.0, 50mM TrisHCl, 100mM NaCl, ii) pH 8.0, 50mM TrisHCl, 1M NaCl, iii) pH 8.0, 50mM TrisHCl, 100mM NaCl. The FSH-mNG elution was performed with a pH 8.0, 50mM TrisHCl, 100mM NaCl, 500mM imidazole buffer. The FSH-mNG was buffer exchanged into a pH 8.0, 50mM TrisHCl, 100mM NaCl buffer, using 10DG Desalting Prepacked Gravity Flow Columns (BioRad Laboratories). The FSH-mNG was then concentrated using 30kDa MWCO Amicon Ultra centrifugation unit (Sigma-Aldrich) according to the manufacturer’s instructions. After analysis by Coomassie blue SDS-PAGE gel staining, purified FSH-mNG was stored at -$20^{\circ }$C. PitStop2 was purchased at Sigma-Aldrich, and used at a final concentration of 30μM.

#### Plasmids.

The FSHR-RLuc8 plasmid was kindly provided by Pr. Aylin Hanyaloglu (Imperial College London, United Kingdom). All other plasmids were designed in our group and synthesized by Twist Bioscience. The cAMP BRET sensor NLuc-EpacD602A-VV-NES was designed from the cAMP BRET sensor NLuc-Epac-VV [62] by adding a nuclear exclusion signal (NES) sequence and the D602A mutation position to improve the sensor’s dynamic range [63].

#### Cell culture.

Human Embryonic Kidney 293 (HEK293A) (Thermo Fisher Scientific) cells were cultured in DMEM (Eurobio) medium containing Glutabio and NaHCO_3_ and supplemented with 10% heat inactivated fetal bovine serum (FBS) (Eurobio), 100 IU/mL penicillin and 0.1 mg/mL streptomycin (Eurobio). Cells were kept at $37^{\circ }$C in a humidified 5% CO_2_ incubator.

#### Bioluminescence Resonance Energy Transfer (BRET).

Forty thousand HEK293A cells per well were seeded in previously 0.01% poly-lysine coated 96-well plates, and transiently transfected in suspension using Metafectene Pro transfection reagent (Biontex Laboratories) according to the manufacturer’s instructions. DNA quantities used in each type of experiment are detailed in the following table:

**Table:** 

Type of experiment	Quantity of plasmid DNA transfected/well
cAMP measurement	FSHR: 25ng
	cAMP sensor NLuc-EpacD602A-VV-NES: 20ng
Receptor trafficking	FSHR-RLuc8: 90ng
	Lyn-Ypet, Ypet-CAAX or Ypet-FYVE: 20ng
Binding of FSH-mNeonGreen	NLuc-FSHR: 30ng

Of note, Lyn and CAAX motifs were used to address Ypet to the lipid rafts and negative charges of PM, respectively, whereas FYVE motif allowed Ypet addressing to the EEs.

48 hours after transfection, BRET measurements were performed upon addition of 5 μM coelenterazine-H (Interchim) diluted in Ca^2+^/Mg^2+^free PBS, containing no or different concentrations of FSH. For experiments performed in presence of PitStop2, cells were pre-incubated 35 minutes in presence of drug-containing buffer before measurements and ligand stimulation. Signals were recorded for at least 60 minutes with a Mithras LB 943 plate reader (Berthold Technologies GmbH & Co.). BRET ratios were calculated as follows: 480nm/540nm for cAMP experiments; 540nm/480nm for receptor internalization and FSH-mNG binding experiments.

## Supporting information

S1 Text Supplementary information file. This file presents the experimental data used in our study, the variables and parameters used in the different models, the trafficking models considered for model selection, predictions of additional results, the convergence algorithm, the profile likelihood for all parameters, the model selection results, the best-estimated parameter values, a summary of the results, and averaged outputs for the most plausible models.
(PDF)
