# Supplementary material for: Spatiotemporal modeling of GPCR signaling: The role of endosomal dynamics and receptor recycling
Source: PLoS Comput Biol. 2026 Sep 29;22(9):e1014790. doi: 10.1371/journal.pcbi.1014790 (PMC13649145; doi:10.1371/journal.pcbi.1014790)
Supplement: Supplementary file 1 [file pcbi.1014790.s001.pdf]

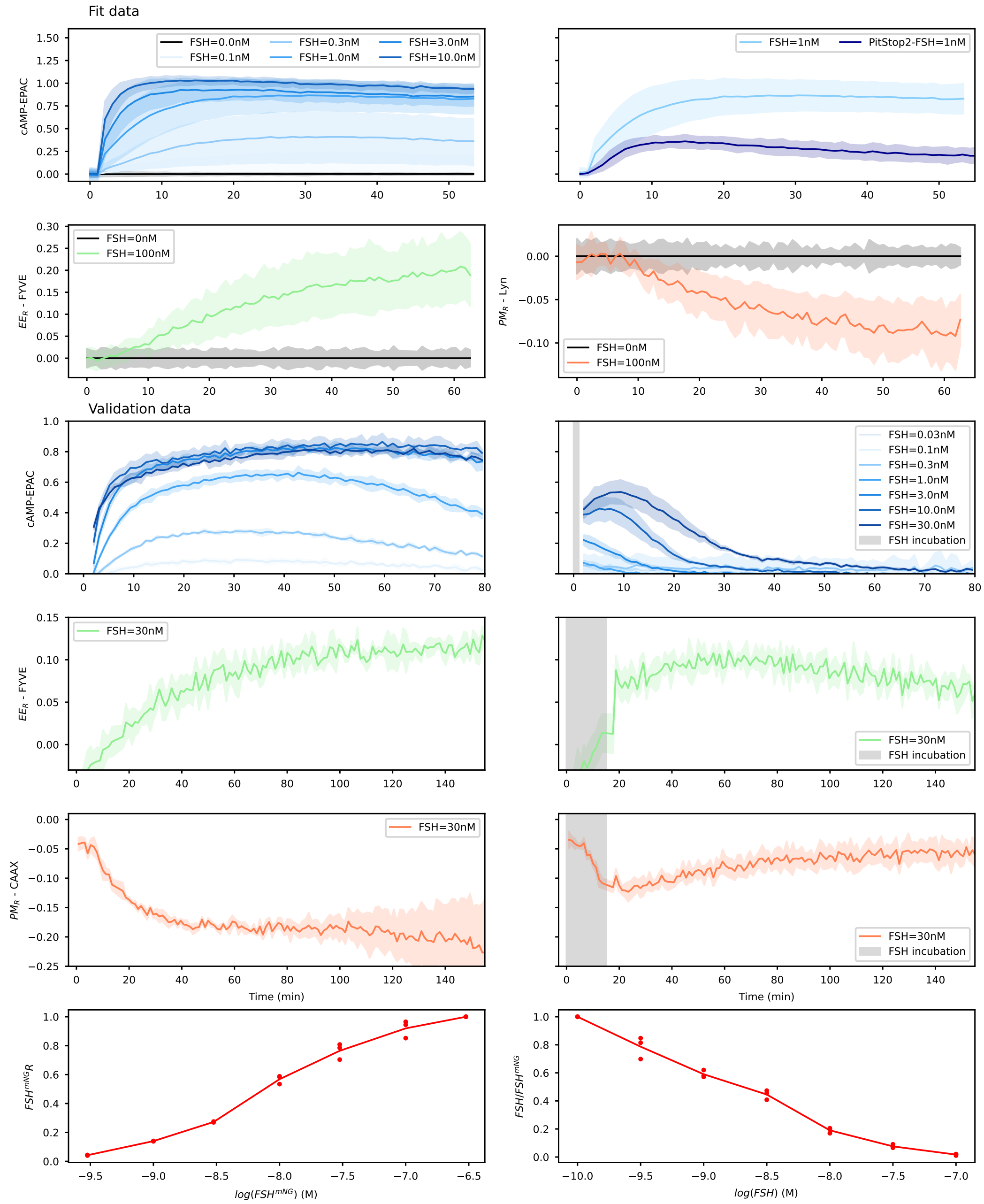

Fig A. Experimental data. The top four panels correspond to the data used to fit the models. The bottom eight panels correspond to validation data. For the model fitting, four different data were included: cAMP levels measured with NLuc-EpacD602A-VV sensor (EPAC) at six ligand concentrations (0, 0.1, 0.3, 1, 3, 10 nM, top left panel) and with CME inhibitor, PitStop2 for FSH= 1 nM (top right panel); Early endosomes localized receptors at two ligand concentrations (0 and 100 nM, Ypet-FYVE, second row left) and plasma membrane localized receptors at two ligand concentrations (0 and 100 nM, Lyn-Ypet, second row right). In each of these four panels model fitting, we have 10 replicates for each dataset. For the model validation, four different data were included: cAMP (EPAC) was measured at seven doses (0, 0.01, 0.3, 1, 3, 10, 30 nM, third row, continuous ligand stimulation in the left column, pulse-chase stimulation in the right); Receptor trafficking at FSH= 30 nM (fourth row: Ypet-FYVE, fifth row: Ypet-CAAX, continuous ligand stimulation in the left column, pulse-chase stimulation in the right). Additional validation data include FSH^{mNG} binding to FSHR at different concentrations and its competition with native FSH for receptor binding (FSH/FSH^{mNG}) (last row).

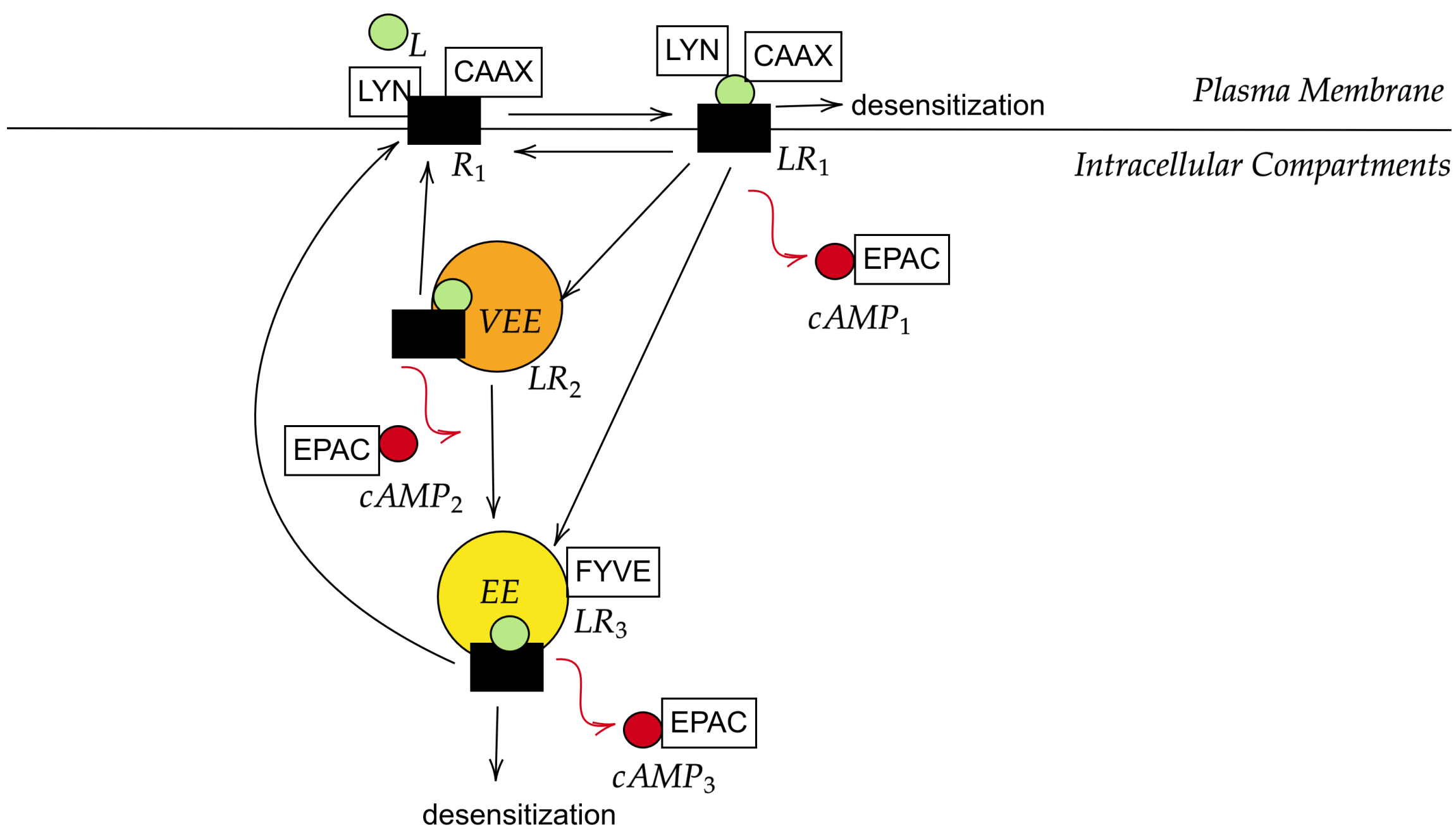

Fig B. Biological Scheme. R_1 represents the amount of receptors at the plasma membrane and LR_1 (respectively LR_2 and LR_3) the amount of complex ligand-receptor at the plasma membrane (respectively in two types of intracellular compartments: very early endosomes and early endosomes). BRET sensing systems are represented in boxed texts: EPAC (cAMP measurement), CAAX and Lyn (receptor localized at plasma membrane) and FYVE (receptors localized at early endosomes). The scheme was done on MatchaIO.

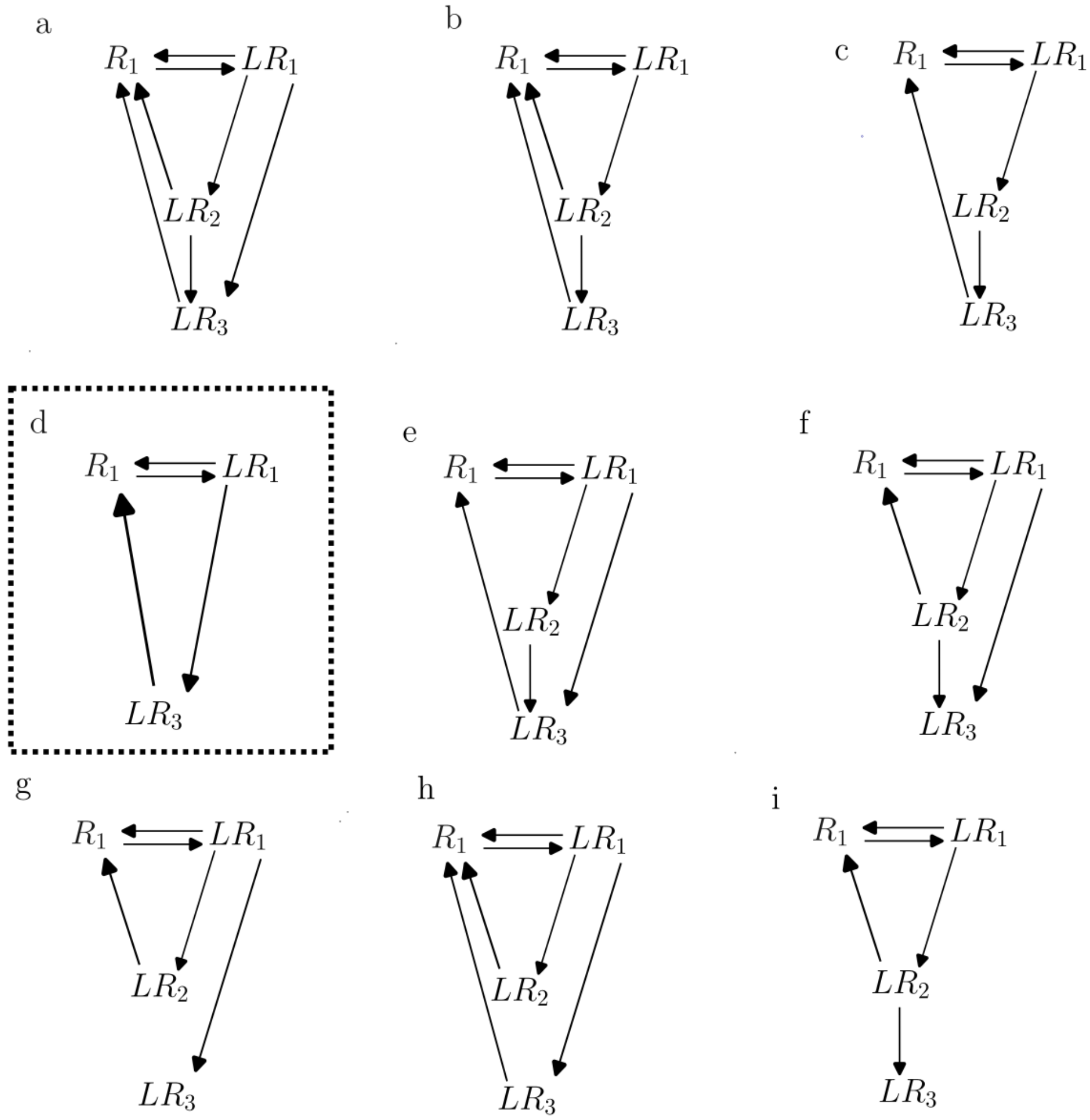

Fig C. Trafficking models used for model selection. R_1 represents the amount of receptors at the plasma membrane and LR_1 (respectively LR_2 and LR_3) the amount of ligand-receptor complex at plasma membrane (respectively in two types of intracellular compartments). Model *d* corresponds to model with only two compartments (Q1), while all other models integrate three compartments. Then, different assumptions about trafficking are tested (a, b, c, e, f, g, h and i) (Q6): internalization of LR_1, recycling of LR_2 and LR_3, trafficking between compartments (compartment 2 to compartment 3). All these models are embedded inside the complete three compartments model (Model 3 (Fig 5))). When no arrow is shown, the corresponding parameter value is set to zero.

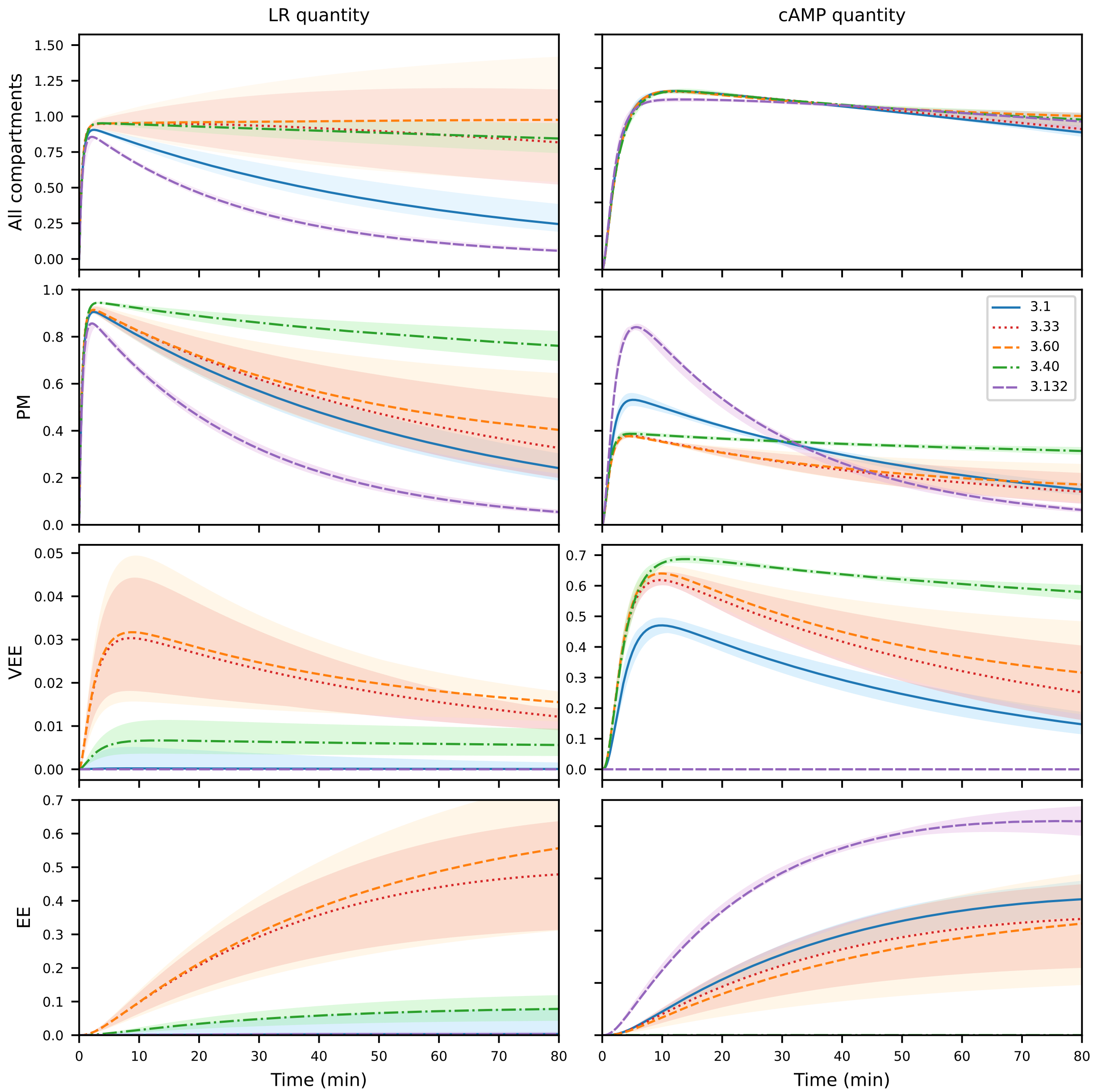

Fig D. Prediction of cAMP levels and LR complex quantities in each compartment for five models. LR complex levels (first column) and cAMP quantities (second column) are predicted in each compartment for a single concentration of FSH (10 nM) for trafficking model c, using five different models (according to legend). The solid line (blue) represents the best model with the favorite cluster (3.1); the dotted line (red) corresponds to trafficking model c without desensitization at the plasma membrane but with desensitization from the compartment 3 (LR_3) (3.33), the dashed line (orange) corresponds to trafficking model c without desensitization at the plasma membrane (3.60), the dash-dot line (green) represents trafficking model c with cAMP production restricted to the first two compartments (3.40), and the purple represents trafficking model c with cAMP production restricted to the two compartments LR_1 and LR_3 (3.132). Predictions are shown with 95% profile-based confidence intervals. The parameters used for these predictions correspond to the best estimates obtained in each parameter estimation problem (S4 Table).

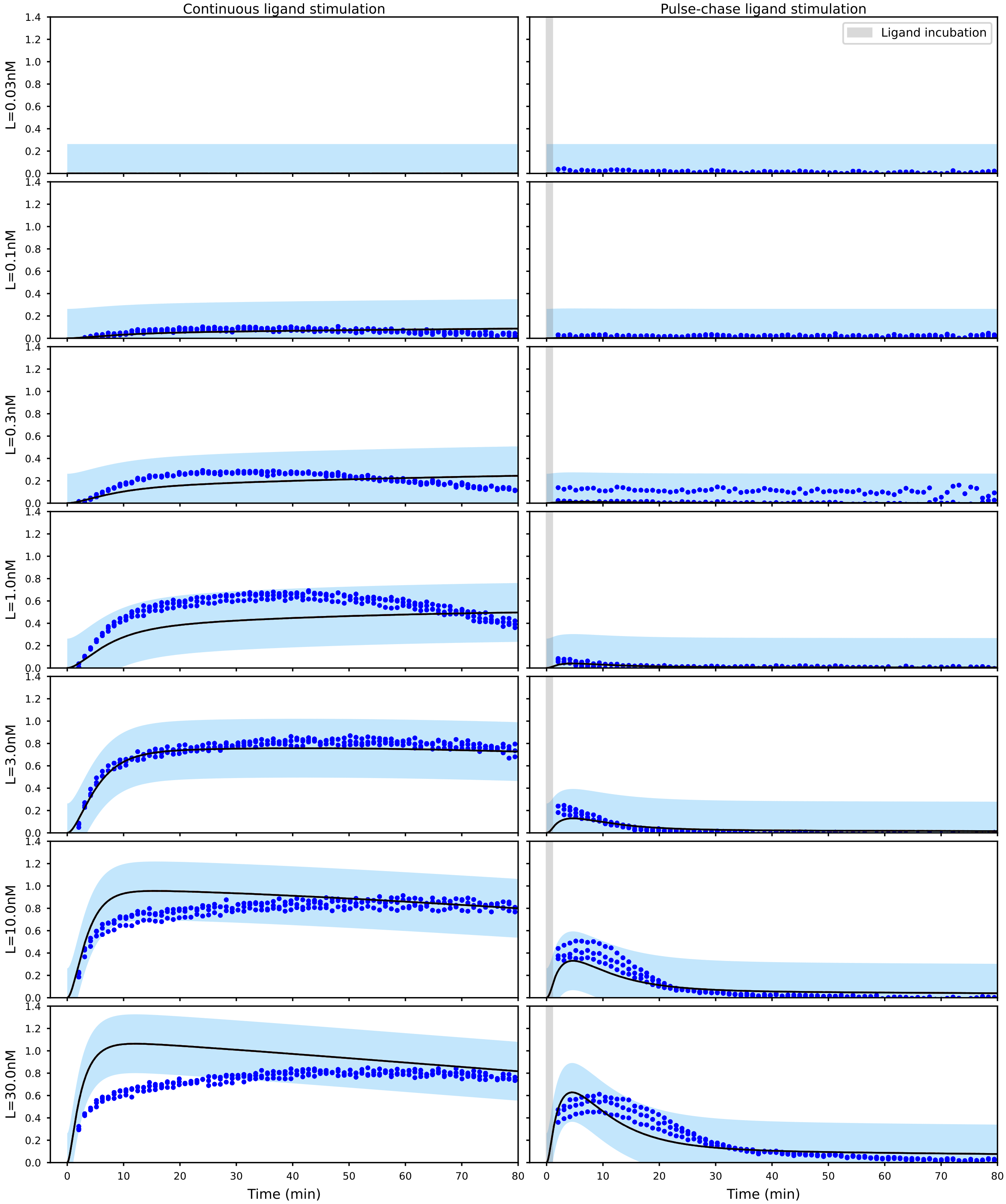

Fig E. Prediction of cAMP quantity with validation data for the five best models. Continuous ligand stimulation condition is shown in the first column and pulse-chase ligand stimulation condition in the second column. cAMP quantity produced for 7 doses of ligand is showed. The parameters used for these predictions correspond to the best estimates obtained for the five best models which were selected according to the AIC criterion (S4 Table) with their statistical error (shaded areas).

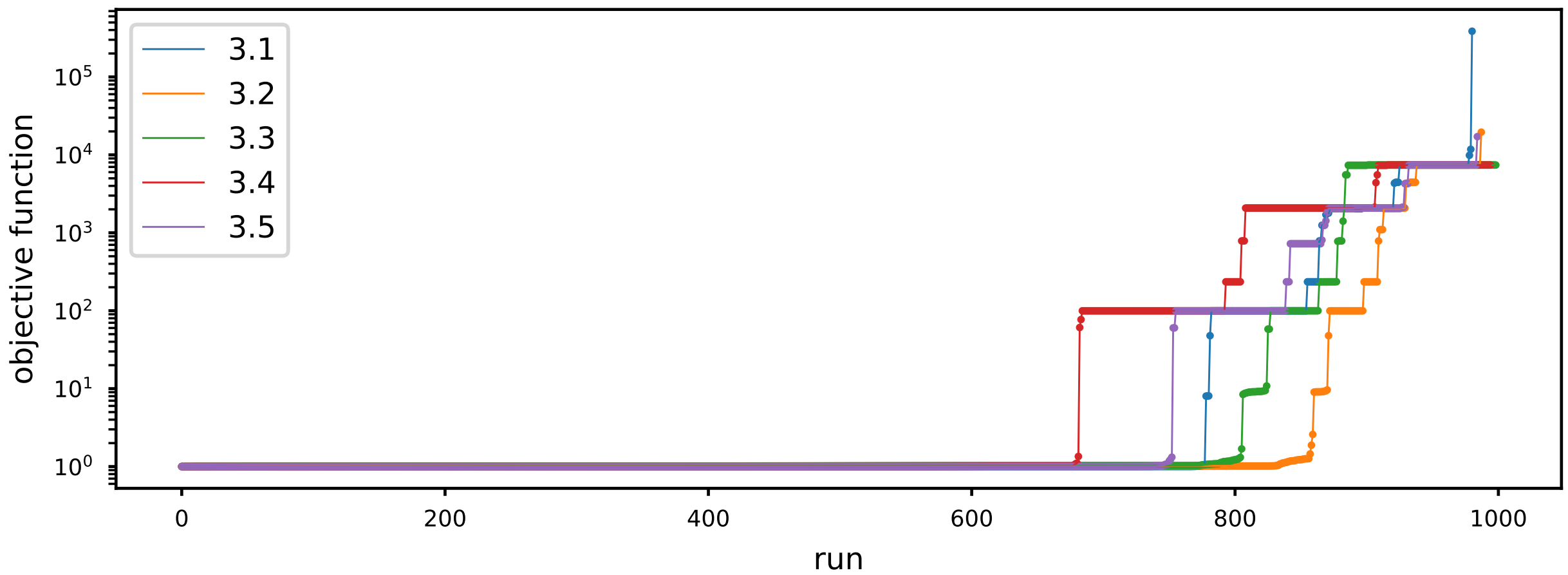

Fig F. Convergence algorithm for the five best models. To resolve the parameter estimation, the algorithm employs deterministic gradient descent with multi-start optimization, using 1000 runs with random initial parameter values to ensure a satisfactory fit with the optimizer Fides. Several runs reached to the same optimum for the five best models (3.1, 3.2, 3.3, 3.4 and 3.5) leading to algorithm convergence.

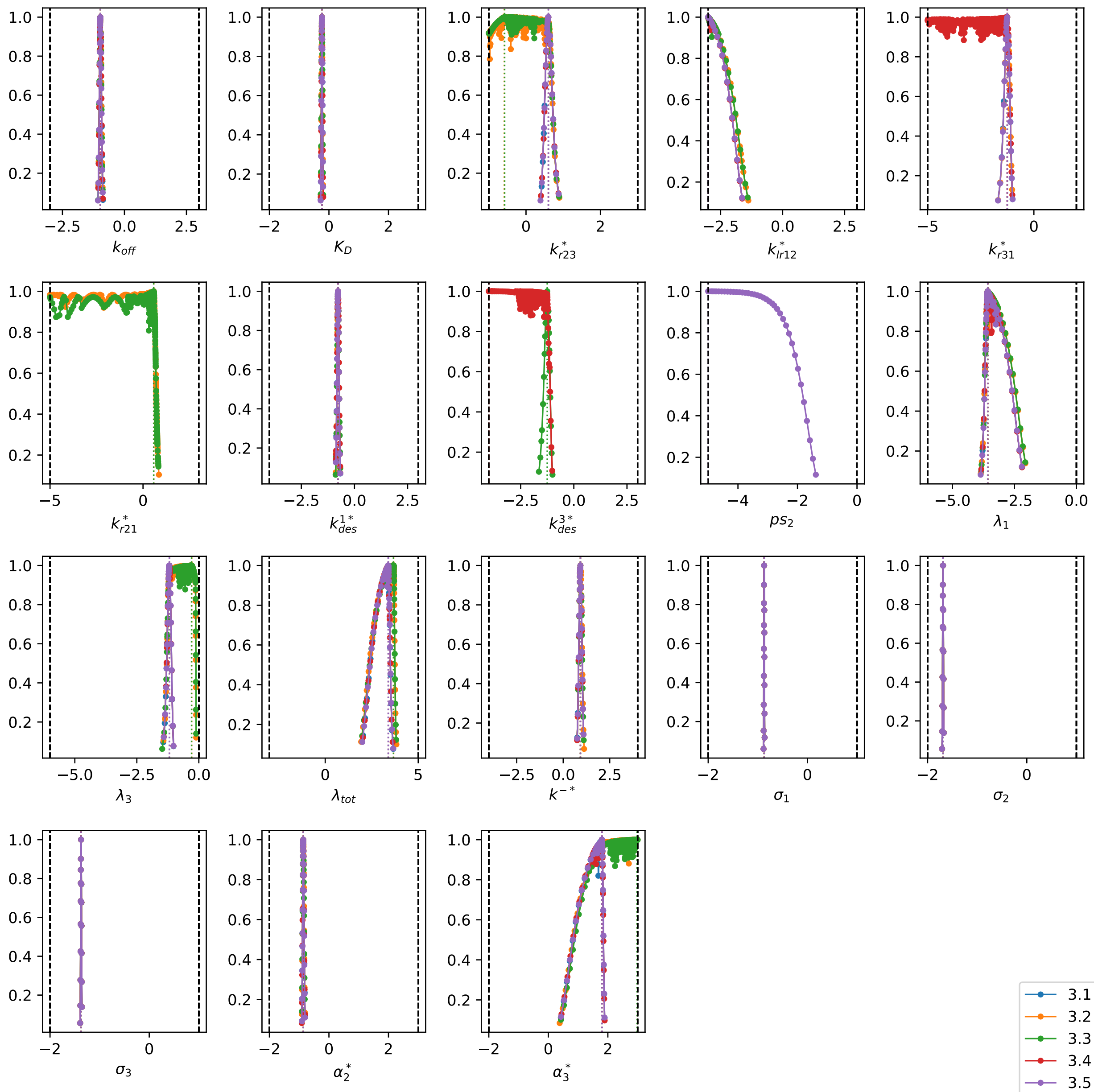

Fig G. Profile likelihood for all parameters for the five best models. The plot shows the results of the Profile Likelihood Estimate (PLE) method, used to evaluate practical identifiability relative to available biological data, for all estimated parameters of the five best models (3.1, 3.2, 3.3, 3.4 and 3.5).

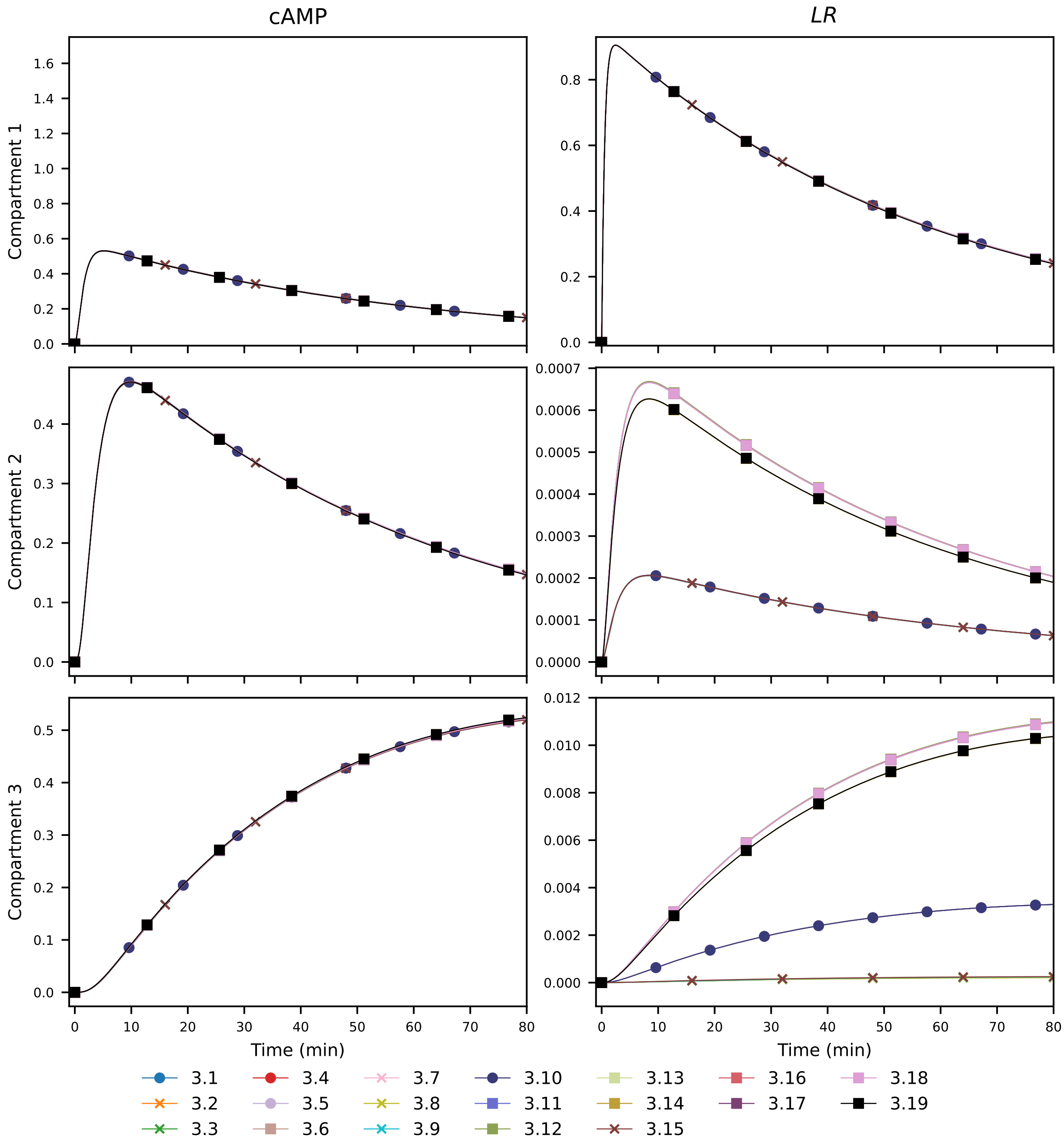

Fig H. Prediction cAMP levels and LR complexes in each compartment for the 19 best models. Single predictions for all best models were simulated in response to a single FSH dose (10 nM). Parameters correspond to the best estimates obtained during model selection (S4 Table). Rounds show cluster 1, crosses the cluster 2 and squares a third cluster.

Table A. Table of the initial quantities, the variables and the parameters for models 1,2,3. This table describes the initial quantities, variables and parameters for these models, including their notation, units and descriptions.

	Terms	Unit	Description
Initial quantities	R_0	mol	Initial quantity of receptors
	L	mol	Initial quantity of ligand
Variables	R_1	mol	Amount of receptors at the PM
	LR_i, $i = 1, 2, 3$	mol	Amount of complex ligand-receptors in compartment i
	$cAMP$	mol	Amount of cAMP
Parameters			
All models	k_{off}	s^{-1}	Speed of dissociation of the complex ligand-receptor
	k_{on}	$mol^{-1}.s^{-1}$	Speed of association to form the complex ligand-receptor
	$K_D = \frac{k_{off}}{k_{on}}$	mol	Constant of dissociation of the complex ligand-receptor
	k_i^+, $i = 1, 2, 3$	s^{-1}	Production rate of cAMP in compartment i
	k^-	s^{-1}	Degradation rate of cAMP
Model 1 (Fig 1)	k_{ixy}	s^{-1}	Internalization rate from x to y
	k_{rxy}	s^{-1}	Recycling rate from x to y
Model 2 (Fig 3)	k_{ixy}	s^{-1}	Internalization rate from x to y
	k_{rxy}	s^{-1}	Recycling rate from x to y
	p_{23}	s^{-1}	Proportion receptors from 2 to 3
Model 3 (Fig 5)	k_{int}	s^{-1}	Internalization rate
	k_{ixy}	s^{-1}	Internalization rate from x to y
	k_{rxy}	s^{-1}	Recycling rate from x to y
	k_{des}^i, $i = 1, 3$	s^{-1}	Desensitization rate from the compartment i
	ps_2	-	Internalization inhibition effect

Table B. Result of model selection based on AIC criterion. 200 models were constructed by combining several hypotheses (8 x 3 x 2 x 2 x 2 + 2 x 2 x 2 = 200): the number of compartments (Q1)); whether the ligand-receptor complex is functionally active in two or three compartments (1 means active in the three compartments, 2 in LR_1 and LR_2 and 3 in LR_1 and LR_3) (Q2); if the receptor internalization inhibition drug is partially or fully efficient (Q3); if the receptor is desensitized at plasma membrane (Q4) or at early endosomes (Q5) and the different trafficking hypotheses (Q6). Crosses mean that the parameter was estimated, whereas when the box is empty, the corresponding parameter was fixed to 0.

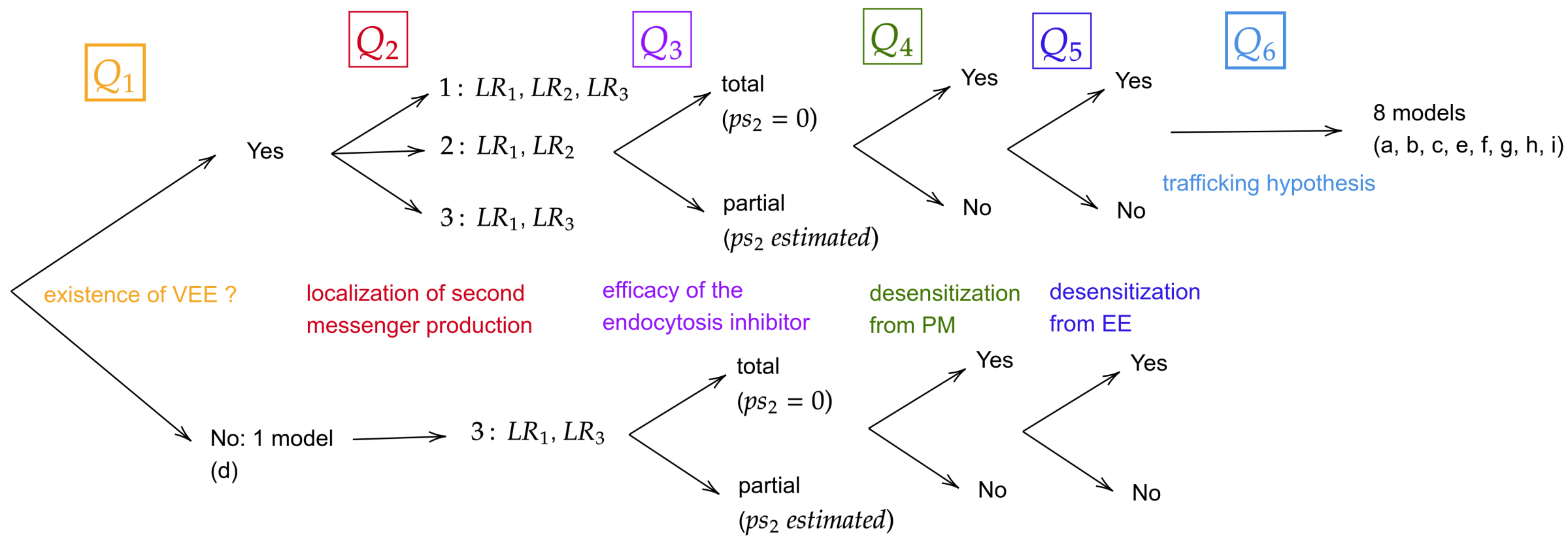

Model	Q1	Q2	Q3	Q4	Q5	Q6	AIC	Δ_{AIC}
3.1	3	1		x		c	-16629.88	0.00
3.2	3	1		x		b	-16627.91	1.97
3.3	3	1		x	x	i	-16627.91	1.97
3.4	3	1		x	x	c	-16627.88	2.00
3.5	3	1	x	x		c	-16627.88	2.00
3.6	3	1		x		e	-16627.02	2.86
3.7	3	1		x	x	b	-16625.91	3.97
3.8	3	1	x	x		b	-16625.91	3.97
3.9	3	1	x	x	x	i	-16625.91	3.97
3.10	3	1	x	x	x	c	-16625.88	4.00
3.11	3	1		x		a	-16625.02	4.86
3.12	3	1		x	x	e	-16625.02	4.86
3.13	3	1	x	x		e	-16625.02	4.86
3.14	3	1		x	x	f	-16624.95	4.93
3.15	3	1	x	x	x	b	-16623.91	5.97
3.16	3	1		x	x	a	-16623.02	6.86
3.17	3	1	x	x		a	-16623.02	6.86
3.18	3	1	x	x	x	e	-16623.02	6.86
3.19	3	1	x	x	x	f	-16622.95	6.93
3.20	3	1	x	x	x	a	-16621.02	8.86
3.21	3	1		x		i	-16615.85	14.03
3.22	3	1	x	x		i	-16613.85	16.03
3.23	3	1		x		f	-16613.59	16.29
3.24	3	1		x	x	g	-16613.29	16.59
3.25	3	1		x		h	-16613.29	16.59
3.26	3	1		x		g	-16612.28	17.60
3.27	3	1	x	x		f	-16611.59	18.29
3.28	3	1		x	x	h	-16611.29	18.59
3.29	3	1	x	x		h	-16611.29	18.59
3.30	3	1	x	x	x	g	-16611.29	18.59
3.31	3	1	x	x		g	-16610.27	19.61
3.32	3	1	x	x	x	h	-16609.29	20.59
3.33	3	1			x	c	-16541.02	88.86

Model	Q1	Q2	Q3	Q4	Q5	Q6	AIC	Δ_{AIC}
3.34	3	1			x	i	-16540.45	89.43
3.35	3	1			x	b	-16539.02	90.86
3.36	3	1	x		x	c	-16539.01	90.87
3.37	3	1			x	e	-16539.00	90.88
3.38	3	1	x		x	i	-16538.45	91.43
3.39	3	1			x	f	-16538.44	91.44
3.40	3	2		x		c	-16538.23	91.65
3.41	3	1	x		x	b	-16537.01	92.87
3.42	3	1			x	a	-16537.00	92.88
3.43	3	1	x		x	e	-16537.00	92.88
3.44	3	1	x		x	f	-16536.44	93.44
3.45	3	2		x		b	-16536.23	93.65
3.46	3	2	x	x		c	-16536.23	93.65
3.47	3	2		x	x	c	-16536.22	93.66
3.48	3	2		x		e	-16536.17	93.71
3.49	3	1	x		x	a	-16535.00	94.88
3.50	3	2	x	x		b	-16534.23	95.65
3.51	3	2		x	x	b	-16534.22	95.66
3.52	3	2	x	x	x	c	-16534.22	95.66
3.53	3	2		x		a	-16534.17	95.71
3.54	3	2	x	x		e	-16534.16	95.71
3.55	3	2		x	x	e	-16534.16	95.72
3.56	3	2	x	x	x	b	-16532.22	97.66
3.57	3	2	x	x		a	-16532.16	97.71
3.58	3	2		x	x	a	-16532.16	97.72
3.59	3	2	x	x	x	e	-16532.16	97.72
3.60	3	1				c	-16532.15	97.73
3.61	3	2	x	x	x	a	-16530.16	99.72
3.62	3	1				b	-16530.15	99.73
3.63	3	1	x			c	-16530.15	99.73
3.64	3	1				e	-16530.15	99.73
3.65	3	1	x			b	-16528.15	101.73
3.66	3	1				a	-16528.15	101.73
3.67	3	1	x			e	-16528.14	101.74
3.68	3	1	x			a	-16526.14	103.74
3.69	3	2			x	c	-16525.95	103.93
3.70	3	1				i	-16525.01	104.87
3.71	3	2			x	b	-16523.95	105.93
3.72	3	2	x		x	c	-16523.95	105.93
3.73	3	2			x	e	-16523.92	105.96
3.74	3	2				c	-16523.89	105.99
3.75	3	1				f	-16523.01	106.87
3.76	3	1	x			i	-16523.01	106.87
3.77	3	2	x		x	b	-16521.95	107.93
3.78	3	2			x	a	-16521.92	107.96

Model	Q1	Q2	Q3	Q4	Q5	Q6	AIC	Δ_{AIC}
3.79	3	2	x		x	e	-16521.92	107.96
3.80	3	2				b	-16521.89	107.99
3.81	3	2	x			c	-16521.89	107.99
3.82	3	2				e	-16521.87	108.01
3.83	3	1	x			f	-16521.01	108.87
3.84	3	2	x		x	a	-16519.92	109.96
3.85	3	2	x			b	-16519.89	109.99
3.86	3	2				a	-16519.87	110.01
3.87	3	2	x			e	-16519.87	110.01
3.88	3	1			x	g	-16519.85	110.03
3.89	3	1			x	h	-16518.17	111.71
3.90	3	2	x			a	-16517.87	112.01
3.91	3	1	x		x	g	-16517.84	112.04
3.92	3	1				h	-16517.56	112.31
3.93	3	1				g	-16516.64	113.24
3.94	3	1	x		x	h	-16516.17	113.71
3.95	3	2		x	x	i	-16515.75	114.13
3.96	3	1	x			h	-16515.56	114.32
3.97	3	1	x			g	-16514.63	115.25
3.98	3	2	x	x	x	i	-16513.75	116.13
3.99	3	2		x		h	-16512.39	117.49
3.100	3	2		x	x	f	-16512.33	117.55
3.101	3	2	x	x		h	-16510.39	119.49
3.102	3	2		x	x	h	-16510.38	119.50
3.103	3	2	x	x	x	f	-16510.33	119.55
3.104	3	2				h	-16508.98	120.90
3.105	3	2	x	x	x	h	-16508.38	121.50
3.106	3	2			x	h	-16506.99	122.89
3.107	3	2	x			h	-16506.98	122.90
3.108	3	2	x		x	h	-16504.99	124.89
3.109	3	2		x	x	g	-16495.93	133.95
3.110	3	2	x	x	x	g	-16493.93	135.95
3.111	3	2			x	i	-16485.01	144.87
3.112	3	2	x		x	i	-16483.01	146.87
3.113	3	2			x	f	-16482.97	146.91
3.114	3	2	x		x	f	-16480.97	148.91
3.115	3	2			x	g	-16466.44	163.44
3.116	3	2	x		x	g	-16464.44	165.44
3.117	3	2		x		g	-16440.96	188.92
3.118	3	2	x	x		g	-16438.96	190.92
3.119	3	2		x		f	-16432.89	196.99
3.120	3	2	x	x		f	-16430.89	198.99
3.121	3	2		x		i	-16412.80	217.08
3.122	3	2	x	x		i	-16410.80	219.08
3.123	3	2				g	-16404.06	225.82

Model	Q1	Q2	Q3	Q4	Q5	Q6	AIC	Δ_{AIC}
3.124	3	2	x			g	-16402.06	227.82
3.125	3	2				f	-16401.62	228.26
3.126	3	2	x			f	-16399.62	230.26
3.127	3	2	x			i	-16389.08	240.80
3.128	3	2				i	-16380.23	249.65
3.129	2	3		x		d	-16167.56	462.32
3.130	2	3		x	x	d	-16165.56	464.32
3.131	2	3	x	x		d	-16165.55	464.33
3.132	3	3		x		c	-16163.90	465.98
3.133	2	3	x	x	x	d	-16163.55	466.33
3.134	3	3		x	x	g	-16163.19	466.69
3.135	3	3		x		h	-16163.18	466.70
3.136	3	3		x	x	i	-16163.04	466.84
3.137	3	3		x		b	-16162.93	466.95
3.138	3	3		x	x	c	-16161.99	467.89
3.139	3	3	x	x		c	-16161.90	467.98
3.140	3	3		x		e	-16161.74	468.14
3.141	3	3		x	x	h	-16161.19	468.69
3.142	3	3		x	x	f	-16161.19	468.69
3.143	3	3		x		a	-16161.18	468.70
3.144	3	3	x	x	x	g	-16161.18	468.70
3.145	3	3	x	x		h	-16161.17	468.71
3.146	3	3	x	x	x	i	-16160.97	468.91
3.147	3	3	x	x		b	-16160.93	468.95
3.148	3	3		x	x	b	-16160.92	468.96
3.149	3	3	x	x	x	c	-16159.98	469.90
3.150	3	3		x	x	e	-16159.83	470.05
3.151	3	3	x	x		e	-16159.73	470.15
3.152	3	3		x	x	a	-16159.19	470.69
3.153	3	3	x	x	x	h	-16159.18	470.70
3.154	3	3	x	x	x	f	-16159.18	470.70
3.155	3	3	x	x		a	-16159.17	470.71
3.156	3	3	x	x	x	b	-16158.92	470.96
3.157	3	3	x	x	x	e	-16157.82	472.06
3.158	3	3		x		g	-16157.25	472.63
3.159	3	3	x	x	x	a	-16157.18	472.70
3.160	3	3		x		i	-16157.06	472.82
3.161	3	3		x		f	-16155.25	474.63
3.162	3	3	x	x		g	-16155.24	474.64
3.163	3	3	x	x		i	-16154.99	474.89
3.164	3	3	x	x		f	-16153.24	476.64
3.165	3	3			x	e	-15722.75	907.13
3.166	3	3			x	f	-15722.74	907.14
3.167	3	3			x	a	-15720.74	909.14
3.168	3	3	x		x	e	-15720.73	909.15

Model	Q1	Q2	Q3	Q4	Q5	Q6	AIC	Δ_{AIC}
3.169	3	3	x		x	f	-15720.73	909.15
3.170	3	3	x		x	a	-15718.73	911.15
3.171	3	3				e	-15673.45	956.43
3.172	3	3				a	-15671.44	958.44
3.173	3	3	x			e	-15671.44	958.44
3.174	3	3	x			a	-15669.43	960.45
3.175	3	3			x	g	-15564.16	1065.72
3.176	3	3				h	-15563.76	1066.12
3.177	3	3			x	h	-15562.16	1067.72
3.178	3	3	x		x	g	-15562.16	1067.72
3.179	3	3	x			h	-15561.75	1068.13
3.180	3	3	x		x	h	-15560.16	1069.72
3.181	2	3			x	d	-15516.27	1113.61
3.182	3	3			x	c	-15514.50	1115.38
3.183	3	3			x	i	-15514.45	1115.43
3.184	2	3	x		x	d	-15514.27	1115.61
3.185	3	3			x	b	-15512.50	1117.38
3.186	3	3	x		x	c	-15512.50	1117.38
3.187	3	3	x		x	i	-15512.45	1117.43
3.188	3	3	x		x	b	-15510.50	1119.38
3.189	2	3				d	-15064.93	1564.95
3.190	3	3				g	-15063.02	1566.86
3.191	3	3				i	-15062.99	1566.89
3.192	2	3	x			d	-15062.91	1566.97
3.193	3	3				c	-15062.88	1567.00
3.194	3	3				f	-15061.04	1568.84
3.195	3	3	x			g	-15060.99	1568.89
3.196	3	3	x			i	-15060.96	1568.92
3.197	3	3				b	-15060.88	1569.00
3.198	3	3	x			c	-15060.85	1569.03
3.199	3	3	x			f	-15059.01	1570.87
3.200	3	3	x			b	-15058.85	1571.03

Table C. Table of the value of the best estimated parameters for the 19 best models. The parameters were estimated using a deterministic gradient descent algorithm with multi-start optimization, and the best estimate of each parameter for the 19 best models is presented in this table.

Param.	3.1	3.2	3.3	3.4	3.5	3.6	3.7	3.8	3.9	3.10	Unit
k_{on}	0.184	0.184	0.184	0.184	0.184	0.184	0.184	0.184	0.184	0.184	$nmol^{-1}.s^{-1}$
k_{off}	0.107	0.107	0.107	0.107	0.107	0.107	0.107	0.107	0.107	0.107	s^{-1}
K_D	0.583	0.582	0.582	0.583	0.583	0.583	0.582	0.582	0.582	0.583	$nmol$
k_{r21}	0	0.402	0.400	0	0	0	0.399	0.402	0.398	0	s^{-1}
k_{r31}	0.006	0.006	0	0.006	0.006	0.006	0.006	0.006	0	0.006	s^{-1}
k_{i12}	1.1e-04	1.1e-04	1.1e-04	1.1e-04	1.1e-04	3.4e-04	1.1e-04	1.1e-04	1.1e-04	1.1e-04	s^{-1}
k_{i13}	0	0	0	0	0	1.1e-05	0	0	0	0	s^{-1}
k_{r23}	0.428	0.028	0.029	0.428	0.428	0.426	0.030	0.027	0.031	0.428	s^{-1}
k^1_{des}	0.018	0.018	0.018	0.018	0.018	0.018	0.018	0.018	0.018	0.018	s^{-1}
k^3_{des}	0	0	0.006	1.1e-05	0	0	9.2e-05	0	0.006	1.1e-05	s^{-1}
k^+_1	0.552	0.551	0.552	0.552	0.552	0.553	0.552	0.552	0.551	0.552	s^{-1}
k^+_2	2076	2080	2077	2075	2076	648	2079	2077	2080	2076	s^{-1}
k^+_3	143	2204	2153	143	143	43.414	2021	2248	1975	143	s^{-1}
k^-	0.905	0.904	0.904	0.905	0.905	0.909	0.904	0.904	0.904	0.905	s^{-1}
ps_2	0	0	0	0	1.0e-05	0	0	1.0e-05	1.5e-05	1.0e-05	-
σ_1	0.134	0.134	0.134	0.134	0.134	0.134	0.134	0.134	0.134	0.134	-
σ_2	0.020	0.020	0.020	0.020	0.020	0.020	0.020	0.020	0.020	0.020	-
σ_3	0.042	0.042	0.042	0.042	0.042	0.042	0.042	0.042	0.042	0.042	-
α^*_2	0.137	0.137	0.137	0.137	0.137	0.137	0.137	0.137	0.137	0.137	-
α^*_3	63.642	981	958	63.640	63.641	19.151	899	1000	879	63.641	-

Param.	3.11	3.12	3.13	3.14	3.15	3.16	3.17	3.18	3.19	Unit
k_{on}	0.184	0.184	0.184	0.184	0.184	0.184	0.184	0.184	0.184	$nmol^{-1}.s^{-1}$
k_{off}	0.107	0.107	0.107	0.107	0.107	0.107	0.107	0.107	0.107	s^{-1}
K_D	0.583	0.583	0.583	0.584	0.582	0.583	0.583	0.583	0.584	$nmol$
k_{r21}	1.3e-06	0	0	4.8e-06	0.396	1.8e-05	1.2e-06	0	1.2e-06	s^{-1}
k_{r31}	0.006	0.006	0.006	0	0.006	0.006	0.006	0.006	0	s^{-1}
k_{i12}	3.4e-04	3.5e-04	3.4e-04	3.2e-04	1.1e-04	3.4e-04	3.4e-04	3.4e-04	3.3e-04	s^{-1}
k_{i13}	1.1e-05	1.1e-05	1.1e-05	1.1e-05	0	1.1e-05	1.1e-05	1.1e-05	1.1e-05	s^{-1}
k_{r23}	0.426	0.426	0.426	0.427	0.033	0.426	0.426	0.426	0.427	s^{-1}
k^1_{des}	0.018	0.018	0.018	0.018	0.018	0.018	0.018	0.018	0.018	s^{-1}
k^3_{des}	0	1.1e-05	0	0.006	1.6e-05	1.1e-05	0	1.1e-05	0.006	s^{-1}
k^+_1	0.553	0.553	0.553	0.552	0.552	0.553	0.553	0.553	0.552	s^{-1}
k^+_2	648	646	648	687	2078	648	648	648	686	s^{-1}
k^+_3	43.405	43.298	43.419	46.019	1869	43.443	43.438	43.446	45.961	s^{-1}
k^-	0.909	0.909	0.909	0.907	0.904	0.909	0.909	0.909	0.907	s^{-1}
ps_2	0	0	1.0e-05	0	1.0e-05	0	1.0e-05	1.0e-05	1.0e-05	-
σ_1	0.134	0.134	0.134	0.134	0.134	0.134	0.134	0.134	0.134	-
σ_2	0.020	0.020	0.020	0.020	0.020	0.020	0.020	0.020	0.020	-
σ_3	0.042	0.042	0.042	0.042	0.042	0.042	0.042	0.042	0.042	-
α^*_2	0.137	0.137	0.137	0.137	0.137	0.137	0.137	0.137	0.137	-
α^*_3	19.146	19.099	19.154	20.269	831	19.164	19.161	19.165	20.242	-

Table D. Table of the value of best parameters for five different models. The parameters were estimated using a deterministic gradient descent algorithm with multi-start optimization, and the best estimate of each parameter for models 3.1, 3.33, 3.60, 3.40 and 3.124 is presented in this table.

Param.	1	33	60	40	132	Unit
k_{on}	0.184	0.204	0.198	0.182	0.175	$nmol^{-1}.s^{-1}$
k_{off}	0.107	0.116	0.109	0.080	0.141	s^{-1}
K_D	0.583	0.570	0.551	0.442	0.803	$nmol$
k_{r21}	0	0	0	0	0	s^{-1}
k_{r31}	0.006	0.002	0.007	0.023	0.003	s^{-1}
k_{i12}	1.1e-04	0.016	0.016	0.003	1.4e-04	s^{-1}
k_{i13}	0	0	0	0	0	s^{-1}
k_{r23}	0.428	0.432	0.418	0.365	141	s^{-1}
k^1_{des}	0.018	0	0	0.002	0.038	s^{-1}
k^3_{des}	0	0.007	0	0	0	s^{-1}
k^+_1	0.552	0.508	0.511	0.623	0.652	s^{-1}
k^+_2	2076	24.357	24.533	156	0	s^{-1}
k^+_3	143	1.109	0.934	0	171	s^{-1}
k^-	0.905	1.191	1.212	1.513	0.597	s^{-1}
ps_2	0	0	0	0	0	-
σ_1	0.134	0.136	0.136	0.136	0.140	-
σ_2	0.020	0.020	0.020	0.021	0.021	-
σ_3	0.042	0.042	0.042	0.042	0.044	-
α^*_2	0.137	0.158	0.169	0.525	0.091	-
α^*_3	63.642	0.437	0.406	2.680	61.605	-

Table E. Summary of results for the plausible models selected based on the AIC criterion. The table below reports the outcomes of the selection procedure for models 3.6 to 3.19, including the answers from Q1 to Q6 and the corresponding statistics for each model (log-likelihood, AIC, number of estimated parameters, convergence status), revealing the existence of two distinct model clusters. Convergence was calculated as the number of runs with a negative log-likelihood value below the minimum negative log-likelihood value plus 0.1.

	3.6	3.7	3.8	3.9	3.10	3.11	3.12
Q1 - Existence of VEE	yes	yes	yes	yes	yes	yes	yes
Q2 - Localization of second messenger production	$LR_{1,2,3}$	$LR_{1,2,3}$	$LR_{1,2,3}$	$LR_{1,2,3}$	$LR_{1,2,3}$	$LR_{1,2,3}$	$LR_{1,2,3}$
Q3 - Efficacy of the inhibitor endocytosis	total	total	partial	partial	partial	total	total
Q4 - Desensitization from PM	yes	yes	yes	yes	yes	yes	yes
Q5 - Desensitization from EE	no	yes	no	yes	yes	no	yes
Q6 - Trafficking hypotheses	e	b	b	i	c	a	e
Internalization from PM to EE	yes	no	no	no	no	yes	yes
Recycling from VEE	no	yes	yes	no	no	yes	no
Internalization from PM to VEE	yes	yes	yes	yes	yes	yes	yes
Recycling from EE	yes	yes	yes	no	yes	yes	yes
Negative log-likelihood	-8329.51	-8329.96	-8329.96	-8328.95	-8327.94	-8329.51	-8329.51
AIC	-16627.02	-16625.91	-16625.91	-16625.91	-16625.88	-16625.02	-16625.02
Δ_{AIC}	2.86	3.97	3.97	3.97	4.00	4.86	4.86
Number of estimated parameters	16		17	17	17	17	17
Convergence (%)	78.9	77.8	77.7	76.2	62.8	77.6	64.4
Cluster	3	2	2	2	1	3	3

	3.13	3.14	3.15	3.16	3.17	3.18	3.19
Q1 - Existence of VEE	yes	yes	yes	yes	yes	yes	yes
Q2 - Localization of second messenger production	$LR_{1,2,3}$	$LR_{1,2,3}$	$LR_{1,2,3}$	$LR_{1,2,3}$	$LR_{1,2,3}$	$LR_{1,2,3}$	$LR_{1,2,3}$
Q3 - Efficacy of the inhibitor endocytosis	partial	total	partial	total	partial	partial	partial
Q4 - Desensitization from PM	yes	yes	yes	yes	yes	yes	yes
Q5 - Desensitization from EE	no	yes	yes	yes	no	yes	yes
Q6 - Trafficking hypothesis	e	f	b	a	a	e	f
Internalization from PM to EE	yes	yes	no	yes	yes	yes	yes
Recycling from VEE	no	yes	yes	yes	yes	no	yes
Internalization from PM to VEE	yes	yes	yes	yes	yes	yes	yes
Recycling from EE	yes	no	yes	yes	yes	yes	no
Negative log-likelihood	-8329.51	-8329.47	-8329.96	-8329.51	-8329.51	-8329.51	-8328.47
AIC	-16625.02	-16624.95	-16623.91	-16623.02	-16623.02	-16623.02	-16622.95
Δ_{AIC}	4.86	4.93	5.97	6.86	6.86	6.86	6.93
Number of estimated parameters	17	17	18	18	18	18	18
Convergence (%)	64.4	71.8	77.9	68.8	65.0	62.5	66.3
Cluster	3	3	2	3	3	3	3

Table F. Averaged outputs for the models 3.6 to 3.19. The table presents the proportion of internalized receptors ($LR_2 + LR_3$) relative to total receptors, and the proportion of receptors in VEE or EE relative to internalized receptors ($LR_2 + LR_3$), but also endocytosis and recycling time and probability for a 5 min incubation with 10 nM of ligand. Values in brackets indicate uncertainty.

	3.6	3.7	3.8	3.9	3.10
LR_{intra} (%)	0.16 [0.04, 1.40]	0.02 [0.01, 1.23]	0.02 [0.01, 1.12]	0.02 [0.01, 1.14]	0.05 [0.04, 1.17]
LR_2 (%)	42.76 [26.74, 50.74]	91.69 [26.66, 97.20]	92.47 [30.23, 97.22]	91.50 [30.11, 97.20]	43.95 [27.63, 52.35]
LR_3 (%)	57.24 [49.26, 73.26]	8.31 [2.80, 73.34]	7.53 [2.78, 69.77]	8.50 [2.80, 69.89]	56.05 [47.65, 72.37]
Time endocytosis (s)	58.44 [50.06, 71.74]	58.41 [48.24, 70.11]	58.42 [48.52, 69.35]	58.42 [47.29, 69.25]	58.62 [49.73, 70.38]
Probability endocytosis	0.02 [0.01, 0.17]	0.01 [0.00, 0.18]	0.01 [0.00, 0.19]	0.01 [0.00, 0.20]	0.01 [0.00, 0.14]
Time recycling (s)	171.92 [105.59, 459.72]	13.96 [2.24, 196.46]	12.92 [4.36, 186.42]	2.33 [1.33, 2.64]	168.88 [107.35, 236.16]
Probability recycling	1.00 [1.00, 1.00]	1.00 [0.30, 1.00]	1.00 [1.00, 1.00]	0.93 [0.00, 0.98]	1.00 [0.00, 1.00]

	3.11	3.12	3.13	3.14	3.15
LR_{intra} (%)	0.16 [0.03, 1.26]	0.16 [0.04, 1.42]	0.16 [0.04, 1.42]	0.15 [0.03, 1.35]	0.02 [0.01, 1.13]
LR_2 (%)	42.77 [27.13, 95.71]	42.78 [27.11, 50.91]	42.78 [27.11, 50.91]	42.61 [27.70, 95.80]	91.08 [29.77, 97.24]
LR_3 (%)	57.23 [4.29, 72.87]	57.22 [49.09, 72.89]	57.22 [49.09, 72.89]	57.39 [4.20, 72.30]	8.92 [2.76, 70.23]
Time endocytosis (s)	58.43 [48.74, 71.40]	58.43 [50.09, 71.76]	58.44 [50.05, 71.82]	58.36 [49.17, 71.80]	58.41 [48.09, 70.11]
Probability endocytosis	0.02 [0.01, 0.19]	0.02 [0.01, 0.17]	0.02 [0.01, 0.17]	0.02 [0.01, 0.19]	0.01 [0.00, 0.20]
Time recycling (s)	171.91 [7.95, 458.62]	171.91 [104.64, 236.58]	171.94 [105.57, 457.93]	7.78 [2.60, 29.89]	15.06 [2.21, 190.04]
Probability recycling	1.00 [1.00, 1.00]	1.00 [0.00, 1.00]	1.00 [1.00, 1.00]	0.00 [0.00, 0.97]	1.00 [0.10, 1.00]

	3.16	3.17	3.18	3.19
LR_{intra} (%)	0.16 [0.03, 1.28]	0.16 [0.03, 1.22]	0.16 [0.04, 1.43]	0.15 [0.03, 1.39]
LR_2 (%)	42.76 [25.57, 95.65]	42.77 [25.86, 95.66]	42.77 [27.14, 50.83]	42.61 [25.65, 95.78]
LR_3 (%)	57.24 [4.35, 74.43]	57.23 [4.34, 74.14]	57.23 [49.17, 72.86]	57.39 [4.22, 74.35]
Time endocytosis (s)	58.44 [49.39, 71.76]	58.43 [48.96, 71.48]	58.43 [50.07, 71.88]	58.36 [48.67, 71.69]
Probability endocytosis	0.02 [0.01, 0.20]	0.02 [0.01, 0.19]	0.02 [0.01, 0.17]	0.02 [0.01, 0.20]
Time recycling (s)	171.92 [8.10, 239.77]	171.92 [8.02, 458.61]	171.92 [106.55, 237.48]	7.77 [2.58, 32.24]
Probability recycling	1.00 [0.00, 1.00]	1.00 [1.00, 1.00]	1.00 [0.00, 1.00]	3e-06 [0.00, 0.97]
